# Evaluating the Impact of Zimbabwe’s Prevention of Mother-to-Child HIV Transmission Program: Population-Level Estimates of HIV-Free Infant Survival Pre-Option A

**DOI:** 10.1371/journal.pone.0134571

**Published:** 2015-08-06

**Authors:** Raluca Buzdugan, Sandra I. McCoy, Constancia Watadzaushe, Mi-Suk Kang Dufour, Maya Petersen, Jeffrey Dirawo, Angela Mushavi, Hilda Angela Mujuru, Agnes Mahomva, Reuben Musarandega, Anna Hakobyan, Owen Mugurungi, Frances M. Cowan, Nancy S. Padian

**Affiliations:** 1 School of Public Health, University of California, Berkeley, California, United States of America; 2 Centre for Sexual Health and HIV/AIDS Research Zimbabwe, Harare, Zimbabwe; 3 School of Medicine, University of California San Francisco, San Francisco, California, United States of America; 4 Ministry of Health and Child Care, Harare, Zimbabwe; 5 University of Zimbabwe, Harare, Zimbabwe; 6 Elizabeth Glaser Pediatric AIDS Foundation, Harare, Zimbabwe; 7 Children’s Investment Fund Foundation, London, United Kingdom; 8 Research Department of Infection and Population Health, University College London, London, United Kingdom; National Institute of Health, ITALY

## Abstract

**Objective:**

We estimated HIV-free infant survival and mother-to-child HIV transmission (MTCT) rates in Zimbabwe, some of the first community-based estimates from a UNAIDS priority country.

**Methods:**

In 2012 we surveyed mother-infant pairs residing in the catchment areas of 157 health facilities randomly selected from 5 of 10 provinces in Zimbabwe. Enrolled infants were born 9–18 months before the survey. We collected questionnaires, blood samples for HIV testing, and verbal autopsies for deceased mothers/infants. Estimates were assessed among i) all HIV-exposed infants, as part of an impact evaluation of Option A of the 2010 WHO guidelines (rolled out in Zimbabwe in 2011), and ii) the subgroup of infants unexposed to Option A. We compared province-level MTCT rates measured among women in the community with MTCT rates measured using program monitoring data from facilities serving those communities.

**Findings:**

Among 8568 women with known HIV serostatus, 1107 (12.9%) were HIV-infected. Among all HIV-exposed infants, HIV-free infant survival was 90.9% (95% confidence interval (CI): 88.7–92.7) and MTCT was 8.8% (95% CI: 6.9–11.1). Sixty-six percent of HIV-exposed infants were still breastfeeding. Among the 762 infants born before Option A was implemented, 90.5% (95% CI: 88.1–92.5) were alive and HIV-uninfected at 9–18 months of age, and 9.1% (95%CI: 7.1–11.7) were HIV-infected. In four provinces, the community-based MTCT rate was higher than the facility-based MTCT rate. In Harare, the community and facility-based rates were 6.0% and 9.1%, respectively.

**Conclusion:**

By 2012 Zimbabwe had made substantial progress towards the elimination of MTCT. Our HIV-free infant survival and MTCT estimates capture HIV transmissions during pregnancy, delivery and breastfeeding regardless of whether or not mothers accessed health services. These estimates also provide a baseline against which to measure the impact of Option A guidelines (and subsequently Option B+).

## Introduction

Pediatric HIV/AIDS remains a major global public health issue, with 330,000 children newly infected with HIV in 2011, 90% of whom lived in Sub-Saharan Africa.[1] In 2011 UNAIDS launched a global plan to eliminate new pediatric HIV infections by 2015, which focused on twenty-two priority countries where 90% of the world’s HIV-infected pregnant women reside.[2] To this end, the World Health Organization (WHO) revised their 2006 guidelines for the elimination of mother-to-child HIV transmission (eMTCT) in developing countries. Specifically, in 2010 WHO recommended that HIV-infected pregnant women eligible for antiretroviral therapy (ART) receive lifelong therapy, while ART-ineligible women receive one of two antiretroviral (ARV) prophylactic regimens (Options A or B) to prevent MTCT during pregnancy and breastfeeding.[3] Compared to the earlier guidelines, the 2010 guidelines recommended lifelong ART for a larger group of HIV-infected women (i.e., CD4≤350 or clinical stage 3–4 vs. CD4≤200), and that ARV prophylaxis be provided earlier in the pregnancy (i.e., starting at 14 weeks instead of 28). In addition, for the first time, ARVs to prevent MTCT during breastfeeding were recommended in settings where breastfeeding is the safest feeding option. In 2013, WHO updated their guidelines again, recommending that all pregnant women, regardless of clinical stage, receive ART at a minimum during pregnancy and breastfeeding (Option B) or ideally lifelong (Option B+).[4]

Although many developing countries have adopted the 2010 guidelines or are beginning to adopt the 2013 WHO-recommended regimens,[5] little is known about their population-level effectiveness (regimen efficacy combined with adherence and program coverage).[6] Recognizing that facility-based estimates of impact may overestimate the true effect of eMTCT regimens as they only include mothers who accessed health services, WHO has urged countries to conduct population-level impact assessments of their eMTCT programs, with rates of MTCT and HIV-free infant survival, ideally measured at 18 months, used as the main outcomes to determine impact.[7] HIV-free infant survival is considered the gold standard, as it accounts for deaths prevented and infant infections at birth and during breastfeeding.[8]

Zimbabwe, a UNAIDS priority country, has an estimated 1.4 million people living with HIV infection. Modeling studies conducted between 2009 and 2011 estimated that MTCT ranged between 17% and 30%.[9–12] In 2011, Zimbabwe’s Ministry of Health and Child Care (MoHCC) implemented Option A of the 2010 WHO guidelines. Specifically, MoHCC rolled out this program in 1344 of the existing 1,560 health facilities in the country between August and December 2011, distributed point-of-care CD4 testing machines for determination of ART eligibility, and facilitated community mobilization to increase entry and retention in the PMTCT cascade; UNAIDS commended the Zimbabwe MoHCC for this successful countrywide implementation.[13] Zimbabwe began rollout of Option B+ in November 2013.

Well in advance of the 2013 WHO guidelines and working with the MoHCC, we designed an evaluation consisting of serial cross-sectional community-based surveys to determine the impact of Option A on HIV-free infant survival and MTCT at 9–18 months postpartum in Zimbabwe. Here we present estimates of HIV-free infant survival and MTCT based on our baseline survey, conducted in May-September 2012. We calculated these estimates among all HIV-exposed infants, irrespective of potential exposure to Option A. We also assessed these rates in the subgroup of infants who were not exposed to Option A. Lastly, we compared province-level MTCT rates based on the community survey with MTCT rates based on monitoring and evaluation data from facilities in the same provinces, to verify the assumption underlying WHO recommendations that facility-based estimates of impact are likely to overestimate the effectiveness of eMTCT regimens.

## Methods

**Fig 1:**
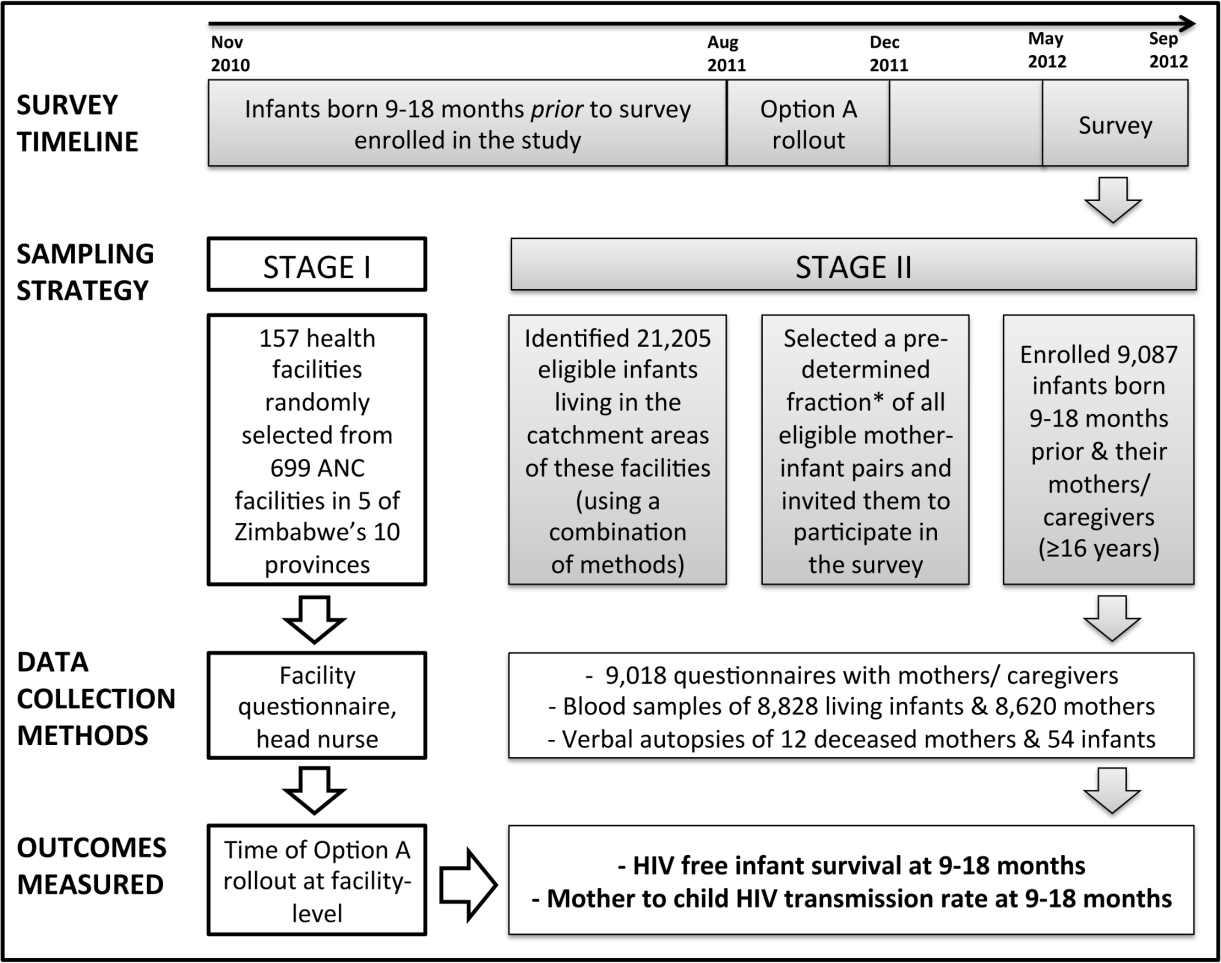
Survey timeline, sampling strategy, data collection methods and outcomes measured for the 2012 survey of the impact evaluation of Zimbabwe’s Accelerated PMTCT program. * In each catchment area, we used one of the following sampling fractions (so that we enroll an average of 50 mother-infant pairs per catchment area, which was one of the assumptions of our power calculations): 1) 1 in 4 eligible mother-infant pairs if the catchment area had over 300 eligible infants, 2) 1 in 2 infants if the catchment area had 150 to 300 eligible infants, or 3) all the mother-infant pairs if the catchment area had fewer than 150 eligible infants.

We conducted a cross-sectional survey of mother-infant pairs selected in two stages. First, we randomly selected 157 health facilities from the 699 facilities offering antenatal care in five of Zimbabwe’s ten provinces. Second, we identified 21,205 eligible mother-infant pairs living in the catchment areas of these 157 facilities, selected a pre-determined fraction of them and invited them to participate in the survey. Infants born 9–18 months prior to the survey and their mothers/ caregivers were eligible for the survey. Overall, 9,087 mother-infant pairs participated in the 2012 survey. We conducted questionnaires with mothers/ caregivers, verbal autopsies regarding the deceased mothers/infants, and collected maternal and infant blood samples for HIV testing. In addition, we administered a short questionnaire to head nurses in the 157 health facilities. See Fig 1 for a brief summary of the study design. Below we discuss and explain in detail the study population, sampling strategy, data collection methods, laboratory procedures, data analysis, and the measures for the protection of human subjects.

### Study population

**Fig 2:**
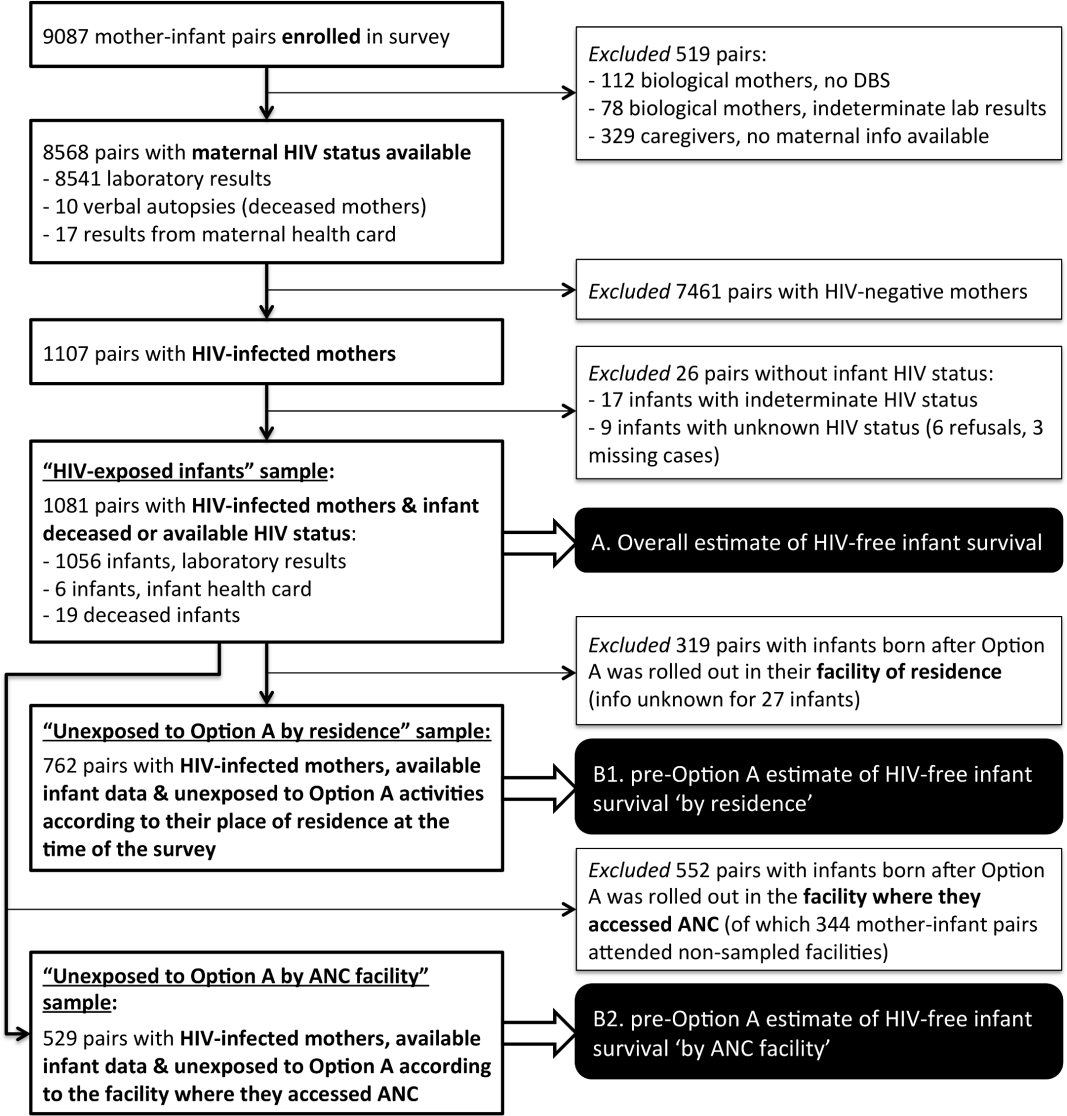
Samples of mother-infant pairs used to calculate three estimates of HIV-free infant survival at 9–18 months, Zimbabwe, 2012. The flowchart summarizes what samples were used to estimate HIV-free infant survival at 9–18 months. The overall estimate of HIV-free infant survival (‘estimate A’) was assessed among the ‘HIV-exposed infants’ sample (i.e., 1,081 pairs with HIV-infected mothers and infants either deceased or with available HIV status). The pre-Option A estimates of HIV-free infant survival (‘estimates B1 and B2’) were assessed among subsets of the ‘HIV-exposed infants’ sample, after excluding the infants who were exposed to Option A activities. We measured exposure to Option A activities in two ways: 1) according to the facility closest to the infant’s residence at the time of the survey, and 2) according to the facility where antenatal care (ANC) was received. These pre-Option A samples of infants were used to assess HIV-free infant survival using two definitions of exposure: ‘by residence’ (‘estimate B1’) and ‘by ANC facility’ (‘estimate B2’).

The study population consisted of infants born 9–18 months before the survey and their mothers or caregivers (≥16 years old). The infants’ age range allowed us to capture HIV transmissions occurring during pregnancy, delivery, and breastfeeding.[3] Given the high maternal mortality associated with HIV infection[14] and the high mortality among HIV-infected infants,[15] we included deceased mothers and infants. Overall, 9,087 mother-infant pairs participated in the survey; 68 infants and 55 mothers were deceased at the time of the survey. Fig 2 explains in detail the samples used in the analyses conducted for this paper.

### Sampling strategy

Resources precluded sampling the entire country. Hence, our target population was mother-infant pairs residing in five of Zimbabwe’s ten provinces (Harare, Mashonaland West, Mashonaland Central, Manicaland, Matabeleland South), selected to include three of the four largest cities in Zimbabwe, rural communities with high and low HIV prevalence, representation of both major ethnic groups in Zimbabwe (Shona, Ndebele), and areas where detailed monitoring and evaluation data were being collected. Harare is the capital and an urban metropolis, whereas the other four provinces include both urban and rural populations.

First, we randomly selected 157 of 699 health facilities offering eMTCT services in these provinces and administered a questionnaire with head nurses to capture the services delivered at that facility and the timing of Option A rollout. We selected as primary sampling units the catchment areas (CAs) of these facilities. (In this study we used the health facility catchment areas, which are geographical areas defined by the MoHCC. CAs are defined by boundaries of the administrative ward(s), which are subdivisions of districts.) CAs were randomly sampled proportionate to the number of facilities in their district. In each sampled CA, we identified all eligible infants (i.e. infants born 9–18 months before the survey and their ≥16 years old mothers or caregivers) and sampled a known fraction of these infants with the objective of enrolling 50 infants per CA. Specifically, in each CA we used one of the following sampling fractions: 1) 1 in 4 eligible mother-infant pairs if the CA was estimated to have over 300 eligible infants, 2) 1 in 2 infants if the CA was estimated to have 150 to 300 eligible infants, or 3) all the mother-infant pairs if the CA was estimated to have fewer than 150 eligible infants. Sample size was calculated to adequately power the impact evaluation.

Infants born in the previous two years were identified based on information pooled from: 1) community health workers (CHWs) and 2) immunization registers from selected facilities and neighboring facilities (to identify women residing in sampled facilities who accessed services at adjacent facilities). Further, mothers identified using (1) and (2) were asked to identify other eligible infants in their neighborhood. This three-pronged approach efficiently identified eligible participants without screening all the households in the selected CAs and captured mother-infant pairs who received care outside their area of residence.

### Data collection

Participating mothers/caregivers completed anonymous interviewer-administered questionnaires that captured the mother’s demographic characteristics, experience with antenatal and postnatal care, delivery, ART and ARV prophylaxis for the eligible child. When participants had patient-held medical records in their possession (i.e., infant health card, maternal health card), interviewers collected information on HIV status and treatment recorded on these documents. In addition, all living biological mothers and infants provided dried blood spot samples (DBS) for HIV testing, following written informed consent. If the biological mother of an eligible infant was deceased, we interviewed the caregiver to assess whether HIV/AIDS was the probable cause of the maternal death. Similarly, if the eligible infant was deceased at the time of the survey, we interviewed the biological mother to assess the baby’s likely cause of death. To this end, we used verbal autopsy questionnaires developed by the WHO and adapted them for our population.

### Laboratory procedures

Blood samples were air-dried onto filter papers and stored at room temperature until transported biweekly to the National Microbiology Reference Laboratory in Harare. Maternal samples were tested for HIV-1 antibody using AniLabsytems EIA kit (AniLabsystems Ltd, OyToilette 3, FIN-01720) with all positive specimens confirmed using Enzygnost Anti-HIV 1/2 Plus ELISA and discrepant results resolved by Western Blot. We tested the samples of the infants born to HIV-positive mothers as well as the infants born to mothers whose sample was unavailable; infant samples were tested for HIV with DNA polymerase chain reaction (Roche Amplicor HIV-1 DNA Test 1.5). External quality assurance was conducted on 7% of samples.

### Data analysis

#### Primary outcomes

We estimated two outcomes:

1. HIV-free infant survival (proportion of infants born to HIV-infected mothers who were alive and HIV-uninfected at 9–18 months of age): The denominator (number of HIV-infected mothers) was assessed based on either i) laboratory-confirmed HIV test results (99.4%), ii) verbal autopsy data (for deceased mothers, 0.3%), or iii) information recorded on maternal health cards (for deceased or unavailable mothers, 0.3%). To classify deaths as due to AIDS from verbal autopsy data, we used an algorithm validated in Zimbabwe.[16] The numerator (number of living HIV-uninfected infants) was assessed based on i) laboratory-confirmed HIV test results (97.7%), ii) information recorded on infant health cards (1.8%), and iii) reports of infants’ deaths (0.5%).
2. MTCT (proportion of infants born to HIV-infected mothers who were HIV-infected at 9–18 months of age) uses the same denominator as HIV-free infant survival. The numerator (number of infants HIV-infected or deceased related to HIV/AIDS) was assessed based on i) laboratory-confirmed HIV test results (98.1%), ii) verbal autopsy data (for deceased infants, 1.3%), and iii) information recorded on infant health cards (0.6%). A Zimbabwean pediatrician (HAM) examined the infant verbal autopsy data and rated the likelihood of each death being HIV-related (on a 5-point scale ranging between ‘very unlikely’ and ‘very likely’) based on: gestational age at delivery, birth weight, infant age at death, symptoms indicative of common opportunistic infections in children, along with the chronicity of their illness and factors that affect likelihood of MTCT. ‘Likely’ and ‘very likely’ cases were classified as infant HIV/AIDS-related deaths.

#### Assessment of exposure to Option A

We defined exposure to Option A in two ways. First, mother-infant pairs were considered exposed to Option A activities if infants were born *after* Option A guidelines were implemented in the *facility closest to their place of residence* at the time of the survey. (Note that the impact evaluation was designed based on this definition of exposure to Option A, as the national PMTCT program was implemented at the facility level). Although the order in which communities were surveyed was timed to maximize the likelihood that all enrolled infants were born *before* program rollout, we identified some infants born after Option A was implemented in the closest facility. The timing of Option A rollout was unavailable for two sampled facilities.

Second, mother-infant pairs were considered exposed to Option A activities if infants were born *after* Option A guidelines were implemented in the *facility where their mothers reported to have received antenatal care*. This alternative definition of exposure to Option A acknowledges that not everyone sought care at the facility closest to their residence and captures mobility in seeking care. Although more precise than the definition of exposure to Option A based on the nearest facility, assessment of the facility where the mother received antenatal care was only possible for the subset of women who attended prenatal care at the 155 facilities in our sample where we could verify the exact timing of Option A rollout; we used this second definition of exposure to Option A in sensitivity analyses (see below).

#### Statistical analysis

We present descriptive statistics of facility- and individual-level characteristics and the primary outcomes. For each primary outcome (defined above), we provide three sets of estimates (see Fig 2 for details on the samples used to generate each set of estimates). First, we assessed overall, community-based estimates (‘estimate A’) among the sample of infants born to HIV-infected mothers, irrespective of potential exposure to Option A. Second, we restricted these estimates to infants born *before* Option A was implemented in the facility closest to their residence at the time of the survey (‘estimate B1’). Third, we limited the estimates to infants born *before* Option A was rolled out in the facility where they reportedly received antenatal care (‘estimate B2’), to examine the sensitivity of the B1 estimates to potential mobility during pregnancy. In other words, for estimates B1 and B2 we *excluded* infants exposed to Option A activities.

We also compared the community-based estimate of MTCT (‘estimate A’) measured with the community serosurvey to the MTCT rate measured with facility-based monitoring and evaluation data collected by the National Health Information Unit of the MOHCC. We limited this comparison to MOHCC estimates from facilities serving the communities included in survey, and examined possible differences in province-level estimates by source of data. The facility-based data represent the results of all routine HIV tests conducted in 2012 among HIV-exposed infants up to 18 months (most of these infants were <4 months old). Each health facility sends the infant blood samples to a central collection point, where they are batched with other specimens and sent weekly to the National Microbiology Reference Laboratory to be tested for HIV with DNA polymerase chain reaction.

All the analyses of the community-based survey data were conducted using the software package STATA version 12 (StataCorp, Texas). The data were weighted to account for the two-stage stratified cluster design and survey non-response; thus, we estimated the above-mentioned key outcomes using STATA *svy* commands.

### Human subjects protection

The protocol was approved by the Medical Research Council of Zimbabwe and the ethics committees of University of California, Berkeley and University College London. All participants provided written informed consent prior to participation and were compensated for their time with a gift worth $5. The study includes infants who provided dried blood spots samples for HIV testing; collection of the dried blood spots samples was only conducted after written informed consent from their mothers/ caregivers was obtained. In addition, some of the mothers were 16 to 21 year old; according to the Medical Research Council of Zimbabwe, these mothers are emancipated minors capable of providing informed consent for themselves and their children’s participation in a research study. Participants could retrieve their anonymous HIV test results at the local facility up to 3 months following the survey, using a card with barcode numbers.

## Results

We sampled a diverse group of 157 facilities (Table 1). Most facilities offered key eMTCT services on-site, including antenatal care (n = 156), HIV testing (n = 156), maternal (n = 152) and infant ARV prophylaxis (n = 154), postnatal care (n = 157) and immunizations (n = 156), with delivery (n = 139) and on-site CD4 testing (n = 96) being the least available services.

**Table 1:** Characteristics of sampled health facilities in five provinces, Zimbabwe, 2012.

	Number of health facilities	Frequency distribution of health facilities
	(n = 157)	(%, out of 100%)
**Type of facility**		
Central / provincial / district hospital	6	3.8
Mission / rural health hospital	15	9.6
Local authority	3	1.9
Private hospital	2	1.3
Rural health clinic	96	61.2
Polyclinic	9	5.7
Satellite clinic	4	2.6
Other (mostly clinics)	22	14.0
**Estimated population in health facility catchment area**		
<5000	39	24.8
5000–9999	60	38.2
10000–14999	33	21.0
15000+	17	10.8
Don't know	8	5.1
**Number of rooms in facility**		
Mean (standard deviation)	145	9.0 (10.3)
**Staff members in facility (except village health workers)**		
Mean (standard deviation)	153	14.1 (56.2)
**Number of village health workers / health promoters**		
Mean (standard deviation)	150	7.3 (7.2)
**Link with communities** (multiple choice question)		
No link to the community	1	0.6
Community health nurse	23	14.7
Village health worker	129	82.2
Home based care cadre	10	6.4
Outreach teams	5	3.2
Environmental health officer	16	10.2
Other	40	25.5
**Services offered during the month prior to the survey**		
Antenatal care	156	99.4
HIV testing for pregnant women	156	99.4
Maternal ARV prophylaxis	152	96.8
Infant ARV prophylaxis	154	98.1
Labor and delivery	139	88.5
Postnatal care	157	100.0
Immunizations for infants	156	99.4
CD4 testing on-site	96	61.2

Of the 9184 eligible mother-infant pairs selected from the sampled CAs, we enrolled 9087 pairs (98.9% response rate) who participated in at least one survey component (questionnaire, maternal DBS, or infant DBS). Overall, 8568 mothers had an HIV result and 1107 (12.9%, 95% confidence interval (CI): 12.2–13.6) were HIV-infected. Twenty-seven infants (2.5% of HIV-exposed infants) were sampled from catchment areas where we could not assess exposure to Option A. The appendix illustrates the overlap between the samples of mother-infant pairs used to estimate B1 (n = 762) and B2 (n = 529).

Table 2 provides the socio-demographic profile of HIV-infected mothers (sample used to generate estimate A), and of HIV-infected mothers with infants unexposed to Option A. Because the profiles of Option A-unexposed mothers assessed using definition B1 (i.e., whether Option A guidelines were implemented in the facility closest to the residence at the time of the survey) are statistically indistinguishable from Option A-unexposed mothers using definition B2 (whether Option A guidelines were implemented in the facility where they reportedly received antenatal care), here we present the profile of the B1 sample only (as that is the baseline of our impact evaluation). The socio-demographic profiles of the two samples of HIV-infected mothers were very similar. HIV-infected mothers were mostly married (81%) and had multiple births (86%). Most of them lived in large households (79% with ≥4 members) that reported having experienced some household food insecurity (61%). Only 29% of mothers had finished high school and 23% were employed.

**Table 2:** Socio-demographic characteristics of mother-infant pairs enrolled in the baseline survey, for specific subpopulations (unweighted N and %), Zimbabwe, 2012.

	‘HIV-exposed infants’ and their mothers, irrespective of whether they were exposed to Option A or not (sample A)		Infants ‘unexposed to Option A activities based on their residence’ and their mothers (sample B1)	
	N	%	N	%
	(n = 1075)*		(n = 759)*	
**Mothers’ age**				
16–20 years	67	6.3	51	6.8
21–25 years	264	24.8	184	24.6
26–30 years	323	30.4	229	30.6
31–35 years	268	25.2	183	24.4
36+ years	141	13.3	102	13.6
**Mothers’ ethnicity**				
Shona	757	71.2	556	74.2
Ndebele	163	15.3	97	13.0
Other	143	13.5	96	12.8
**Mothers’ marital status**				
Married	863	81.2	596	79.6
Previously married	143	13.5	110	14.7
Never married	57	5.4	43	5.7
**Mothers’ parity**				
1 birth	150	14.2	116	15.5
2 births	269	25.4	188	25.2
3 births	265	25.0	185	24.8
4+ births	376	35.5	258	34.5
**Mothers’ education**				
Primary or less	424	39.9	294	39.3
Form 1–3	337	31.7	231	30.8
Form 4	293	27.6	217	29.0
Form 5+	9	0.9	7	0.9
**Household size**				
1–3 people	223	21.0	170	22.7
4 people	245	23.1	160	21.4
5 people	233	21.9	167	22.3
6 people	152	14.3	102	13.6
7+ people	210	19.8	150	20.0
**Household food insecurity**				
Severe insecurity	303	28.5	205	27.4
Some insecurity	343	32.3	255	34.1
Food secure	417	39.2	289	38.6
**Mothers employed**	246	23.3	172	23.2
**Infants alive or deceased**				
Deceased	19	1.8	13	1.7
Alive	1050	98.2	739	98.3
*Among alive infants*	(n = 1050)		(n = 739)	
**Infants’ age**				
9–10 months	235	22.4	65	8.8
11–12 months	238	22.7	148	20.0
13–14 months	222	21.1	195	26.4
15–16 months	196	18.7	179	24.2
17–18 months	159	15.1	152	20.6

* Sample size differs from the one listed in Fig 2, as i) this table only examines data regarding biological mothers and ii) some of the biological mothers included in the estimate of HIV-free infant survival did not answer the questionnaire.

**Fig 3:**
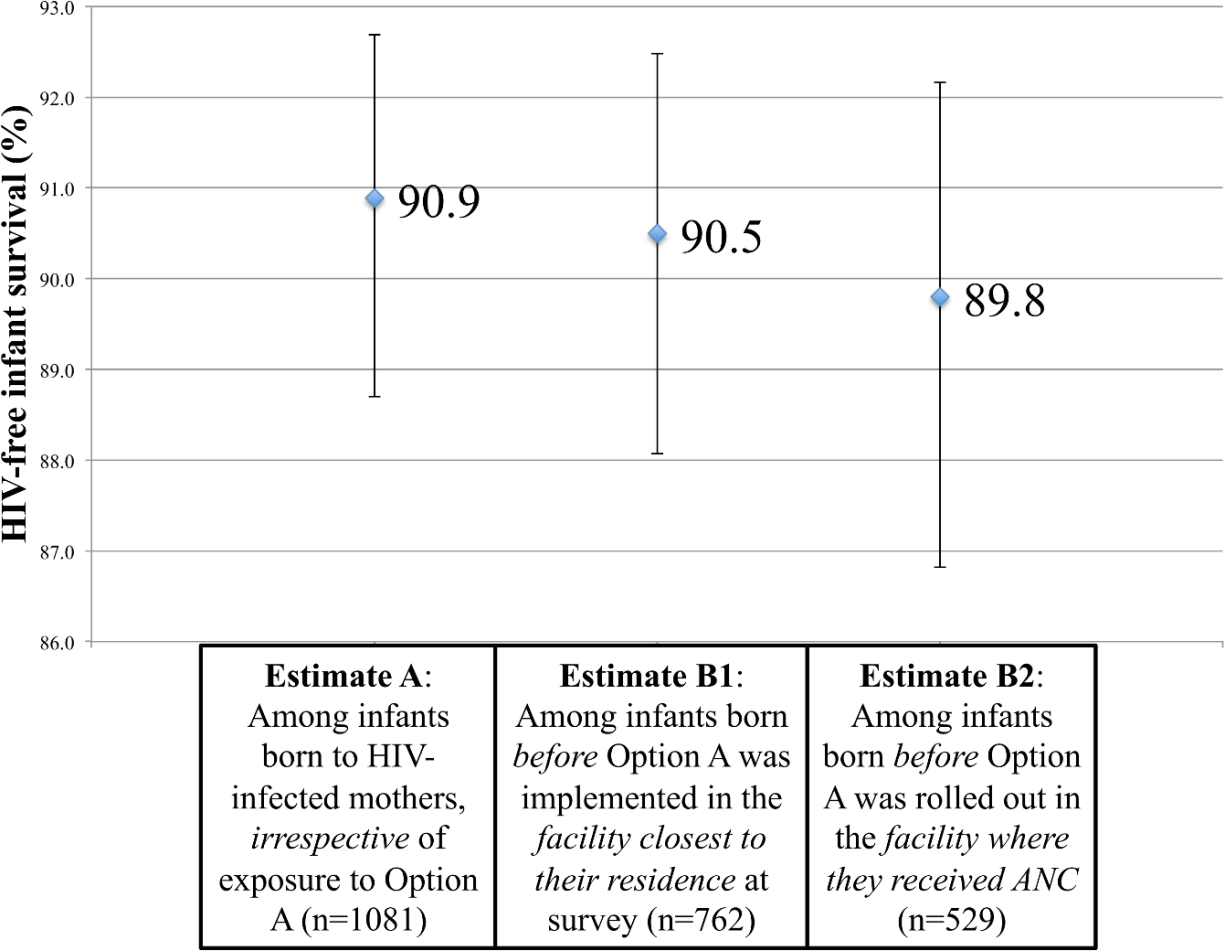
HIV-free infant survival at 9–18 months of age in five provinces in Zimbabwe, 2012. The figure shows estimates and 95% confidence intervals adjusted for the survey design. Weighted frequencies are shown on the x-axis. The sample size (unweighted) differs from that listed on Fig 4 due to missing data regarding the infants’ status for a few mother-infant pairs.

**Fig 4:**
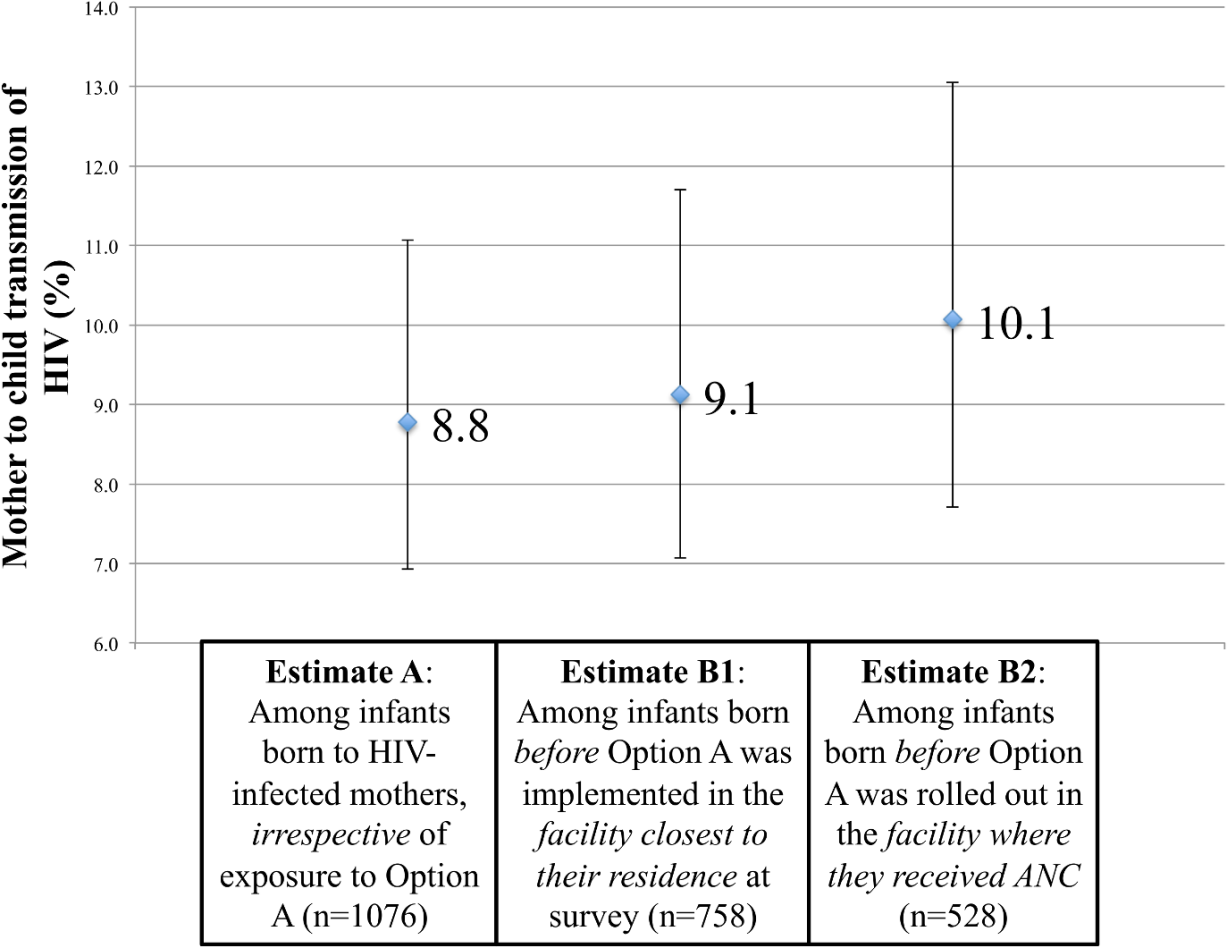
Mother-to-child HIV transmission rate at 9–18 months of age in five provinces in Zimbabwe, 2012. The figure shows estimates and 95% confidence intervals adjusted for the survey design. Weighted frequencies are shown on the x-axis. The sample size (unweighted) differs from that listed on Fig 3 due to missing data regarding the infants’ status for a few mother-infant pairs.

Fig 3 presents the estimates of HIV-free infant survival and Fig 4 presents MTCT at 9–18 months of age. Among the sample of infants born to HIV-infected mothers, irrespective of potential exposure to Option A, HIV-free infant survival was 90.9% (95% CI: 88.7–92.7) and MTCT was 8.8% (95% CI: 6.9–11.1) (estimates A). Among the infants born before Option A was implemented in the facility closest to their residence at the time of the survey, 90.5% (95% CI: 88.1–92.5) of infants born to HIV-infected mothers were alive and HIV-uninfected at 9–18 months of age; 9.1% (95% CI: 7.1–11.7) of them were HIV-positive (estimates B1). Sensitivity analyses among the sample of infants born before Option A was rolled out in the facility where their mothers reportedly received antenatal care yielded an estimate of HIV-free infant survival of 89.8% (95% CI: 86.8–92.2) and MTCT of 10.1% (95% CI: 7.7–13.1).

**Fig 5:**
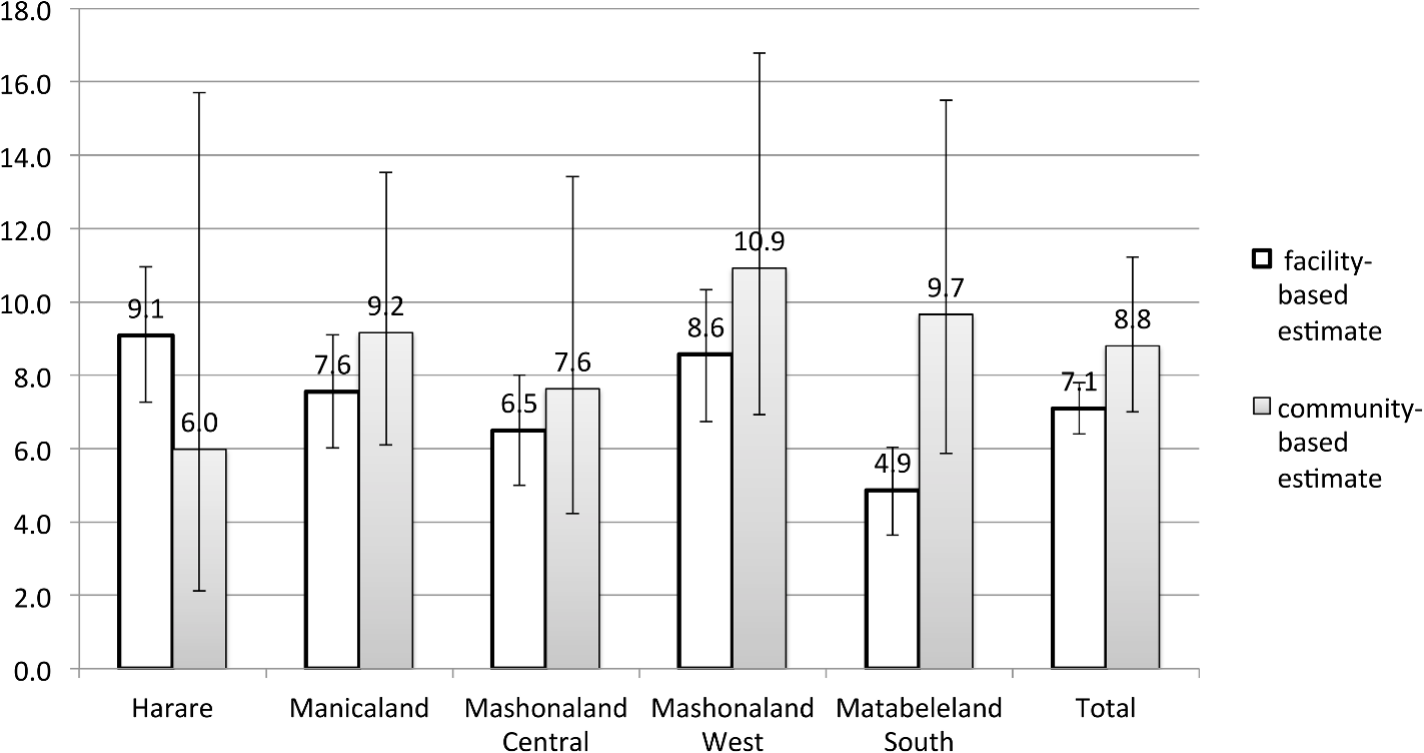
Mother-to-child HIV transmission rate by province, estimated by the community survey and by Zimbabwe’s National Health Information System using facility-based monitoring and evaluation data. The community survey was conducted in May-September 2012 and enrolled babies aged 9 to 18 months, while the facility-based data consists of early infant testing conducted in January-December 2012 among babies mostly <4 months.

Fig 5 compares province-level MTCT estimates from the community-based survey (estimate A) and national monitoring and evaluation data from facilities serving those communities. Overall, the MTCT rate based on the program data is slightly lower than based on the community-based data (7.1% vs. 8.8%, p = 0.063). In four of the five provinces examined, the community-level estimates of MTCT are higher than the facility-based estimates; the opposite is the case in Harare.

## Discussion

We estimate that in 2012, 90.9% of HIV-exposed infants in five provinces in Zimbabwe were alive and HIV-uninfected at 9–18 months of age, and 8.8% were HIV-infected. Our two estimates of HIV-free infant survival and MTCT prior to the implementation of Option A were very similar to each other and comparable to the overall rates (irrespective of exposure to Option A). These estimates, among the first community-based estimates from a UNAIDS priority country, capture HIV transmissions during pregnancy, delivery and the first 9–18 months of breastfeeding among HIV-exposed mother-infant pairs regardless of whether they attended eMTCT services. Although much lower than previous estimates,[9–12] as of 2012 there was nevertheless a substantial gap between this transmission rate and the eMTCT goal of <5% MTCT.

We compared the MTCT estimates from our community-based survey with facility-based estimates based on data from Zimbabwe’s National Health Information System. With the exception of facilities in Harare, community-based estimates in each province were higher than facility-based estimates from the same province. Notably, the community MTCT rate is from the serosurvey conducted in May-September 2012 among babies aged 9 to 18 months, while the facility MTCT rate is from early infant testing conducted in January-December 2012 among babies mostly under 4 months. Thus, the higher MTCT rates we observe in four of the five provinces may be due in part to the fact that the community serosurvey captures both early and late MTCT transmissions. In addition, mothers who were not retained in the eMTCT cascade may be less likely to return to a facility for early infant testing and might be expected to have higher rates of MTCT. Data from four provinces support the assumption that community-based estimates of MTCT are likely to be more representative than facility-based estimates based on early infant testing. However, in Harare, program data suggest a higher MTCT rate than the community survey, possibly because many high-risk women come in from rural areas to give birth in Harare to receive high quality healthcare in case of complications. Further, although many women who live in Harare go to their parents’ home in rural areas for the birth and postpartum period, it is likely that women who know they are at high-risk will be less likely to travel in order to access the better quality healthcare available in Harare. As such higher risk women may be overrepresented in Harare facilities, while the community survey likely includes both low-risk and high-risk women.

Unlike our survey, most eMTCT effectiveness studies have been facility-based. Facility-based designs have significant drawbacks. Loss to follow-up (LTFU) and incomplete and inconsistent record keeping are major concerns in analyses of existing program data.[6] Cohort studies among women recruited from facilities are also sensitive to LTFU and bias due to selection into the cohort or to the care and follow-up they receive.[6] Surveys conducted among mother-infant pairs attending childhood immunizations only provide MTCT estimates of early transmission and do not account for breastfeeding-related transmission.

In contrast, community-based serosurveys such as ours [17,18] can estimate MTCT and HIV-free infant survival post-breastfeeding, and capture information about mothers not accessing eMTCT services. There are examples of similar designs. Serial community-based survey data showed that a pilot program implementing Option B in four Zambian facilities increased HIV-free survival at 24 months (from 66% at baseline to 89% post-intervention, adjusted hazard ratio 0.52).[17] The four-country PEARL study, which is currently underway, is also using a community-based design to assess eMTCT effectiveness; in 2007–2009 this study estimated HIV-free survival at 24 months to be 73% in Cameroon, 78% in South Africa, 83% in Zambia, and 84% in Cote D’Ivoire.[18]

Over the past decade there have been substantial reductions in MTCT at 6 weeks as a result of eMTCT interventions; rates fell in Malawi (26.6% in 2004, 15.5% in 2006,[19] 4.1% in 2009–2011,[20] 8.5% in 2011[21]) and in South Africa (20% in 2004–2005,[22] 10% in 2009,[23] 3.5% in 2010,[24] 2.7% in 2011[25]). However, HIV-free infant survival provides more robust evidence of programmatic effectiveness than MTCT rates. HIV-free infant survival ranges from 66% at 18–20 months in Malawi (2009 facility-based cohort data)[26] to 91.9% at 9–24 months in Rwanda (2009 nationwide cross-sectional survey prior to Option B rollout).[27] Our estimates, based on community-based survey data, suggest that Zimbabwe has made considerable progress towards elimination of MTCT, with 91% of infants born to HIV-infected mothers remaining alive and HIV-uninfected at 9–18 months of age even before rollout of Option A.

Our community-based findings will serve as baseline estimates for the impact evaluation of Option A and Option B+ in Zimbabwe. These data will be triangulated with facility-based data from early infant testing (as we have done in this paper) as well as an ongoing impact assessment study, which prospectively follows mother-infant pairs identified at the 6–8 week immunization visit. Together, these studies will provide the MOHCC with a comprehensive understanding of the population-level effectiveness of Options A and B+, and answers to operational research questions. Although global standards have moved towards Option B+, its implementation and costs are not without challenges; hence understanding the population-level effectiveness of Option A remains informative.

Our study is subject to limitations. Although our estimates account for transmissions occurring during the first 9–18 months of breastfeeding, 66% of HIV-exposed infants were still breastfeeding at the time of the survey (median duration of breastfeeding is 17.8 months in Zimbabwe[14]). Thus, HIV-free survival at 24 months (at the end of breastfeeding) could be lower, and MTCT might be higher. Nonetheless, estimates restricted to all HIV-exposed infants no longer breastfeeding at the time of the survey were comparable: 91.9% (95% CI: 86.8–95.1) HIV-free infant survival and 8.1% (95% CI: 4.9–13.2) MTCT (data not shown). Further, despite our efforts to enroll all eligible mother-infant pairs and rely on verbal autopsy data, infant deaths may have been underreported.

Moreover, although some sampled clinics did not offer the entire cascade of eMTCT services, because of the complexity of matching the services offered at the clinic level with the needs of each mother-infant pair, we did not take this into account when attributing exposure to Option A. Finally, data were only collected in five of ten provinces, although these were widely dispersed across the country and included major cities. Nevertheless, our findings are consistent with national-level data from the 2010–2011 ZHDS that similarly estimated that 12% of pregnant women were HIV-infected.[14]

In sum, we found that by 2012 when Option A was implemented in Zimbabwe, the country had made substantial progress towards the elimination of MTCT. Our HIV-free infant survival and MTCT estimates compliment the facility-based findings of early transmission to capture HIV transmissions during pregnancy, delivery and the first 9–18 months of breastfeeding regardless of whether or not mothers accessed health services. Our findings inform the progress and remaining challenges of Zimbabwe’s national eMTCT program.

## Supporting Information

S1 Fig Overlap between the samples to estimate pre-Option A HIV-free infant survival at 9–18 months: estimates B1 and B2, Zimbabwe, 2012. The figure shows the overlap between the samples used to estimate B1 (n = 762) and B2 (n = 529), defined in Fig 2. Overall, 522 infants were categorized as unexposed to Option A according to both definitions of Option A exposure (and were included in the estimates B1 and B2). In addition, 240 infants were classified as unexposed to Option A based on data from the facility closest to their residence (and were only included in the estimate B1), and 7 infants were considered Option A-unexposed if Option A exposure was defined by where ANC was received (and were only included in the estimate B2).
(DOCX)

S1 File Household questionnaire administered to mother/caregiver of eligible infant (DOCX)

S2 File Facility-based questionnaire administered to head nurse. (DOCX)

S3 File Consent form for participating mother/ caregivers. (DOCX)

S4 File Consent form for participating head nurses. (DOCX)

S5 File Database – 2012 cross-sectional survey. (DTA)
