# Supplementary material for: Evaluating the Impact of Zimbabwe’s Prevention of Mother-to-Child HIV Transmission Program: Population-Level Estimates of HIV-Free Infant Survival Pre-Option A
Source: PLoS One. 2015 Aug 6;10(8):e0134571. doi: 10.1371/journal.pone.0134571 (PMC4527770; doi:10.1371/journal.pone.0134571)
Supplement: S1 File — (DOCX) [file pone.0134571.s002.docx]

**Zimbabwe PMTCT Evaluation – Baseline Survey Questionnaire**

Captured automatically by CAPI: date of interview, time interview started and ended, language of interview

The survey assistant should enter: participant ID, clinic catchment area

Color codes: inserted picture

| **Q** | **Question** | **Answers** | **Instructions** | **Skip** |
| --- | --- | --- | --- | --- |
| Intro Q1 | The following questions should be answered by the survey assistant based on the information collected on the Registration form. |  |  |  |
| Q1 | To verify eligibility of the infant, please enter the birth date of the selected infant (dd/mm/yyyy). | Number pad | Please use the number pad | If survey date - Q1={9,18m} go to Q2  If survey date - Q1≠ {9,18m} STOP |
| Q2 | To verify eligibility of the participant, please enter the mother/caregiver’ age at the last birthday. | Number pad | Please use the number pad | If Q2>15 go to Q3  If Q2≤15 STOP |
| Q3 | Is the baby alive or deceased? | Alive (1)  Deceased (0) | Please press on one box only | Go to Q4 |
| Q4 | Is the respondent the selected infant’s biological mother? | Yes (1)  No (0) | Please press on one box only | If Q3=0 and Q4=0 STOP  If Q3=1 and Q4=0 go to Q5  If Q4=1 and Q3=0 go to A2b  If Q4=1 and Q3=1 go to A2a |
| Q5 | Enter respondent’s gender. | Female (1)  Male (0) | Please press on one box only | Go to A1 |
|  | **Section A. Antenatal care** |  |  |  |
| Intro A1 | In this questionnaire we will **only** ask you questions about your child born on [DATE]. |  | To the survey assistant: START ADRESSING THE MOTHER |  |
| A1 | Does this baby’s biological mother live in this household? | Yes (1)  No (0) | Please press on one box only | If A1=1 STOP the interview and ask to complete the interview with the mother  If A1=0 go to A1b |
| A1b | What is your relationship to the baby’s biological mother? | I am her husband (1)  I am her mother (2)  I am her father (3)  I am her mother in law (4)  I am her father in law (5)  I am her relative (e.g. aunt, sister, cousin) (6)  No relationship, I adopted her child (7)  Other (8) | Please press on one box only | Go to A2a |
| A2a | Is your baby a twin? | Yes (1)  No (0) | Please press on one box only | Go to A3a |
| A2b | Was your baby a twin? | Yes (1)  No (0) | Please press on one box only | Go to A3b |
| A3a | Is your baby a boy or a girl? | A girl (1)  A boy (0) | Please press on one box only | Go to A4 |
| A3b | Was your baby a boy or a girl? | A girl (1)  A boy (0) | Please press on one box only | Go to A5a |
| A4 | How many months old is your baby? | Number pad | Please use the number pad | If Q4=1 go to A6  If Q4=0 go to A70a  Survey date - Q1= A4 |
| Intro A5a | You mentioned that your baby passed away. |  |  |  |
| A5a | I know it may be difficult to talk about this, but can you please tell me when did your baby pass away? (dd/mm/yyyy) | Number pad | Please use the number pad | If A5a=Q1 go to A5b  If A5a>Q1 go to A6  If A5a<Q1 ERROR |
| A5b | You mentioned that your baby died the day he was born. Was your baby born dead? | Yes (1)  No (0) | Please press on one box only | If A5b=1 go to A5c  If A5b=0 go to A6 |
| A5c | How many months was the pregnancy when the baby was born? | Number pad  Don’t remember (8) | Please use the number pad | Go to A5d |
| A5d | Did the pregnancy end earlier than expected? | Yes (1)  No (0)  Don’t remember (8) | Please press on one box only | If A5d=1 go to A5e  If A5d=0,8 go to A6 |
| A5e | How many weeks before the expected date of delivery did the pregnancy end? | Number pad  Don’t remember (8) | Please use the number pad | Go to A6 |
| Intro A6 | We realize that once you got pregnant, you probably looked forward to having the baby. However, think back to the time before you got pregnant with your baby born on [DATE]. |  |  |  |
| A6 | In terms of the timing of the pregnancy with your baby, do you feel like you: (Read out responses) | Wanted the pregnancy at that time (1)  Wanted the pregnancy sooner (2)  Wanted the pregnancy later (3)  Was not planning on getting pregnant at all (4) | Please press on one box only | Go to A7 |
| A7 | Just before you found out you were pregnant with your baby, were you using any method to prevent or avoid pregnancy? | Yes (1)  No (0) | Please press on one box only | Go to A8 |
| A8 | When you were pregnant with your baby, did you seek antenatal care? | Yes (1)  No (0) | Please press on one box only | If A8=1 go to A9  If A8=0 go to A15b |
| A9 | Do you have your maternity record (from the hospital)? | Yes (1)  No (0) | Please press on one box only | If A9=1 go to A10  If A9=0 go to A11 |
| A10 | May I see your maternity record? (If reluctant response, ***encourage to view the booklet***) | Yes (1)  No (0)  I cannot find the booklet at the moment (2) | Please press on one box only | Go to A11 |
| A11 | Did you receive antenatal care for your baby in this locality? (the locality we are currently in) | Yes (1)  No (0) | Please press on one box only | Go to A12 |
| A12 | Where did you first receive antenatal care for your baby? | Home (1)  Government hospital (2)  Government health center (3)  Government health post (4)  Private sector clinic (5)  Mission hospital (6)  Mission health center (7)  Mission health post (8)  Other (9) | Please press on one box only | Go to A13 |
| A13 | Did you ever receive antenatal care for your baby in a health care facility? | Yes (1)  No (0) | Please press on one box only | Go to A14 |
| A14 | ***To the survey assistant:*** Please note the source of information; check the medical record if available | Only recall (1)  Only medical record/health card (2)  Medical record and recall (3)  Other (4) | Please press on one box only | If A13=1 go to A15a  If A13=0 go to A15b |
| A15a | Where did you receive antenatal care during the pregnancy with your baby? (name the clinic) | List of clinics | Please press on one box only | Go to A15c |
| A15b | Why did you not receive antenatal care for your baby at a health care facility? | Clinic is too far away (0/1)  Registry fees were too expensive (0/1)  My husband/partner would not let me (0/1)  Could not take time away from home/work (0/1)  There is no benefit to my baby for going to the clinic during pregnancy (0/1)  Wait times at the clinic are too long (0/1)  Clinic staff are unfriendly (0/1)  Other (0/1) | Please press ALL statements that apply | If A8=1 go to A18  IF A8=0 go to A27 |
| A15c | How much did you pay for round trip transportation to the facility? (in US dollars or rand) | Number pad  I did not have to pay (0) | Please use the number pad | Go to A16 |
| A16 | How long did it take you to get to the antenatal care facility? (one way in minutes) | Number pad | Please use the number pad | Go to A17 |
| A17 | How much did you have to pay for an antenatal appointment? (in US dollars or rand) | Number pad  I did not have to pay (0) | Please use the number pad | Go to A18 |
| A18 | How many months pregnant were you when you **first** received antenatal care? | Number pad | Please use the number pad | Go to A19 |
| A19 | ***To the survey assistant:*** Please note the source of information; check the medical record if available | Only recall (1)  Only medical record/health card (2)  Medical record and recall (3)  Other (4) | Please press on one box only | Go to A20 |
| A20 | How many antenatal visits did you attend during your pregnancy with this baby? | Number pad | Please use the number pad | Go to A21 |
| A21 | ***To the survey assistant:*** Please note the source of information; check the medical record if available | Only recall (1)  Only medical record/health card (2)  Medical record and recall (3)  Other (4) | Please press on one box only | Go to A22 |
| A22 | Were you given a pill to take at the onset of your labor? | Yes (1)  No (0) | Please press on one box only | If A22=1 go to A23  If A22=0 go to A24 |
| A23 | Why were you given this pill? (*Do not read the options aloud*) | To prevent malaria (1)  To cure a specific illness (2)  To prevent transmission of HIV (3)  Other (4)  Don’t know (99) | Please press on one box only | If Q3=0 and A5b=1 go to A27  If Q3=1 or (Q3=0 and A5b=0) go to A24 |
| A24 | Did your baby receive any syrup medicine in his/her mouth very soon after delivery? | Yes (1)  No (0) | Please press on one box only | Go to A25 |
| A25 | After delivery, were you given any pills to take home to give to your baby daily? | Yes (1)  No (0) | Please press on one box only | Go to A26 |
| A26 | Were you given any pills to take daily while you breastfed your baby? | Yes (1)  No (0) | Please press on one box only | Go to A27 |
| A27 | Prior to your pregnancy with your baby, were you ever tested for HIV? | Yes (1)  No (0) | Please press on one box only | If A27=1 go to A28  If A27=0 go to A30 |
| A28 | For any of these HIV tests prior to your pregnancy with your baby, were the results ever positive? | Yes (1)  No (0)  Don’t want to tell (2)  Don’t know (99) | Please press on one box only | If A28=1 go to A29  If A28=0,2,99 go to A30 |
| A29 | In what month and year did you **first** receive a positive test result? (mm/yyyy) | Number pad | Please use the number pad | Go to A30 |
| Intro A30 | Now I would like to ask you a few specific questions about any medical tests you may have had while pregnant with your baby born on [DATE], and any related medication they may have given you. |  |  |  |
| A30 | During your pregnancy with this baby, were you tested for HIV? | Yes (1)  No (0)  Don’t want to tell (2)  Don’t know (99) | Please press on one box only | Go to A31a |
| A31a | ***To the survey assistant:*** Please note the source of information; check the medical record if available | Only recall (1)  Only medical record/health card (2)  Medical record and recall (3)  Other (4) | Please press on one box only | If A30=1 go to A32  If A30=0 go to A31b  If A30=2,99 go to C1 |
| A31b | Why did you not test for HIV? | I was not offered an HIV test (0/1)  I am not at risk for HIV (0/1)  I was scared to find out the result (0/1)  My husband would not let me (0/1)  The test was too expensive (0/1)  Other (0/1) | Please press ALL statements that apply | Go to C1 |
| A32 | In what month and year were you **first** tested for HIV? (mm/yyyy) | Number pad | Please use the number pad | Go to A33 |
| A33 | ***To the survey assistant:*** Please note the source of information; check the medical record if available | Only recall (1)  Only medical record/health card (2)  Medical record and recall (3)  Other (4) | Please press on one box only | Go to A34 |
| A34 | How much did you pay for the test **itself**? (in US dollars or rand) | Number pad  I did not have to pay (0) | Please use the number pad | Go to A35 |
| A35 | Did you have to make a trip to the clinic just to get the results of your HIV test? | Yes (1)  No (0) | Please press on one box only | If A35=1 go to A36  If A35=0 go to A38 |
| A36 | How much did you pay for the round trip transportation to the clinic? (in US dollars or rand) | Number pad  I did not have to pay (0) | Please use the number pad | Go to A37a |
| A37a | How long did it take to go to the clinic for your HIV test result? (one way in minutes) | Number pad | Please use the number pad | Go to A37b |
| A37b | Did you receive the results of the HIV test during the pregnancy with your baby? | Yes (1)  No (0) | Please press on one box only | If A37b=0 go to A37c  If A37b=1 go to A38 |
| A37c | Why did you not get the result of your HIV test during your pregnancy with your baby? | Did not want to find out the results (0/1)  The clinic was to far away (0/1)  Could not take time away from home/work (0/1)  Wait times at the clinic are too long (0/1)  Clinic staff are unfriendly (0/1)  My husband would not let me (0/1)  I moved to a different locality (0/1)  I forgot (0/1)  Other (0/1) | Please press ALL statements that apply | Go to A43 |
| A38 | What was the result of your **first** HIV test when you were pregnant with your baby? | HIV Positive (1)  HIV Negative (0)  Don’t want to tell (2)  Don’t know (99) | Please press on one box only | Go to A39 |
| A39 | ***To the survey assistant:*** Please note the source of information; check the medical record if available | Only recall (1)  Only medical record/health card (2)  Medical record and recall (3)  Other (4) | Please press on one box only | If A38=1,0,2 go to A40  If A38=99 go to A43 |
| A40 | When did you find out the result of your test? | Told that same day (visit) by clinic staff (1)  Had to return at a later date for result (2)  Phone call (3)  Other (4) | Please press on one box only | Go to A41 |
| A41 | Did you tell anyone the result of your HIV test? | Yes (1)  No (0) | Please press on one box only | If A41=1 go to A42  If A41=0 go to A43 |
| A42 | Who did you tell? | Partner/ husband (0/1)  Children (0/1)  Other family member (0/1)  Pastor (0/1)  Friend(s) (0/1)  Other (0/1) | Please press ALL statements that apply | Go to A43 |
| A43 | During this pregnancy, did you give a blood sample to have a CD4 test? | Yes (1)  No (0)  Don’t know (99) | Please press on one box only | If A43=1 go to A44  If A43=0,99 go to A47 |
| A44 | How much did you have to pay for the CD4 test? (in US dollars or rand) | Number pad  I did not have to pay (0) | Please use the number pad | Go to A45 |
| A45 | Did you get the result of the CD4 test? | Yes (1)  No (0) | Please press on one box only | If A45=1 go to A46b  If A45=0 go to A46a  If A45=DK, REF go to A47 |
| A46a | Why did you not get the result of the CD4 test? | Did not want to find out the results (0/1)  The clinic was to far away (0/1)  Could not take time away from home/work (0/1)  Wait times at the clinic are too long (0/1)  Clinic staff are unfriendly (0/1)  My husband would not let me (0/1)  I moved to a different locality (0/1)  I forgot (0/1)  Other (0/1) | Please press ALL statements that apply | Go to A47 |
| A46b | What was the result of the CD4 test? | Number pad  Don’t know/ don’t remember (999) | Please use the number pad | Go to A46c |
| A46c | Did you have to make a trip to the clinic just to get the results of your CD4 test? | Yes (1)  No (0) | Please press on one box only | Go to A46d |
| A46d | From the day you gave the blood sample for the CD4 test, how many days did you have to wait to receive the results of the test? | Number pad  Don’t remember (999) | Please use the number pad | Go to A47 |
| A47 | During this pregnancy, were you **given** any drugs so that you would not transmit HIV to your baby? [show pictures of Nevirapine, AZT and HAART] | Yes (1)  No, I was not given such drugs (0)  Don’t remember (2) | Please press on one box only | Go to A48 |
| A48 | ***To the survey assistant:*** Please note the source of information; check the medical record if available | Only recall (1)  Only medical record/health card (2)  Medical record and recall (3)  Other (4) | Please press on one box only | If A47=1 go to A49  If A47=0,2 go to A68 |
| A49 | What medications were you **given**? [show pictures of Nevirapine and AZT] | Nevirapine only (1)  AZT only (2)  Nevirapine and AZT (3)  HAART (4)  Don’t remember (5)  Other (6) | Please press on one box only | Go to A50 |
| A50 | ***To the survey assistant:*** Please note the source of information; check the medical record if available | Only recall (1)  Only medical record/health card (2)  Medical record and recall (3)  Other (4) | Please press on one box only | If A49=1,3 go to A51  If A49=2 go to A58  If A49=4 go to A64  If A49=5,6 go to A68 |
| A51 | Some mothers are told to take a single tablet of Navirapene around the time of their labour. During the pregnancy with your baby, did you take the Nevirapene tablet? | Yes (1)  No (0)  Don’t remember (2) | Please press on one box only | If A51=0 go to A52a  If A51=1 go to A52b  If A51=DK, REF go to A58 Marker |
| A52a | Why did you not take the Nevirapine tablet? | I forgot to take it (1)  I lost the tablet (2)  I did not have access to the tablet when I went into labor (3)  I did not want to take it (4)  Other (5) | Please press on one box only | Go to A58 Marker |
| A52b | When did you take the Nevirapine tablet? | When I went into labor (1)  After I delivered (2)  1-7 days before delivery (3)  Don’t remember (4) | Please press on one box only | Go to A53 |
| A53 | How many times did you take the Nevirapine tablet? | One time (1)  Two times (2)  More than 2 times (3)  Don’t remember (4) | Please press on one box only | Go to A54 |
| A54 | How much did you pay for the Nevirapine tablet? (in US dollars or rand) | Number pad  I did not have to pay (0) | Please use the number pad | Go to A55 |
| A55 | Did you have to make a trip to the clinic just to get the Nevirapine tablet? | Yes (1)  No (0) | Please press on one box only | If A55=1 go to A56  If A55=0 go to A58 Marker |
| A56 | How much did you pay for the round-trip transportation to get the Nevirapine tablet? | Number pad  I did not have to pay (0) | Please use the number pad | Go to A57 |
| A57 | How long did it take to go get the Nevirapine tablet? (one way in minutes) | Number pad | Please use the number pad | If A49=3 go to A58  If A49=1 go to A68 |
| Intro A58 | You mentioned that during the pregnancy with your baby you were given AZT. |  |  |  |
| A58 | Did you take the AZT tables? | Yes (1)  No (0)  Don’t remember (2) | Please press on one box only | If A58=0 go to A59a  If A58=1 go to A59b  If A58=0,2 go to A61 |
| A59a | Why did you not take the AZT tablets? | I forgot to take them (1)  I lost the tablets (2)  I gave them to someone else (3)  I did not want to take them (4)  Other (5) | Please press on one box only | Go to A61 |
| A59b | How many AZT tablets did you take? | All of the tablets given to me (1)  Some of the tablets given to me (2)  None of the tablets given to me (3)  Don’t remember (4) | Please press on one box only | Go to A60 |
| A60 | When did you start taking the AZT tablets during this pregnancy? | 2 months pregnant (1)  3 months pregnant (2)  4 months pregnant (3)  5 months pregnant (4)  6 months pregnant (5)  7 months pregnant (6)  8 months pregnant (7)  9 months pregnant (8)  10 months pregnant (9) | Please use the number pad | Go to A61 |
| A61 | How much did you have to pay for the AZT tablets? | Number pad  I did not have to pay (0) | Please use the number pad | Go to A62 |
| A62 | How much did the round-trip transportation to get AZT tablets cost? (in US dollars or rand) | Number pad  I did not have to pay (0) | Please use the number pad | Go to A63 |
| A63 | How long did it take you to go get AZT tablets? (one way in minutes) | Number pad | Please use the number pad | Go to A68 |
| Intro A64 | You mentioned that during the pregnancy with your baby you were on HAART medication (HAART is when the mother is on three ARV drugs at one time). |  |  |  |
| A64 | When did you start taking HAART? | Before pregnancy (1)  During pregnancy (2)  After delivery (3)  Never took HAART (4)  Don’t remember (5) | Please press on one box only | If A64=1,2,3 go to A65a  If A64=4,5 go to A68 |
| A65a | How many HAART tablets did you take? | All of the tablets given to me (1)  Some of the tablets given to me (2)  None of the tablets given to me (3)  Don’t remember (4) | Please press on one box only | If A64=2 go to A65b  If A64=1,3 go to A65c |
| A65b | When did you start taking the HAART tablets during this pregnancy? | 2 months pregnant (1)  3 months pregnant (2)  4 months pregnant (3)  5 months pregnant (4)  6 months pregnant (5)  7 months pregnant (6)  8 months pregnant (7)  9 months pregnant (8)  10 months pregnant (9) | Please press on one box only | Go to A65c |
| A65c | How much did you have to pay for one month supply of HAART? (in US dollars or rand) | Number pad  I did not have to pay (0) | Please use the number pad | Go to A66 |
| A66 | How much did you pay per month for transportation to go get a new refill of HAART? (one way in US dollars or rand) | Number pad  I did not have to pay (0) | Please use the number pad | Go to A67 |
| A67 | How long did it take you to go get a new refill of HAART? (one way in minutes) | Number pad | Please use the number pad | Go to A68 |
| A68 | ***To the survey assistant:*** Please note the **last** maternal HIV antibody test (if reported on ANC card) | Positive (1)  Negative (0)  Indeterminate (2)  Not done (3)  Nothing written on card (4) | Please press on one box only | If Q3=0 and A5b=1 go to C1  If Q3=1 or (Q3=0 and A5b=0) go to A69a |
| A69a | Do you have your baby’s health card? | Yes (1)  No (0) | Please press on one box only | If A69a=1 go to A69b  If A69a=0 go to C1 |
| A69b | May I see the baby’s health card? (If reluctant response, ***encourage to view the booklet***) | Yes (1)  No (0)  I cannot find the booklet at the moment (2) | Please press on one box only | If Q69b=1 go to A69c  If Q69b=0,2 go to C1 |
| Intro A69c | ***To the survey assistant:*** Fill in the following information from the booklet (do not ask the baby’s mother). |  |  |  |
| A69c | The baby was given NVP or other ARVs at birth. | Yes (1)  No (0)  Nothing written on card (9) | Please press on one box only | Go to A69d |
| A69d | The baby was given Cotrimoxazole. | Yes (1)  No (0)  Nothing written on card (9) | Please press on one box only | Go to A69e |
| A69e | The baby was tested for HIV. | Yes (1)  No (0)  Nothing written on card (9) | Please press on one box only | If A69e=1 go to A69f  If A69e=0,9 go to C1 |
| A69f | The baby’s HIV test result. | Positive (1)  Negative (0)  Indeterminate (2)  Nothing written on card (9) | Please press on one box only | Go to C1 |
| A70a | Do you have the maternity record of your baby’s biological mother (from the hospital)? | Yes (1)  No (0) | Please press on one box only | If A70a=1 go to A70b  If A70a=0 go to A79 |
| A70b | May I see this maternity record? (If reluctant response, ***encourage to view the booklet***) | Yes (1)  No (0)  I cannot find the booklet at the moment (2) | Please press on one box only | If Q70b=1 go to A70c  If Q70b=0 go to A79 |
| Intro A70c | ***To the survey assistant:*** Fill in the following information from the booklet (do not ask the baby’s caregiver). |  |  |  |
| A70c | The baby’s biological mother received antenatal care in a health care facility. | Yes (1)  No (0)  Nothing written on card (9) | Please press on one box only | If A70=1,9 go to A71  If A70=0 go to A79 |
| A71 | The number of months in her pregnancy at which the biological mother first received antenatal care. | Number pad  Not mentioned (999) | Please use the number pad | Go to A72 |
| A72 | Number of antenatal visits attended during the pregnancy. | Number pad  Nothing written on card (999) | Please use the number pad | Go to A73 |
| A73 | The biological mother was tested for HIV during her pregnancy. | Yes (1)  No (0)  Nothing written on card (9) | Please press on one box only | If A73=1 go to A74  If A73=9 go to A76  If A73=0 go to A79 |
| A74 | Month and year when the biological mother **first** tested for HIV. (mm/yyyy) | Number pad  Nothing written on card (999) | Please use the number pad | Go to A75 |
| A75 | The result of the biological mother’s first HIV test during her pregnancy. | HIV Positive (1)  HIV Negative (0)  Indeterminate (2)  Nothing written on card (9) | Please press on one box only | If A75=1,9 go to A76  If A75=0,2 go to A79 |
| A76 | The biological mother was given drugs to prevent HIV transmission to her baby. | Yes (1)  No (0)  Nothing written on card (9) | Please press on one box only | If A76=1 go to A77  If A76=0,9 go to A79 |
| A77 | The biological mother was given the following medication: | Nevirapine only (1)  AZT only (2)  Nevirapine and AZT (3)  HAART (4) | Please press on one box only | Go to A78 |
| A78 | The result of the last maternal HIV antibody test was: | Positive (1)  Negative (0)  Indeterminate (2)  Nothing written on card (9) | Please press on one box only | Go to A79 |
| A79 | Do you have your baby’s health card? | Yes (1)  No (0) | Please press on one box only | If A79=1 go to A80  If A79=0 go to A85 |
| A80 | May I see the baby’s health card? (If reluctant response, ***encourage to view the booklet***) | Yes (1)  No (0)  I cannot find the booklet at the moment (2) | Please press on one box only | If Q80=1 go to A81  If Q80=0,2 go to A85 |
| Intro A81 | ***To the survey assistant:*** Fill in the following information from the booklet (do not ask the baby’s caregiver). |  |  |  |
| A81 | The baby was given NVP or other ARVs at birth. | Yes (1)  No (0)  Nothing written on card (9) | Please press on one box only | Go to A82 |
| A82 | The baby was given Cotrimoxazole. | Yes (1)  No (0)  Nothing written on card (9) | Please press on one box only | Go to A83 |
| A83 | The baby was tested for HIV. | Yes (1)  No (0)  Nothing written on card (9) | Please press on one box only | If A83=1 go to A84  If A83=0,9 go to A85 |
| A84 | The baby’s HIV test result. | Positive (1)  Negative (0)  Indeterminate (2)  Nothing written on card (9) | Please press on one box only | Go to A85 |
| A85 | As far as you know, has your baby’s biological mother ever tested for HIV? | Yes (1)  No (0)  Don’t know (2) | Please press on one box only | If A85=1 go to A86  If A85=0,2 go to A87 |
| A86 | What was the result of the HIV test? | Positive (1)  Negative (0)  Don’t know (2) | Please press on one box only | Go to A87 |
| A87 | As far as you know, has your baby ever been tested for HIV? | Yes (1)  No (0)  Don’t know (2) | Please press on one box only | If A87=1 go to A88  If A87=0,2 go to C25 |
| A88 | What was the result of the HIV test? | Positive (1)  Negative (0)  Don’t know (2) | Please press on one box only | Go to C25 |
|  | **Section C. Childbirth** |  |  |  |
| Intro C1 | Now I would like to ask you about the birth of your child born on [DATE]. |  |  |  |
| C1 | Did you deliver your baby in this locality? (the locality we are currently in) | Yes (1)  No (0) | Please press on one box only | Go to C2a |
| C2a | Where did you give birth to your baby? | Your home (1)  Other home (2)  Hospital (3)  Health center (4)  Clinic (5)  Other (6) | Please press on one box only | If C2a=3,4,5,6 go to C2b  If C2a=1,2 go to C5  If C2a=DK, REF go to C2c |
| C2b | Where did you deliver your baby? (name the health facility) | List of clinic localities | Please press on one box only | Go to C2c |
| C2c | Were you offered an HIV test during your delivery? | Yes (1)  No (0) | Please press on one box only | If C2c=1 go to C2d  If C2c=0 go to C3 |
| C2d | Did you accept the HIV test during delivery? | Yes (1)  No (0) | Please press on one box only | If C2d =1 go to C2f  If C2d=0 go to C2e |
| C2e | Why did you not accept the HIV test during delivery? | I tested earlier in my pregnancy and was HIV negative (1)  I knew I was HIV positive (2)  I did not want to know my HIV status (3)  I am not at risk for HIV (4)  My husband would not let me (5)  Other (6) | Please press on one box only | Go to C3 |
| C2f | Did you receive the result of the HIV test during delivery? | Yes (1)  No (0) | Please press on one box only | If C2f =1 go to C2g  If C2f=0 go to C3 |
| C2g | What was the result of the HIV test you received during delivery? | Positive (1)  Negative (0)  Don’t know (2) | Please press on one box only | Go to C3 |
| C3 | How much did you pay for round-trip transportation to the place where you delivered your baby? | Number pad  I did not have to pay (0) | Please use the number pad | Go to C4 |
| C4 | How long did it take you to get to the place where you delivered your baby? (one way in minutes) | Number pad | Please use the number pad | Go to C5 |
| C5 | Who made the final decision about where to deliver your baby? | You (1)  The baby’s father (2)  Your mother (3)  Your mother in law (4)  Other (5) | Please press on one box only | If C2a=1,2 and (Q3=1 or (Q3=0 and A5b=0)) go to C6  If C2a=1,2 and (Q3=0 and A5b=1) go to C11  If C2a=3,4,5,6 go to C10  If C2a=DK, REF go to C11 |
| C6 | Was your baby taken to a health center after delivery within 3 days of birth? | Yes (1)  No (0)  Don’t know (2) | Please press on one box only | If C6=1 go to C7  If C6=0,2 go to C11 |
| C7 | How much did you have to pay for the visit to the health center within 3 days of birth? | Number pad  I did not have to pay (0) | Please use the number pad | Go to C8 |
| C8 | How much did you pay for round-trip transportation to the health center within 3 days of birth? | Number pad  I did not have to pay (0) | Please use the number pad | Go to C9 |
| C9 | How long did it take you to go to the health center within 3 days of birth? (one way in minutes) | Number pad | Please use the number pad | Go to C11 |
| C10 | Was your baby delivered by cesarean section? (During the delivery, did a doctor perform surgery to remove the baby from your uterus? If the baby was delivered naturally through the vagina, then it was not a cesarean section.) | Yes (1)  No (0)  Don’t know (2) | Please press on one box only | Go to C11 |
| C11 | Who assisted with the delivery of your baby? | Health professional: Doctor (0/1)  Health professional: Nurse/ midwife (0/1)  Health professional: unskilled worker at health center (0/1)  Traditional/ trained birth attendant (0/1)  Friend/ neighbor (0/1)  Family member (0/1)  No one (0/1)  Other (0/1)  Don’t know (0/1) | Please press ALL statements that apply | Go to C12 |
| C12 | How much did you have to pay for the delivery of your baby (not including transportation)? | Number pad  I did not have to pay (0) | Please use the number pad | If Q3=0 and A5b=1 go to D1  If Q3=1 or (Q3=0 and A5b=0, DK, REF) go to C13 |
| C13 | Was your baby weighed at birth? | Yes (1)  No (0) | Please press on one box only | If C13=1 go to C14  If C13=0 go to C15a |
| C14 | How much did your baby weigh? (in kilograms) | Number pad | Please use the number pad | Go to C15a |
| C15a | Did you go to a clinic for a postnatal visit? Postnatal visits are when the clinic makes sure the mother is healthy after the delivery of a child. | Yes (1)  No (0) | Please press on one box only | If C15a=1 go to C15c  If C15a=0 go to C15b  If C15a=DK, REF go to C15f |
| C15b | Why did you not go to a clinic for a postnatal visit? | I was not told that I should have this visit (1)  My baby and I felt healthy and did not think I needed to go (2)  The clinic was too far away (3)  The clinic staff are unfriendly (4)  I could not take time away from home/work to go to the clinic (5)  I did not want to pay for a clinic visit (6)  My husband would not let me go (7)  Other (8) | Please press on one box only | Go to C15f |
| C15c | Were you given Cotrimoxazole to take for your own health? | Yes (1)  No (0)  Don’t know (9) | Please press on one box only | If C15c=1 go to C15d  If C15c=0,9 go to C15f |
| C15d | Did you take the Cotrimoxazole for your own health as directed by the health facility, in terms of the number of days and dosage? | Yes (1)  No (0) | Please press on one box only | If C15d=1, DK, REF go to C15f  If C15d=0 go to C15e |
| C15e | Why did you not take Cotrimoxazole as directed by the health facility, in terms of the number of days and dosage? | I did not feel sick (1)  It made the me feel sick when I took it (2)  I forgot to take it (3)  A household member told me not to take it (4)  I gave the pills to someone else (5)  I ran out of the pills too quickly (6)  It was too expensive (7)  Other (8) | Please press on one box only | Go to C15f |
| C15f | Did you go to a clinic at some point after the birth of your baby so that your baby could receive immunizations? | Yes (1)  No (0) | Please press on one box only | If C15f =1, DK, REF go to C15h  If C15f=0 go to C15g |
| C15g | Why did you not go to a clinic to have your baby immunized after 3 months from the date of birth? | I was not told when to bring in my child (1)  I do not believe immunizations will help my child (2)  The clinic was too far away (3)  The clinic staff are unfriendly (4)  I could not find take time away from home/work to go to the clinic (5)  I did not want to pay for a clinic visit/the immunizations (6)  My husband would not let me go (7)  Other (8) | Please press on one box only | Go to C15h |
| C15h | Was the baby ever tested for HIV? | Yes (1)  No (0) | Please press on one box only | If C15h=1 go to C16b  If C15h=0 go to C16a  If C15h=DK, REF go to C20b |
| C16a | Why did you not test your baby for HIV? | I was not offered to have my baby tested for HIV (0/1)  I was scared to find out the result (0/1)  My husband would not let me (0/1)  The test was too expensive (0/1)  I did not go back to a clinic after the baby was born (0/1)  The clinic was too far away (0/1)  The clinic staff are unfriendly (0/1)  I could not find take time away from home/work to go to the clinic (0/1)  Other (0/1) | Please press ALL statements that apply | Go to C20b |
| C16b | In what month and year was the baby **last** tested for HIV? (MM/YYYY) | Number pad | Please use the number pad | Go to C17 |
| C17 | How old was the baby when **last** tested for HIV? (in months) | Number pad | Please use the number pad | Go to C18 |
| C18 | Did you get the results of your baby’s HIV test? | Yes (1)  No (0) | Please press on one box only | If C18=1 go to C19a  If C18=0, DK, REF go to C20a |
| C19a | What was the result of your baby's HIV test? | HIV positive (1)  HIV negative (0)  Don’t want to tell (2)  Don’t know (3) | Please press on one box only | If C19a=1 go to C19b  If C19a=0,2,3 go to C20a |
| C19b | Was your baby put on treatment for HIV infection? Specifically, was your baby put on ART drugs? [show pictures] | Yes (1)  No (0)  Don’t know (9) | Please press on one box only | If C19b=0,DK, REF go to C20a  If C19b=1 go to C19c |
| C19c | Did you give your baby the treatment as directed by the health facility, in terms of the number of days and dosage? | Yes (1)  No (0) | Please press on one box only | If C19c=1, DK, REF go to C20a  If C19c=0 go to C19d |
| C19d | Why did you not give your baby the treatment as directed by the health facility? | Baby would not take it (0/1)  It made the baby sick (0/1)  I forgot (0/1)  A household member told me not to (0/1)  I ran out of the medicine too quickly (0/1)  The treatment was too expensive (0/1)  Other (0/1) | Please press ALL statements that apply | Go to C20a |
| C20a | How much did you have to pay for your baby’s HIV test? (in US dollars or rand) | Number pad  I did not have to pay (0) | Please use the number pad | Go to C20b |
| C20b | Has the baby ever spent the night in a clinic or hospital **after** being discharged from birth facility? | Yes (1)  No (0)  Don’t know/ don’t remember (2) | Please press on one box only | If C20b=1 go to C21  If C20b=0,2 go to C25 |
| C21 | How many times has your baby been hospitalized? | 1 time (1)  2 times (2)  3 times (3)  4 times (4)  5 times or more (5)  Don’t know (6) | Please press on one box only | Go to C22 |
| C22 | How much did you have to pay for the hospitalization? | Number pad  I did not have to pay (0) | Please use the number pad | Go to C23 |
| C23 | How much did you pay for round-trip transportation for one hospitalization? (one way in US dollars or rand) | Number pad  I did not have to pay (0) | Please use the number pad | Go to C24 |
| C24 | How long did it take you to go to the clinic or hospital? (one way in minutes) | Number pad | Please use the number pad | If Q3=1 go to C25  If Q3=0 go to Marker C29 |
| C25 | Did your baby have diarrhea in the past 4 weeks (more than 3 watery stools per day)? | Yes (1)  No (0) | Please press on one box only | Go to C26 |
| C26 | During the past 12 months, how many times did your baby have parasites (like intestinal worms)? | Number pad  Don’t know (999) | Please use the number pad | Go to C27 |
| C27 | During the past 4 weeks, did your baby have any respiratory illness (cold, pneumonia, cough)? | Yes (1)  No (0) | Please press on one box only | Go to C28 |
| C28 | In the past 4 weeks, how many days has your baby been sick? | Number pad | Please use the number pad | If Q4=0 go to F1  If Q4=1 and (A28=1 or A38=1) and C2a=1,2 go to C29  If Q4=1 and (A28=1 or A38=1) and C2a=3,4,5,6 go to C31  If Q4=1 and A28=0,2,99 and A38=0,2,99 OR (C2a=DK, REF) go to Marker F1 |
| Intro C29 | You mentioned that you delivered your baby at home. |  |  |  |
| C29 | Were you given ARVs **for yourself** to take home **after** the birth of your baby? | Yes (1)  No (0)  Don’t know (2) | Please press on one box only | Go to C30 |
| C30 | Were you given ARVs to **administer to your baby** at home? | Yes (1)  No (0)  Don’t know (2) | Please press on one box only | If Q3=1 go to F1  If Q3=0 go to F6 |
| Intro C31 | You mentioned that you delivered your baby at a health facility. |  |  |  |
| C31 | Did your baby receive any Nevirapine syrup in his/her mouth before discharge from the health facility? (Nevirapine for the child is a syrup given to the baby within 3 days of delivery) | Yes (1)  No (0)  Don’t know/ don’t remember (2) | Please press on one box only | Go to C32a |
| C32a | Were you given Nevirapine to give to your baby while you were home? | Yes (1)  No (0)  Don’t know/ don’t remember (2) | Please press on one box only | If C32a=1 go to C32b  If C32a=0,2 go to C33 |
| C32b | Did you give your baby the Nevirapine as instructed by the health facility, in terms of the number of days and dosage? | Yes (1)  No (0) | Please press on one box only | If C32b=0, DK, REF, go to C32c  If C32b=1 go to C32d |
| C32c | Why did you not give your baby the Nevirapine as instructed by the health facility? | Baby would not take it (0/1)  It made the baby sick (0/1)  I forgot (0/1)  A household member told me not to (0/1)  I ran out of the medicine too quickly (0/1)  The treatment was too expensive (0/1)  Other (0/1) | Please press ALL statements that apply | Go to C32d |
| C32d | How much did you pay for the Nevirapine syrup? | Number pad  I did not have to pay (0) | Please use the number pad | Go to C33 |
| C33 | Did your baby receive any AZT syrup in his/her mouth before discharge from the health facility? (AZT for the child is a syrup given with a syringe twice a day) | Yes (1)  No (0)  Don’t know/ don’t remember (2) | Please press on one box only | Go to C34a |
| C34a | Were you given AZT to give to your baby while you were home? | Yes (1)  No (0)  Don’t know/ don’t remember (2) |  | If C34a=1 go to C34b  If C34a=0,2 go to C35 |
| C34b | Did you give your baby the AZT as instructed by the health facility? (number of days and dosage) | Yes (1)  No (0) |  | If C34b=0 go to C34c  If C34b=1, DK, REF go to C34d |
| C34c | Why did you not give your baby the AZT as instructed by the health facility? | Baby would not take it (0/1)  It made the baby sick (0/1)  I forgot (0/1)  A household member told me not to (0/1)  I ran out of the medicine too quickly (0/1)  The treatment was too expensive (0/1)  Other (0/1) | Please press ALL statements that apply | Go to C34d |
| C34d | How much did you pay for the AZT syrup? | Number pad  I did not have to pay (0) | Please use the number pad | Go to C35 |
| C35 | Was the baby given Cotrimoxazole to take every day beginning at 6 weeks even when s/he was not sick? (a small part of a pill taken daily) | Yes (1)  No (0)  Don’t know/ don’t remember (2) | Please press on one box only | If C35=1 go to C36a  If C35=0,2 go to F1 |
| C36a | Did you give your baby the Cotrimoxizole as instructed by the health facility, in terms of the number of days and dosage? | Yes (1)  No (0) | Please press on one box only | If C36a=0 go to C36b  If C36a=1, DK, REF go to C36c |
| C36b | Why did you not give your baby the Cotrimoxizole as instructed by the health facility? | Baby would not take it (0/1)  It made the baby sick (0/1)  I forgot (0/1)  A household member told me not to (0/1)  I ran out of the medicine too quickly (0/1)  The treatment was too expensive (0/1)  Other (0/1) | Please press ALL statements that apply | Go to C36c |
| C36c | How much did you pay for a week’s worth of Cotrimoxazole? | Number pad  I did not have to pay (0) | Please use the number pad | If Q3=1 go to F1  If Q3=0 go to F6 |
|  | **Section F. Infant feeding practices** |  |  |  |
| Intro F1 | Now I will ask you some questions about what you feed your baby born on [DATE]. |  |  |  |
| F1 | Do **you personally** normally feed and change your baby during the day? | Yes (1)  No (0) | Please press on one box only | If F1=1 go to F2a  If F1=0 go to F6 |
| Intro F2a | Now I am going to ask you if you gave your baby the following items **at all in the last week** ending this morning. |  |  |  |
| F2a | Breast milk | Yes (1)  No (0) | Please press on one box only | Go to F2b |
| F2b | Infant formula | Yes (1)  No (0) | Please press on one box only | Go to F2c |
| F2c | Fresh cow's milk, tea with milk, or weak porridge | Yes (1)  No (0) | Please press on one box only | Go to F2d |
| F2d | Water only, water with sugar/glucose, fruit juice, tea without milk, rice water | Yes (1)  No (0) | Please press on one box only | Go to F2e |
| F2e | Solid food (e.g. sadza, yogurt, cheese, cereals, porridge, bread, fermented porridge, fruits/vegetables, meat/fish/chicken, eggs) | Yes (1)  No (0) | Please press on one box only | Go to F2f |
| F2f | Traditional herbs and/or traditional medicines, non-prescribed over the counter medicines | Yes (1)  No (0) | Please press on one box only | Go to F2g |
| F2g | Prescribed Medicine | Yes (1)  No (0) | Please press on one box only | Go to F2h |
| F2h | Other | Yes (1)  No (0) | Please press on one box only | If F2b=1 go to F3  If F2b=0 go to F6 |
| Intro F3 | You mentioned that in the last week you gave your baby infant formula. |  |  |  |
| F3 | Where do you get infant formula? | Buy it (1)  Receive it from clinic (2)  Other (3)  Don’t know (99) | Please press on one box only | Go to F4 |
| F4 | Did you ever run out of formula? | Yes (1)  No (0) | Please press on one box only | If F4=1 go to F5  If F4=0 go to F6 |
| F5 | What did you feed your baby when you did not have formula? | Breast milk (0/1)  Glucose water (0/1)  Water (0/1)  Other (0/1) | Please press ALL statements that apply | Go to F6 |
| F6 | Was your baby ever breastfed? | Yes (1)  No (0)  Don’t know (99) | Please press on one box only | If F6=1 and Q4=1 go to F7a  If F6=1 and Q4=0 go to F8  If F6=0,99 and Q4=1 go to F14  If F6=0,99 and Q4=0 go to D1 |
| F7a | May I request you to confirm that YOU breastfed your baby. | Yes (1)  No (0) | Please press on one box only | Go to F7b |
| F7b | Did anyone else **ever** breastfed your baby? | Yes (1)  No (0)  Don’t know (99) | Please press on one box only | If Q3=1 go to F9  If Q3=0 go to F10 |
| F8 | Who are all the people who **ever** breastfed your baby? | Biological mother (0/1)  Yourself (0/1)  Someone else (0/1) | Please press ALL statements that apply | Go to F9 |
| F9 | Is your baby still breastfeeding? | Yes (1)  No (0) | Please press on one box only | If Q4=1 go to F10  If Q4=0 go to D1 |
| F10 | How long after birth did you first put your baby on the breast? (read out responses) | Immediately (1)  Less than one hour (2)  Less than 24 hours (3)  More than 24 hours later (4) | Please press on one box only | Go to F11 |
| F11 | Did you ever exclusively breastfeed your baby? (did you feed the baby only breast milk and nothing else for any period of time) | Yes (1)  No (0) | Please press on one box only | If F11=1 go to F12  If F11=0 go to F13 |
| F12 | For how long was your baby exclusively breastfed? (in months) | Number pad | Please use the number pad | Go to F13 |
| F13 | For how many months total have you breastfed your baby? (exclusively and combined with other products ) | Number pad | Please use the number pad | Go to F14 |
| F14 | Did anyone at the health facility instruct you on how to feed your baby after you were discharged or when you came for post natal care? | Yes (1)  No, no one instructed me (0)  No, I never went to a health facility with my baby (2) | Please press on one box only | If A28=1 or A38=1 go to F15  If A28=0,DK,REF and A38=0, DK,REF go to D1 |
| Intro F15 | Now I will ask you about the HIV treatment for you and your baby while you were breastfeeding. |  |  |  |
| F15 | Were you on HIV treatment when you started breastfeeding your baby? | Yes (1)  No (0) | Please press on one box only | If F15=1 go to F16  If F15=0 go to F17 |
| F16 | Did you remain on HIV treatment for the **entire** time that you were breastfeeding your baby? | Yes (1)  No (0)  Don’t remember (2) | Please press on one box only | If F16=1 go to F19  If F16=0 go to F22 |
| F17 | Were you put on treatment at some point AFTER delivery and AFTER initiation of breastfeeding? | Yes (1)  No (0)  Don’t remember (2) | Please press on one box only | If F17=1 go to F18  If F17=0,2 go to F22 |
| F18 | Why were you put on treatment at some point AFTER delivery and AFTER initiation of breastfeeding? | New HIV test result was positive (1)  Decided you wanted to for baby sake (2)  Got money for medicine (3)  Clinic got new medicine (4)  Other (5) | Please press on one box only | Go to F19 |
| F19 | How much did you pay for one month worth of this treatment? | Number pad  I did not have to pay (0) | Please use the number pad | Go to F20 |
| F20 | How many months were you on this treatment? | Number pad | Please use the number pad | Go to F21 |
| F21 | How long did it take you to go get a refill of this treatment? (one way in minutes) | Number pad | Please use the number pad | Go to F22 |
| F22 | Was your baby put on HIV medication at some point AFTER delivery and AFTER initiation of breastfeeding? | Yes (1)  No (0)  Don’t remember (2) | Please press on one box only | If F22=1 go to F23  If F22=0,2 go to D1 |
| F23 | Why was your baby put on HIV medication at some point AFTER delivery and AFTER initiation of breastfeeding? | New HIV test result was positive (1)  Decided you wanted to for baby sake (2)  Got money for medicine (3)  Clinic got new medicine (4)  Clinic said the baby needed it (5)  Other (6) | Please press on one box only | Go to F24 |
| F24 | How much did you pay for one month of HIV medication for your baby? | Number pad  I did not have to pay (0) | Please use the number pad | Go to D1 |
|  | **Section D. Demographic characteristics** |  |  |  |
| Intro D1 | Now we would like to ask you a few questions about yourself. |  |  |  |
| D1 | How long have you lived in this place? | I have always lived here (0)  I have lived here continuously for some years but not my whole life (1)  I have lived here on and off for some years but not my whole life (2)  I have lived here for less than 1 year (3)  I do not live here, I am just visiting (4) | Please press on one box only | Go to D2 |
| D2 | What is the highest level of education that you have completed? | No schooling/ education (0)  Primary school (1)  Form 1 (2)  Form 2 (ZJC) (3)  Form 3 (4)  Form 4 (O level) (5)  Form 5 (6)  Form 6 (A level) (7)  College, certificate, degree (8)  Don’t know (99) | Please press on one box only | Go to D3 |
| D3 | What tribe or ethnic group do you belong to/identify with? | Shona (1)  Ndebele (2)  Kalanga (3)  Other (4) | Please press on one box only | Go to D4 |
| D4 | What is your religion? | Roman Catholic (1)  Anglican (2)  Lutheran (3)  Methodist (4)  Baptist (5)  Presbyterian (6)  Apostolic (Manrange/Masowe) (7)  Pentecostal (8)  Moslem (9)  African traditional religion (10)  Other (11)  No religion (12) | Please press on one box only | If Q4=1 go to D5  If Q4=0 go to H1 |
| D5 | How many times have you given birth in your life, including stillbirths? | Number pad | Please use the number pad | Go to D6 |
| D6 | What best describes your current marital situation? | Currently married and living with husband (1)  Currently married but living separately for work or family reasons (2)  Divorced or separated (3)  Widowed (last husband died) (4)  Never been married (5) | Please press on one box only | If D6=1 go to D8  If D6=2 go to D7  If D6=3,4,5 go to D9  If D6=DK, REF go to H1 |
| D7 | Does your husband come home regularly to stay with you, for example on weekends? | Yes (1)  No (0) | Please press on one box only | Go to D8 |
| D8 | How many wives does your husband have (including yourself)? | Number pad | Please use the number pad | If D6=2 & D7=0, DK, REF go to D9  If (D6=2 & D7=1) or (D6=1) go to D10 |
| D9 | Do you currently have a regular sexual partner? | Yes (1)  No (0) | Please press on one box only | If D9=1 or (D9=0 and D6=2) go to D10  If D9=0,DK and D6=3,4,5 go to H1 |
| D10 | Is your husband/ partner the biological father of your child born on [DATE]? | Yes (1)  No (0) | Please press on one box only | Go to D11 |
| D11 | What is the highest level of education that your husband/ partner has completed? | No schooling/ education (0)  Primary school (1)  Form 1 (2)  Form 2 (ZJC) (3)  Form 3 (4)  Form 4 (O level) (5)  Form 5 (6)  Form 6 (A level) (7)  College, certificate, degree (8)  Don’t know (99) | Please press on one box only | Go to D12 |
| D12 | What tribe or ethnic group does your husband/ partner belong to/ identify with? | Shona (1)  Ndebele (2)  Kalanga (3)  Other (4) | Please press on one box only | Go to D13 |
| D13 | What is your husband/ partner's religion? | Roman Catholic (1)  Anglican (2)  Lutheran (3)  Methodist (4)  Baptist (5)  Presbyterian (6)  Apostolic (Manrange/Masowe) (7)  Pentecostal (8)  Moslem (9)  African traditional religion (10)  Other (11)  No religion (12) | Please press on one box only | Go to H1 |
|  | **Section H. Household characteristics** |  |  |  |
| Intro H1 | We would like to ask you some questions about your household. By household we mean all the people that live together in this house and eat out of the same pot. |  |  |  |
| H1 | How many people normally live in this household? (include yourself, all adults, and all children) | Number pad | Please use the number pad | If Q4=1 go to H2  If Q4=0 go to H4 |
| H2 | Does your mother live with you? | Yes (1)  No (0) | Please press on one box only | Go to H3 |
| H3 | Does your mother-in-law live with you? | Yes (1)  No (0) | Please press on one box only | Go to H4 |
| H4 | During the past 6 months, how many household members had a regular income (earned money on a monthly or weekly basis)? | Number pad | Please use the number pad | Go to H5  H4≤H1 |
| H5 | Think about the building in your homestead that is the most appealing. What material is it built of? | Poles and dagga (1)  Mud bricks (2)  Cement blocks (3)  Stones (4)  Bricks and cement (5)  Blocks and cement (6)  Informal material/corrugated iron/wood (7)  Other (8) | Please press on one box only | Go to H6 |
| H6 | What kind of toilet facility does your household use? | Flush toilet (1)  Our own Blair (2)  Our neighbor’s Blair (3)  Traditional pit latrine (4)  Bucket toilet (5)  The bush/ field (6)  Other (7) | Please press on one box only | Go to H7 |
| H7 | What is the main source of drinking water for members of your household? | Piped water into house (1)  Piped water outside but available within plot (2)  Public tap (3)  Tube well or borehold (4)  Protected well (5)  Unprotected well (6)  Protected spring (7)  Unprotected spring (8)  Rainwater (9)  Tanker truck (10)  Cart with small tank (11)  Surface water (river/lake/pond/stream) (12)  Bottled water (13)  Other (14) | Please press on one box only | Go to H8 |
| H8 | Do you do anything to the water to make it safer to drink? | Yes (1)  No (0)  Don’t know (99) | Please press on one box only | If H8=1 go to H9  If H8=0,99 go to H10 |
| H9 | What do you usually do to make the water safer to drink? | Boil (0/1)  Add bleach/ chlorine (0/1)  Use water filter (0/1)  Solar disinfection (0/1)  Other (0/1)  Don’t know (0/1) | Please press ALL statements that apply | Go to H10  If H9a=1 or H9b=1 or H9c=1 or H9d=1 or H9e=1then H9f=0 |
| H10 | Do you have the following items in your household? | Electricity (0/1)  Refrigerator (0/1)  Stove (electric, gas, or wood) (0/1)  Drinking tap water in the house (0/1)  Livestock (goats, sheep, cattle, donkeys) (0/1)  Bicycle (0/1)  Motorcycle (0/1)  Car/truck in working condition (0/1)  Scotch cart (0/1)  Wheel barrow (0/1)  Phone (cell or landline) (0/1)  Radio (0/1)  Television (0/1) | Please press ALL statements that apply | Go to H11 |
| Intro H11 | Now think about yourself and your household. |  |  |  |
| H11 | In the past four weeks, did you worry that your household would not have enough food? | Yes (1)  No (0) | Please press on one box only | If H11=1 go to H12  If H11=0 go to H13 |
| H12 | How often did this happen? | Rarely (once or twice in the past four  weeks) (1)  Sometimes (three to ten times in the past  four weeks) (2)  Often (more than ten times in the past four  weeks) (3) | Please press on one box only | Go to H13 |
| H13 | In the past four weeks, were you or any household member not able to eat the  kinds of foods you preferred because of a lack of resources? | Yes (1)  No (0) | Please press on one box only | If H13=1 go to H14  If H13=0 go to H15 |
| H14 | How often did this happen? | Rarely (once or twice in the past four  weeks) (1)  Sometimes (three to ten times in the past  four weeks) (2)  Often (more than ten times in the past four  weeks) (3) | Please press on one box only | Go to H15 |
| H15 | Did you or any household member go to sleep at night hungry because there was not enough food? | Yes (1)  No (0) | Please press on one box only | If H15=1 go to H16  If H15=0 go to U1a |
| H16 | How often did this happen? | Rarely (once or twice in the past four  weeks) (1)  Sometimes (three to ten times in the past  four weeks) (2)  Often (more than ten times in the past four  weeks) (3) | Please press on one box only | Go to U1a |
|  | **Section U. Health care utilization** |  |  |  |
| Intro U1a | Now we will ask you some questions about your health related behaviour. When you are sick and want to get medical advice or treatment, how much of a problem is each of the following? |  |  |  |
| U1a | Knowing where to go | Not a problem (1)  Somewhat of a problem (2)  A big problem (3) | Please press on one box only | Go to U1b |
| U1b | Getting permission to go | Not a problem (1)  Somewhat of a problem (2)  A big problem (3) | Please press on one box only | Go to U1c |
| U1c | Getting money needed for treatment | Not a problem (1)  Somewhat of a problem (2)  A big problem (3) | Please press on one box only | Go to U1d |
| U1d | The distance to the health facility | Not a problem (1)  Somewhat of a problem (2)  A big problem (3) | Please press on one box only | Go to U1e |
| U1e | Cost of transportation to the facility | Not a problem (1)  Somewhat of a problem (2)  A big problem (3) | Please press on one box only | Go to U1f |
| U1f | Not wanting to go alone | Not a problem (1)  Somewhat of a problem (2)  A big problem (3) | Please press on one box only | Go to U1g |
| U1g | Concern that there might not be a female health provider | Not a problem (1)  Somewhat of a problem (2)  A big problem (3) | Please press on one box only | Go to U1h |
| U1h | Concern that health providers might be unfriendly or hostile | Not a problem (1)  Somewhat of a problem (2)  A big problem (3) | Please press on one box only | Go to U1j |
| U1j | Concern about the quality of care you would receive there | Not a problem (1)  Somewhat of a problem (2)  A big problem (3) | Please press on one box only | Go to U2 |
| U2 | In the last 12 months, have you visited a health facility for care for yourself or for your children? | Yes (1)  No (0) | Please press on one box only | If Q4=1 go to U3  If Q4=0 go to I1 |
| Intro U3 | Now I will ask you a few questions about your use of family planning methods. |  |  |  |
| U3 | Are you **currently** doing something or using any method to delay or avoid getting pregnant? | Yes (1)  No (0) | Please press on one box only | If U3=1 go to U4  If U3=0 go to U6 |
| U4 | Are you **currently** using any of the following methods? | Female sterilization/hysterectomy (0/1)  Male sterilization (0/1)  Oral contraceptive pills (0/1)  IUD (0/1)  Shot or injection (0/1)  Implant (0/1)  Male condom (0/1)  Female condom (0/1)  Diaphragm (0/1)  Patch (0/1)  Foam/ jelly (0/1)  Lactational Amen. Method (0/1)  Natural family planning/Rhythm (0/1)  Withdrawal (0/1)  Other (0/1) | Please press ALL statements that apply | If U4c=1 or U4e=1 or U4g=1 or U4h=1 or U4j=1 or U4k=1 go to U5  If else go to U6 |
| U5 | How much do you have to pay for contraceptives per month? (in US dollars or rand) | Number pad  I don’t have to pay (0) | Please use the number pad | Go to U6 |
| U6 | Are you currently pregnant? | Yes (1)  No (0)  Not sure (2) | Please press on one box only | Go to I1 |
|  | **Section I. Income and consumption** |  |  |  |
| I1 | Some women take up jobs for which they are paid in cash or kind. Others sell things, have a small business or work on the family farm or in the family business. In the last seven days, have you done any of these things or any other work? | Yes (1)  No (0) | Please press on one box only | If I1=0 go to I2  If I1=1, DK, REF go to I3 |
| I2 | Although you did not work in the last seven days, do you have any job or business from which you were absent for leave, illness, vacation, maternity leave of any other such reasons? | Yes (1)  No (0) | Please press on one box only | If I2=1 go to I3  If I2=0 go to I5 |
| I3 | What is your occupation, that is, what kind of work do you do? | Crop farmer (1)  Animal farmer (2)  Housewife (3)  Trader/merchant/salesperson (4)  Transport worker (5)  Construction worker (6)  Teacher/education professional (7)  Health professional/Traditional birth attendant/Traditional healer (8)  Secretary/Executive secretary (9)  Factory worker (10)  Restaurant/Bar/Hotel Worker (11)  Skilled trades (12)  Preacher/pastor/clerical (13)  Region/district elder (14)  Domestic worker (15)  Civil servant / Government (16)  Other (17) | Please press on one box only | Go to I4 |
| I4 | In the last week you worked, how much money did you earn per week? (in US dollars or rand) | Number pad | Please use the number pad | Go to I5 |
| I5 | Does a woman or a man make most of the important decisions in this household? | Woman (1)  Man (2)  Both (3) | Please press on one box only | Go to I6 |
| I6 | How much has your household spent on food in the **past week**? (in US dollars or rand) | Number pad  Don’t know (999) | Please use the number pad | Go to I7 |
| I7 | How much has your household spent on transport in the **past week**? (in US dollars or rand) | Number pad  Don’t know (999) | Please use the number pad | Go to I8 |
| I8 | How much did you spend on the following items in the **past month**? | Charcoal (Number Pad)  Paraffin (Number Pad)  Candles (Number Pad)  Soap (Number Pad)  Matches (Number Pad)  Toiletries (Number Pad)  Bicycle repairs/ parts (Number Pad)  Batteries (Number Pad)  Clothing (Number Pad)  Kitchen utensils (Number Pad)  Other household items (Number Pad) | Please use the number pad. Enter 0 for ‘My household did not spend money on this item in the past month’. | Go to I9 |
| I9 | How much did your household spend on the following education-related items in the **past year**? | School fees (Number pad)  Uniforms (Number pad)  Books (Number pad)  Stationary (Number pad)  Boarding feeds (Number pad)  Tutoring (Number pad)  Contribution to school building and security guard (Number pad) | Please use the number pad. Enter 0 for ‘My household did not spend money on this item in the past month’. | Go to I10 |
| I10 | How much has your household spend on medical expenses (including medicines) in the **past month**? | Number pad | Please use the number pad | If Q4=0 go to M1  If Q3=0 and A5b<>1 and A69f<>1 and C19a<>1 and (A5-Q1)<29 days go to V1  If Q3=0 and A5b<>1 and A69f<>1 and C19a<>1 and (A5-Q1)>28 days go to W1  If Q3=1 and Q4=1 FINISH |
|  | **Section M. Mother’s verbal autopsy** |  |  |  |
| Intro M1 | Now I will ask you some questions about your baby’s biological mother. |  |  |  |
| M1 | Did your baby’s biological mother pass away? | Yes (1)  No (0) | Please press on one box only | If (A75=1 or A86=1) FINISH  If A75=0,DK and A86=0,DK and M1=1 go to M2  If A75=0 and A86=0 and M1=0 FINISH |
| M2 | Did you live with the biological mother in the period leading to her death? | Yes (1)  No (0) | Please press on one box only | If M2=1 go to M3  If M2=0 FINISH |
| Intro M3 | I know it may be difficult to talk about this, but I will ask you some questions about the events surrounding the death of your baby’s biological mother. |  |  |  |
| M3 | How old was the mother when she died? | Number pad  Don’t know (999) | Please use the number pad | Go to M4 |
| M4 | What was her occupation, that is, what kind of work did she mainly do? | Crop farmer (1)  Animal farmer (2)  Housewife (3)  Trader/merchant/salesperson (4)  Transport worker (5)  Construction worker (6)  Teacher/education professional (7)  Health professional/Traditional birth attendant/Traditional healer (8)  Secretary/Executive secretary (9)  Factory worker (10)  Restaurant/Bar/Hotel Worker (11)  Skilled trader (12)  Preacher/pastor/clerical (13)  Region/district elder (14)  Domestic worker (15)  Civil servant / Government (16)  Other (17)  Don’t know (99) | Please press on one box only | Go to M5 |
| M5 | What is the highest level of education that the mother completed? | No schooling/ education (0)  Primary school (1)  Form 1 (2)  Form 2 (ZJC) (3)  Form 3 (4)  Form 4 (O level) (5)  Form 5 (6)  Form 6 (A level) (7)  College, certificate, degree (8)  Don’t know (99) | Please press on one box only | Go to M6 |
| M6 | What was her marital situation? | Married and living with husband (1)  Married but living separately for work or family reasons (2)  Divorced or separated (3)  Widowed (last husband died) (4)  Never been married (5)  Don’t know (99) | Please press on one box only | Go to M7 |
| Intro M7 | When did she die? |  |  |  |
| M7a | Day (dd) | Number pad  Don’t know (999) | Please use the number pad | Go to M7b |
| M7b | Month (mm) | Number pad  Don’t know (999) | Please use the number pad | Go to M7c |
| M7c | Year (yyyy) | Number pad  Don’t know (999) | Please use the number pad | Go to M8 |
| M8 | Where did she die? | Hospital (1)  Other health facility (2)  Home (3)  Other (4)  Don’t know (9) | Please press on one box only | Go to M9a |
| Intro M9a | I would like to ask you some questions concerning previously known medical conditions the mother had; injuries and accidents that she suffered; and signs and symptoms that she had/showed when she was ill. Some of these questions may not appear to be directly related to her death.  Please bear with me and answer all the questions. They will help us get a clear picture of all possible symptoms that the she had.  Please tell me if the deceased suffered from any of the following illnesses: |  |  |  |
| M9a | High blood pressure? | Yes (1)  No (0)  Don’t know (9) | Please press on one box only | Go to M9b |
| M9b | Diabetes? | Yes (1)  No (0)  Don’t know (9) | Please press on one box only | Go to M9c |
| M9c | Asthma? | Yes (1)  No (0)  Don’t know (9) | Please press on one box only | Go to M9d |
| M9d | Epilepsy? | Yes (1)  No (0)  Don’t know (9) | Please press on one box only | Go to M9e |
| M9e | Malnutrition? | Yes (1)  No (0)  Don’t know (9) | Please press on one box only | Go to M9f |
| M9f | Cancer? | Yes (1)  No (0)  Don’t know (9) | Please press on one box only | Go to M9g |
| M9g | HIV/AIDS? | Yes (1)  No (0)  Don’t know (9) | Please press on one box only | Go to M9h |
| M9h | Tuberculosis? | Yes (1)  No (0)  Don’t know (9) | Please press on one box only | If M9h=1 go to M9j  If M9h=0,9 go to M9k |
| M9j | How long has she been suffering from tuberculosis? (in months) | Number pad  Don’t know (999) | Please use the number pad | Go to M9k |
| M9k | Did s/he suffer from any other medically diagnosed illness? | Yes (1)  No (0)  Don’t know (9) | Please press on one box only | Go to M10 |
| M10 | Did she suffer from any injury or accident that led to her death? | Yes (1)  No (0)  Don’t know (9) | Please press on one box only | If M10=1 go to M11  If M10=0,9 go to M13 |
| M11 | What kind of injury or accident did she suffer? | Road traffic accident (1)  Fall (2)  Downing (3)  Poisoning (4)  Burns (5)  Violence/ assault (6)  Other (7)  Don’t know (9) | Please press on one box only | Go to M12 |
| M12 | Was the injury or accident intentionally inflicted by someone else? | Yes (1)  No (0)  Don’t know (9) | Please press on one box only | Go to M13 |
| M13 | Do you think that she committed suicide? | Yes (1)  No (0)  Don’t know (9) | Please press on one box only | Go to M14 |
| M14 | Did she suffer from any animal/ insect bite that led to her death? | Yes (1)  No (0)  Don’t know (9) | Please press on one box only | If M14=1 go to M15  If M14=0,9 go to M16 |
| M15 | What type of animal or insect? | Dog (1)  Snake (2)  Insect (3)  Other (4)  Don’t know (9) | Please press on one box only | Go to M16 |
| M16 | Did she have an ulcer or swelling in the breast? | Yes (1)  No (0)  Don’t know (9) | Please press on one box only | If M16=1 go to M17  If M16=0,9 go to M18 |
| M17 | For how long did she have an ulcer or swelling in the breast? (in months) | Number pad  Less than 1 month (0)  Don’t know (999) | Please use the number pad | Go to M18 |
| M18 | Did she have excessive vaginal bleeding during menstrual periods? | Yes (1)  No (0)  Don’t know (9) | Please press on one box only | If M18=1 go to M19  If M18=0,9 go to M20 |
| M19 | Within a month, for how many days did s/he have excessive vaginal bleeding during menstrual periods? | Number pad  Don’t know (999) | Please use the number pad | Go to M20 |
| M20 | Did she have vaginal bleeding in between menstrual periods? | Yes (1)  No (0)  Don’t know (9) | Please press on one box only | If M20=1 go to M21  If M20=0,9 go to M22 |
| M21 | For how long did she have vaginal bleeding in between menstrual periods? (in days) | Number pad  Don’t know (999) | Please use the number pad | Go to M22 |
| M22 | Did she have abnormal vaginal discharge? | Yes (1)  No (0)  Don’t know (9) | Please press on one box only | If M22=1 go to M23  If M22=0,9 go to M24 |
| M23 | For how long did she have abnormal vaginal discharge? (in days) | Number pad  Don’t know (999) | Please use the number pad | Go to M24 |
| M24 | Was she pregnant at the time of death? | Yes (1)  No (0)  Don’t know (9) | Please press on one box only | If M24=1 go to M25  If M24=0,9 go to M28 |
| M25 | How long was she pregnant? (in weeks) | Number pad  Don’t know (999) | Please use the number pad | Go to M26a |
| Intro M26a | During the last 3 months of pregnancy, did she suffer from any of the following illnesses: |  |  |  |
| M26a | Vaginal bleeding? | Yes (1)  No (0)  Don’t know (9) | Please press on one box only | Go to M26b |
| M26b | Smelly vaginal discharge? | Yes (1)  No (0)  Don’t know (9) | Please press on one box only | Go to M26c |
| M26c | Puffy face? | Yes (1)  No (0)  Don’t know (9) | Please press on one box only | Go to M26d |
| M26d | Headache? | Yes (1)  No (0)  Don’t know (9) | Please press on one box only | Go to M26e |
| M26e | Blurred vision? | Yes (1)  No (0)  Don’t know (9) | Please press on one box only | Go to M26f |
| M26f | Convulsion (sudden, violent, irregular movement of the body)? | Yes (1)  No (0)  Don’t know (9) | Please press on one box only | Go to M26g |
| M26g | Febrile illness? | Yes (1)  No (0)  Don’t know (9) | Please press on one box only | Go to M26h |
| M26h | Severe abdominal pain that was not labor pain? | Yes (1)  No (0)  Don’t know (9) | Please press on one box only | Go to M26j |
| M26j | Pallor and shortness of breath (both present)? | Yes (1)  No (0)  Don’t know (9) | Please press on one box only | Go to M26k |
| M26k | Did she suffer from any other illness? | Yes (1)  No (0)  Don’t know (9) | Please press on one box only | Go to M27 |
| M27 | Did she die during labor, but undelivered? | Yes (1)  No (0)  Don’t know (9) | Please press on one box only | If M27=0,9 go to M28  If M27=1 go to M30 |
| M28 | Did she give birth recently? | Yes (1)  No (0)  Don’t know (9) | Please press on one box only | If M28=1 go to M29  If M28=0,9 go to M40 |
| M29 | How many days after giving birth did she die? | Number pad  Don’t know (999) | Please use the number pad | Go to M30 |
| M30 | Was there excessive bleeding on the day labor started? | Yes (1)  No (0)  Don’t know (9) | Please press on one box only | Go to M31 |
| M31 | Was there excessive bleeding during labor before the delivering of the baby? | Yes (1)  No (0)  Don’t know (9) | Please press on one box only | Go to M32 |
| M32 | Was there excessive bleeding after delivering the baby? | Yes (1)  No (0)  Don’t know (9) | Please press on one box only | Go to M33 |
| M33 | Did she have difficulty in delivering the placenta? | Yes (1)  No (0)  Don’t know (9) | Please press on one box only | Go to M34 |
| M34 | Was she in labor for unusually long (more than 24 hours)? | Yes (1)  No (0)  Don’t know (9) | Please press on one box only | Go to M35 |
| M35 | Was it a normal vaginal delivery? | Yes (1)  No (0)  Don’t know (9) | Please press on one box only | If M35=0 go to M36  If M35=1,9 go to M37 |
| M36 | What type of delivery was it? | Forceps/ vacuum (1)  Caesarean section (2)  Other (3)  Don’t know (9) | Please press on one box only | Go to M37 |
| M37 | Did she have foul smelling vaginal discharge during delivery? | Yes (1)  No (0)  Don’t know (9) | Please press on one box only | Go to M38 |
| M38 | Where did she give birth? | Hospital (1)  Other health facility (2)  Home (3)  Other (4)  Don’t know (9) | Please press on one box only | Go to M39 |
| M39 | Who conducted the delivery? | Doctor (1)  Nurse/ midwife (2)  Traditional birth attendant (3)  Relative (4)  Mother by herself (5)  Other (6)  Don’t know (9) | Please press on one box only | Go to M40 |
| M40 | Did she experience an abortion recently? | Yes (1)  No (0)  Don’t know (9) | Please press on one box only | If M40=1 go to M41  If M40=0,9 go to M47 |
| M41 | Did she die during the abortion? | Yes (1)  No (0)  Don’t know (9) | Please press on one box only | If M41=0 go to M42  If M41=1,9 go to M44 |
| M42 | How many days before death did she have the abortion? | Number pad  Don’t know (999) | Please use the number pad | Go to M43 |
| M43 | How many months pregnant was she when she had the abortion? | Number pad  Don’t know (999) | Please use the number pad | Go to M44 |
| M44 | Did she have heavy bleeding after the abortion? | Yes (1)  No (0)  Don’t know (9) | Please press on one box only | Go to M45 |
| M45 | Did the abortion occur by itself, spontaneously? | Yes (1)  No (0)  Don’t know (9) | Please press on one box only | If M45=0 go to M46  If M45=1,9 go to M47 |
| M46 | Did she take medicine or treatment to induce the abortion? | Yes (1)  No (0)  Don’t know (9) | Please press on one box only | Go to M47 |
| M47 | For how long was she ill before she died? (in months) | Number pad  Less than 1 month (0)  Don’t know (999) | Please use the number pad | Go to M48 |
| M48 | Did she have a fever? | Yes (1)  No (0)  Don’t know (9) | Please press on one box only | If M48=1 go to M49  If M48=0,9 go to M53 |
| M49 | For how long did she have a fever? (in days) | Number pad  Don’t know (999) | Please use the number pad | Go to M50 |
| M50 | Was the fever continuous or on and off? | Continuous (1)  On and off (2)  Don’t know (9) | Please press on one box only | Go to M51 |
| M51 | Did she have fever only at night? | Yes (1)  No (0)  Don’t know (9) | Please press on one box only | Go to M52 |
| M52 | Did she have chills/ rigor? | Yes (1)  No (0)  Don’t know (9) | Please press on one box only | Go to M53 |
| M53 | Did she have a cough? | Yes (1)  No (0)  Don’t know (9) | Please press on one box only | If M53=1 go to M54  If M53=0,9 go to M59 |
| M54 | For how long did she have a cough? (in days) | Number pad  Don’t know (999) | Please use the number pad | Go to M55 |
| M55 | Was the cough severe? | Yes (1)  No (0)  Don’t know (9) | Please press on one box only | Go to M56 |
| M56 | Was the cough productive with sputum (saliva and mucus)? | Yes (1)  No (0)  Don’t know (9) | Please press on one box only | Go to M57 |
| M57 | Did she cough out blood? | Yes (1)  No (0)  Don’t know (9) | Please press on one box only | Go to M58 |
| M58 | Did she have night sweats? | Yes (1)  No (0)  Don’t know (9) | Please press on one box only | Go to M59 |
| M59 | Did she have breathlessness? | Yes (1)  No (0)  Don’t know (9) | Please press on one box only | If M59=1 go to M60  If M59=0,9 go to M64 |
| M60 | For how long did she have breathlessness? (in days) | Number pad  Don’t know (999) | Please use the number pad | Go to M61 |
| M61 | Was she unable to carry out daily routines due to breathlessness? | Yes (1)  No (0)  Don’t know (9) | Please press on one box only | Go to M62 |
| M62 | Was she breathless while lying flat? | Yes (1)  No (0)  Don’t know (9) | Please press on one box only | Go to M63 |
| M63 | Did she have wheezing (breathing with a whistling or rattling sound in the chest)? | Yes (1)  No (0)  Don’t know (9) | Please press on one box only | Go to M64 |
| M64 | Did she have chest pain? | Yes (1)  No (0)  Don’t know (9) | Please press on one box only | If M64=1 go to M65  If M64=0,9 go to M74 |
| M65 | For how long did she have chest pain? (in days) | Number pad  Don’t know (999) | Please use the number pad | Go to M66 |
| M66 | Did chest pain start suddenly or gradually? | Suddenly (1)  Gradually (2)  Don’t know (9) | Please press on one box only | Go to M67 |
| M67 | When she had severe chest pain, how long did it last? | Less than half an hour (1)  Half an hour to 24 hours (2)  Longer than 24 hours (3)  Don’t know (8) | Please press on one box only | Go to M68 |
| M68 | Was the chest pain located below the breastbone (sternum)? | Yes (1)  No (0)  Don’t know (9) | Please press on one box only | Go to M69 |
| M69 | Was the chest pain over the heart and did it spread to the left arm? | Yes (1)  No (0)  Don’t know (9) | Please press on one box only | Go to M70 |
| M70 | Was the chest pain located over the ribs (sides)? | Yes (1)  No (0)  Don’t know (9) | Please press on one box only | Go to M71 |
| M71 | Was the chest pain continuous or on and off? | Continuous (1)  On and off (2)  Don’t know (9) | Please press on one box only | Go to M72 |
| M72 | Did the chest pain get worse while coughing? | Yes (1)  No (0)  Don’t know (9) | Please press on one box only | Go to M73 |
| M73 | Did she have palpitations? | Yes (1)  No (0)  Don’t know (9) | Please press on one box only | Go to M74 |
| M74 | Did she have diarrhea? | Yes (1)  No (0)  Don’t know (9) | Please press on one box only | If M74=1 go to M75  If M74=0,9 go to M79 |
| M75 | For how long did she have diarrhea? (in days) | Number pad  Don’t know (999) | Please use the number pad | Go to M76 |
| M76 | Was the diarrhea continuous or on and off? | Continuous (1)  On and off (2)  Don’t know (9) | Please press on one box only | Go to M77 |
| M77 | Was there blood in the stool at any time during the final illness? | Yes (1)  No (0)  Don’t know (9) | Please press on one box only | Go to M78 |
| M78 | When the diarrhea was most severe, how many times did she pass stool in a day? | Number pad  Don’t know (999) | Please use the number pad | Go to M79 |
| M79 | Did she vomit? | Yes (1)  No (0)  Don’t know (9) | Please press on one box only | If M79=1 go to M80  If M79=0,9 go to M83 |
| M80 | For how long did she vomit? (in days) | Number pad  Don’t know (999) | Please use the number pad | Go to M81 |
| M81 | Did the vomit look like a coffee-colored fluid or bright red/ blood red or some other? | Coffee-colored fluid (1)  Bright red/ Blood red (2)  Other (3)  Don’t know (9) | Please press on one box only | Go to M82 |
| M82 | When the vomiting was most severe, how many times did she vomit in a day? | Number pad  Don’t know (999) | Please use the number pad | If M24=0 and M27=0 and M41=0 go to M83  If M24=1 or M27=1 or M41=1 go to M92 |
| M83 | Did she have abdominal pain? | Yes (1)  No (0)  Don’t know (9) | Please press on one box only | If M83=1 go to M84  If M83=0,9 go to M85 |
| M84 | For how long did she have abdominal pain? (in days) | Number pad  Don’t know (999) | Please use the number pad | Go to M85 |
| M85 | Did she have abdominal bloating? | Yes (1)  No (0)  Don’t know (9) | Please press on one box only | If M85=1 go to M86  If M85=0,9 go to M89 |
| M86 | For how long did she have abdominal bloating? (in days) | Number pad  Don’t know (999) | Please use the number pad | Go to M87 |
| M87 | Did the bloating develop rapidly within days or gradually over months? | Rapidly within days (1)  Gradually over months (2)  Don’t know (9) | Please press on one box only | Go to M88 |
| M88 | Was there a period of a day or longer during which she did not pass any stool? | Yes (1)  No (0)  Don’t know (9) | Please press on one box only | Go to M89 |
| M89 | Did she have any mass in her abdomen? | Yes (1)  No (0)  Don’t know (9) | Please press on one box only | If M89=1 go to M90  If M89=0,9 go to M92 |
| M90 | For how long did she have the mass in the abdomen? (in months) | Number pad  Less than 1 month (0)  Don’t know (999) | Please use the number pad | Go to M91 |
| M91 | Where in the abdomen was the mass located? | Right upper abdomen (1)  Left upper abdomen (2)  Lower abdomen (3)  All over abdomen (4)  Don’t know (9) | Please press on one box only | Go to M92 |
| M92 | Did she have difficulty or pain while swallowing solids? | Yes (1)  No (0)  Don’t know (9) | Please press on one box only | If M92=1 go to M93  If M92=0,9 go to M94 |
| M93 | For how long did she have difficulty or pain while swallowing solids? (in days) | Number pad  Don’t know (999) | Please use the number pad | Go to M94 |
| M94 | Did she have difficulty or pain while swallowing liquids? | Yes (1)  No (0)  Don’t know (9) | Please press on one box only | If M94=1 go to M95  If M94=0,9 go to M96 |
| M95 | For how long did she have difficulty or pain while swallowing liquids? (in days) | Number pad  Don’t know (999) | Please use the number pad | Go to M96 |
| M96 | Did she have headache? | Yes (1)  No (0)  Don’t know (9) | Please press on one box only | If M96=1 go to M97  If M96=0,9 go to M99 |
| M97 | For how long did she have headache? (in months) | Number pad  Less than 1 month (0)  Don’t know (999) | Please use the number pad | Go to M98 |
| M98 | Was the headache severe? | Yes (1)  No (0)  Don’t know (9) | Please press on one box only | Go to M99 |
| M99 | Did she have a stiff or painful neck? | Yes (1)  No (0)  Don’t know (9) | Please press on one box only | If M99=1 go to M100  If M99=0,9 go to M101 |
| M100 | For how long did she have a stiff or painful neck? (in days) | Number pad  Don’t know (999) | Please use the number pad | Go to M101 |
| M101 | Did she have mental confusion? | Yes (1)  No (0)  Don’t know (9) | Please press on one box only | If M101=1 go to M102  If M101=0,9 go to M104 |
| M102 | For how long did she have mental confusion? (in days) | Number pad  Don’t know (999) | Please use the number pad | Go to M103 |
| M103 | Did the mental confusion start suddenly, quickly within a single day, or slowly over many days? | Suddenly (1)  Within a day (fast) (2)  Slowly (many days) (3)  Don’t know (9) | Please press on one box only | Go to M104 |
| M104 | Did she become unconscious? | Yes (1)  No (0)  Don’t know (9) | Please press on one box only | If M104=1 go to M105  If M104=0,9 go to M107 |
| M105 | For how long was she unconscious? (in days) | Number pad  Don’t know (999) | Please use the number pad | Go to M106 |
| M106 | Did the unconsciousness start suddenly, quickly within a single day, or slowly over many days? | Suddenly (1)  Within a day (fast) (2)  Slowly (many days) (3)  Don’t know (9) | Please press on one box only | Go to M107 |
| M107 | Did she have convulsions (sudden, violent, irregular movement of the body)? | Yes (1)  No (0)  Don’t know (9) | Please press on one box only | If M107=1 go to M108  If M107=0,9 go to M109 |
| M108 | For how long did she have convulsions? (in days) | Number pad  Don’t know (999) | Please use the number pad | Go to M109 |
| M109 | Was she unable to open the mouth? | Yes (1)  No (0)  Don’t know (9) | Please press on one box only | If M109=1 go to M110  If M109=0,9 go to M111 |
| M110 | For how long was she unable to open her mouth? (in days) | Number pad  Don’t know (999) | Please use the number pad | Go to M111 |
| M111 | Did she have stiffness of the whole body? | Yes (1)  No (0)  Don’t know (9) | Please press on one box only | If M111=1 go to M112  If M111=0,9 go to M113 |
| M112 | For how long did she have stiffness of the whole body? (in days) | Number pad  Don’t know (999) | Please use the number pad | Go to M113 |
| M113 | Did she have paralysis of one side of the body? | Yes (1)  No (0)  Don’t know (9) | Please press on one box only | If M113=1 go to M114  If M113=0,9 go to M116 |
| M114 | For how long did she have paralysis of one side of the body? (in days) | Number pad  Don’t know (999) | Please use the number pad | Go to M115 |
| M115 | Did the paralysis of one side of the body start suddenly, quickly within a single day, or slowly over many days? | Suddenly (1)  Within a day (fast) (2)  Slowly (many days) (3)  Don’t know (9) | Please press on one box only | Go to M116 |
| M116 | Did she have paralysis of the lower limbs? | Yes (1)  No (0)  Don’t know (9) | Please press on one box only | If M116=1 go to M117  If M116=0,9 go to M119 |
| M117 | For how long did she have paralysis of the lower limbs? (in days) | Number pad  Don’t know (999) | Please use the number pad | Go to M118 |
| M118 | Did the paralysis of the lower limbs start suddenly, quickly within a single day, or slowly over many days? | Suddenly (1)  Within a day (fast) (2)  Slowly (many days) (3)  Don’t know (9) | Please press on one box only | Go to M119 |
| M119 | Was there change in the color of urine? | Yes (1)  No (0)  Don’t know (9) | Please press on one box only | If M119=1 go to M120  If M119=0,9 go to M121 |
| M120 | For how long did she have the change in the color of urine? (in days) | Number pad  Don’t know (999) | Please use the number pad | Go to M121 |
| M121 | During the final illness did she ever pass blood in the urine? | Yes (1)  No (0)  Don’t know (9) | Please press on one box only | If M121=1 go to M122  If M121=0,9 go to M123 |
| M122 | For how long did she pass blood in the urine? (in days) | Number pad  Don’t know (999) | Please use the number pad | Go to M123 |
| M123 | Was there any change in the amount of urine she passed daily? | Yes (1)  No (0)  Don’t know (9) | Please press on one box only | If M123=1 go to M124  If M123=0,9 go to M126 |
| M124 | For how long did she have the change in the amount of urine passed daily? (in days) | Number pad  Don’t know (999) | Please use the number pad | Go to M125 |
| M125 | Did she pass too much urine, too little urine, or no urine at all? | Too much (1)  Too little (2)  No urine al all (3)  Don’t know (9) | Please press on one box only | Go to M126 |
| M126 | During the illness that led to death, did she have any skin rash? | Yes (1)  No (0)  Don’t know (9) | Please press on one box only | If M126=1 go to M127  If M126=0,9 go to M130 |
| M127 | For how long did she have the skin rash? (in days) | Number pad  Don’t know (999) | Please use the number pad | Go to M128a |
| Intro M128a | Was the rash on: |  |  |  |
| M128a | The face? | Yes (1)  No (0)  Don’t know (9) | Please press on one box only | Go to M128b |
| M128b | The trunk? | Yes (1)  No (0)  Don’t know (9) | Please press on one box only | Go to M128c |
| M128c | The arms and legs? | Yes (1)  No (0)  Don’t know (9) | Please press on one box only | Go to M128d |
| M128d | Any other place? | Yes (1)  No (0)  Don’t know (9) | Please press on one box only | Go to M129 |
| M129 | What did the rash look like? | Measles rash (1)  Rash with clear fluid (2)  Rash with pus (3)  Don’t know (9) | Please press on one box only | Go to M130 |
| M130 | Did she complain of burning sensation of the legs? | Yes (1)  No (0)  Don’t know (9) | Please press on one box only | Go to M131a |
| M131a | Did she have red eyes? | Yes (1)  No (0)  Don’t know (9) | Please press on one box only | Go to M131b |
| M131b | Did she have bleeding from the nose, mouth or anus? | Yes (1)  No (0)  Don’t know (9) | Please press on one box only | Go to M132a |
| M132a | Did she ever have shingles (herpes zoster)? | Yes (1)  No (0)  Don’t know (9) | Please press on one box only | Go to M132b |
| M132b | Did she complain of itching of the skin? | Yes (1)  No (0)  Don’t know (9) | Please press on one box only | Go to M132c |
| M132c | Did the skin become very dry and scaly/ flaky? | Yes (1)  No (0)  Don’t know (9) | Please press on one box only | Go to M133 |
| M133 | Did she have weight loss? | Yes (1)  No (0)  Don’t know (9) | Please press on one box only | If M133=1 go to M134  If M133=0,9 go to M136 |
| M134 | For how long did she have weight loss? (in months) | Number pad  Less than 1 month (0)  Don’t know (999) | Please use the number pad | Go to M135 |
| M135 | Did she look very thin and wasted? | Yes (1)  No (0)  Don’t know (9) | Please press on one box only | Go to M136 |
| M136 | Did she have mouth sores or white patches in the mouth or on the tongue? | Yes (1)  No (0)  Don’t know (9) | Please press on one box only | If M136=1 go to M137  If M136=0,9 go to M138 |
| M137 | For how long did she have mouth sores or white patches in the mouth or on the tongue? (days) | Number pad  Don’t know (999) | Please use the number pad | Go to M138 |
| M138 | Did she have any swelling? | Yes (1)  No (0)  Don’t know (9) | Please press on one box only | If M138=1 go to M139  If M138=0,9 go to M141 |
| M139 | For how long did she have the swelling? (days) | Number pad  Don’t know (999) | Please use the number pad | Go to M140 |
| Intro M140 | Was the swelling on: |  |  |  |
| M140a | The face? | Yes (1)  No (0)  Don’t know (9) | Please press on one box only | Go to M140b |
| M140b | The joints? | Yes (1)  No (0)  Don’t know (9) | Please press on one box only | Go to M140c |
| M140c | The arms? | Yes (1)  No (0)  Don’t know (9) | Please press on one box only | Go to M140d |
| M140d | The legs? | Yes (1)  No (0)  Don’t know (9) | Please press on one box only | Go to M140e |
| M140e | The whole body? | Yes (1)  No (0)  Don’t know (9) | Please press on one box only | Go to M140f |
| M140f | The vagina? (swelling growing out of the vagina) | Yes (1)  No (0)  Don’t know (9) | Please press on one box only | Go to M140g |
| M140g | Any other place? | Yes (1)  No (0)  Don’t know (9) | Please press on one box only | Go to M141 |
| M141 | Did she have any lumps? | Yes (1)  No (0)  Don’t know (9) | Please press on one box only | If M141=1 go to M142  If M141=0,9 go to M144 |
| M142 | For how long did she have the lumps? (months) | Number pad  Less than 1 month (0)  Don’t know (999) | Please use the number pad | Go to M143 |
| Intro M143 | Were the lumps on: |  |  |  |
| M143a | The neck? | Yes (1)  No (0)  Don’t know (9) | Please press on one box only | Go to M143b |
| M143b | The armpit? | Yes (1)  No (0)  Don’t know (9) | Please press on one box only | Go to M143c |
| M143c | The groin? | Yes (1)  No (0)  Don’t know (9) | Please press on one box only | Go to M143d |
| M140d | Any other place? | Yes (1)  No (0)  Don’t know (9) | Please press on one box only | Go to M144 |
| M144 | Did she have yellow discoloration of the eyes? (days) | Yes (1)  No (0)  Don’t know (9) | Please press on one box only | If M144=1 go to M144  If M144=0,9 go to M146 |
| M145 | For how long did she have yellow discoloration of the eyes? (days) | Number pad  Don’t know (999) | Please use the number pad | Go to M146a |
| M146a | Did she look pale (thinning/ lack of blood) or have pale palms, eyes or nail beds? | Yes (1)  No (0)  Don’t know (9) | Please press on one box only | If M146a=1 go to M146b  If M146a=0,9 go to M147 |
| M146b | For how long did she look pale or have pale palms, eyes or nail beds? (days) | Number pad  Don’t know (999) | Please use the number pad | Go to M147 |
| M147 | Did the colour of her hair change? | Yes (1)  No (0)  Don’t know (9) | Please press on one box only | Go to M148 |
| M148 | Did she have an ulcer, abscess, or sore anywhere on the body? | Yes (1)  No (0)  Don’t know (9) | Please press on one box only | If M148=1 go to M149  If M148=0,9 go to M150 |
| M149 | For how long did she have the ulcer, abscess, or sore? (days) | Number pad  Don’t know (999) | Please use the number pad | Go to M150 |
| M150 | Did she receive any treatment for the illness that led to death? | Yes (1)  No (0)  Don’t know (9) | Please press on one box only | If M150=1 go to M151a  If M150=0,9 go to M152 |
| Intro M151a | What type of treatment did she receive? |  |  |  |
| M151a | Oral rehydration salts and/or intravenous fluids (drip) treatment? | Yes (1)  No (0)  Don’t know (9) | Please press on one box only | Go to M151b |
| M151b | Blood transfusion? | Yes (1)  No (0)  Don’t know (9) | Please press on one box only | Go to M151c |
| M151c | Treatment/ food through a tube passed through the nose? | Yes (1)  No (0)  Don’t know (9) | Please press on one box only | Go to M151d |
| M151d | Any other treatment? | Yes (1)  No (0)  Don’t know (9) | Please press on one box only | Go to M152 |
| M152 | Did she have any operation for the illness? | Yes (1)  No (0)  Don’t know (9) | Please press on one box only | If M152=1 go to M153  If M152=0,9 go to M155 |
| M153 | How long before death did she have the operation? (in days) | Number pad  Don’t know (999) | Please use the number pad | Go to M154a |
| M154 | On what part of the body was the operation? | Abdomen (1)  Chest (2)  Head (3)  Other (4)  Don’t know (9) | Please press on one box only | Go to M155 |
| M155 | During her lifetime, did the mother usually drink a lot of alcohol? | Yes (1)  No (0)  Don’t know (9) | Please press on one box only | Go to M156 |
| M156 | Do you have the mother’s death certificate? | Yes (1)  No (0) | Please press on one box only | If M156=1 go to M157  If M156=0 SAVE INTERVIEW AND FILL IN THE GREEN COLOR PAPER. |
| M157 | Can I see the death certificate? | Yes (1)  No (0) | Please press on one box only | SAVE INTERVIEW AND FILL IN THE GREEN COLOR PAPER. |
|  | **Section V. Infant’s verbal autopsy (infant less than 4 weeks)** |  |  |  |
| V1 | Did you live with the baby in the period leading to her/his death? | Yes (1)  No (0) | Please press on one box only | If V1=1 go to V2  If V1=0 FINISH |
| Intro V2 | I know it may be difficult to talk about this, but I will ask you some questions about the events surrounding the death of your baby. |  |  |  |
| V2 | Where did s/he die? | Hospital (1)  Other health facility (2)  Home (3)  Other (4)  Don’t know (8) | Please press on one box only | Go to V7a |
| Intro V7a | I would like to ask you some questions concerning yourself and symptoms that the baby had at birth and shortly after. Some of these questions may not appear to be directly related to the baby’s death. Please bear with me and answer all the questions. They will help us to get a clear picture of all possible symptoms that the baby had. |  |  |  |
| Intro V7a | During the pregnancy did you suffer from any of the following illnesses: |  |  |  |
| V7a | High blood pressure? | Yes (1)  No (0)  Don’t know (8) | Please press on one box only | Go to V7b |
| V7b | Heart disease? | Yes (1)  No (0)  Don’t know (8) | Please press on one box only | Go to V7c |
| V7c | Diabetes? | Yes (1)  No (0)  Don’t know (8) | Please press on one box only | Go to V7d |
| V7d | Epilepsy/convulsion (sudden, violent, irregular movement of the body)? | Yes (1)  No (0)  Don’t know (8) | Please press on one box only | Go to V7e |
| V7e | Any other medically diagnosed illness? | Yes (1)  No (0)  Don’t know (8) | Please press on one box only | Go to V8a |
| Intro V8a | During the last 3 months of pregnancy did you suffer from any of the following illnesses: |  |  |  |
| V8a | Vaginal bleeding? | Yes (1)  No (0)  Don’t know (8) | Please press on one box only | Go to V8b |
| V8b | Smelly vaginal discharge? | Yes (1)  No (0)  Don’t know (8) | Please press on one box only | Go to V8c |
| V8c | Puffy face? | Yes (1)  No (0)  Don’t know (8) | Please press on one box only | Go to V8d |
| V8d | Headache? | Yes (1)  No (0)  Don’t know (8) | Please press on one box only | Go to V8e |
| V8e | Blurred vision? | Yes (1)  No (0)  Don’t know (8) | Please press on one box only | Go to V8f |
| V8f | Convulsion (sudden, violent, irregular movement of the body)? | Yes (1)  No (0)  Don’t know (8) | Please press on one box only | Go to V8g |
| V8g | Febrile illness? | Yes (1)  No (0)  Don’t know (8) | Please press on one box only | Go to V8h |
| V8h | Severe abdominal pain that was not labour pain? | Yes (1)  No (0)  Don’t know (8) | Please press on one box only | Go to V8j |
| V8j | Pallor and shortness of breath (both present)? | Yes (1)  No (0)  Don’t know (8) | Please press on one box only | Go to V8k |
| V8k | Other | Yes (1)  No (0)  Don’t know (8) | Please press on one box only | Go to V9 |
| V9 | Was the baby a single or multiple birth? | Singleton (1)  Twin (2)  Triplet or more (3)  Don’t know (8) | Please press on one box only | If V9=1,8 go to V10  If V9=2,3 go to V11 |
| V10 | What was the birth order of the child that died? | First (1)  Second (2)  Third or higher (3) | Please press on one box only | Go to V11 |
| V11 | When did the water break? | Before labor started (1)  During labor (2)  Don’t remember (8) | Please press on one box only | Go to V12 |
| V12 | How many hours after the water broke was the baby born? | Less than 24 hours (1)  24 hours or more (2)  Don’t remember (8) | Please press on one box only | Go to V13 |
| V13 | Was the water foul smelling? | Yes (1)  No (0)  Don’t know/ don’t remember (8) | Please press on one box only | Go to V14 |
| V14 | Did the baby stop moving in the womb? | Yes (1)  No (0)  Don’t know/ don’t remember (8) | Please press on one box only | If V14=1 go to V15  If V14=0,8 go to V16 |
| V15 | When did the baby stop moving in the womb? | Before labor started (1)  During labor (2)  Don’t know/ don’t remember (8) | Please press on one box only | Go to V16 |
| V16 | Did the birth attendant listen for fetal heart sounds during labor? | Yes (1)  No (0)  Don’t know/ don’t remember (8) | Please press on one box only | If V16=1 go to V17  If V16=0,8 go to V18 |
| V17 | Were fetal heart sounds present? | Yes (1)  No (0)  Don’t know/ don’t remember (8) | Please press on one box only | Go to V18 |
| V18 | Was there excess bleeding on the day labor started? | Yes (1)  No (0)  Don’t know/ don’t remember (8) | Please press on one box only | Go to V19 |
| V19 | Did you have a fever on the day labor started? | Yes (1)  No (0)  Don’t know/ don’t remember (8) | Please press on one box only | Go to V20 |
| V20 | How long did the labor pains last? | Less than 12 hours (1)  12-23 hours (2)  24 hours or more (3)  Don’t remember (8) | Please press on one box only | Go to V21 |
| V21 | Was it a normal vaginal delivery? | Yes (1)  No (0)  Don’t know (8) | Please press on one box only | If V21=1 go to V22  If V21=0,8 go to V23 |
| V22 | What type of delivery was it? | Forceps/vacuum (1)  Caesarean section (2)  Other (3)  Don’t know (8) | Please press on one box only | Go to V23 |
| V23 | Which part of the baby came first? | Head (1)  Bottom (2)  Feet (3)  Arm/hand (4)  Other (6)  Don’t know/ don’t remember (8) | Please press on one box only | Go to V24 |
| V24 | Did the umbilical cord come out before the baby was born? | Yes (1)  No (0)  Don’t know/ don’t remember (8) | Please press on one box only | Go to V25 |
| Intro V25 | Now I will ask you a few questions about the baby soon after the birth. |  |  |  |
| V25 | What was the size of the baby at birth? | Smaller than normal (1)  Normal (2)  Larger than normal (3)  Don’t know/ don’t remember (8) | Please press on one box only | Go to V26 |
| V26 | Was the baby premature? | Yes (1)  No (0)  Don’t know (8) | Please press on one box only | If V26=1 go to V27  If V26=0,8 go to V28 |
| V27 | How many weeks along was the pregnancy? | Number pad  Don’t remember (88) | Please use the number pad | Go to V28 |
| V28 | What was the birth weight of the baby? (in kilograms) | Number pad  Don’t remember (88) | Please use the number pad | Go to V29 |
| V29 | Was anything applied to the umbilical cord stump after birth? | Yes (1)  No (0)  Don’t know/ don’t remember (8) | Please press on one box only | Go to V30 |
| V30 | Were there any signs of injury or broken bones? | Yes (1)  No (0)  Don’t know/ don’t remember (8) | Please press on one box only | Go to V31 |
| V31 | Was there any sign of paralysis? | Yes (1)  No (0)  Don’t know/ don’t remember (8) | Please press on one box only | Go to V32 |
| V32 | Did the baby have any malformation? | Yes (1)  No (0)  Don’t know/ don’t remember (8) | Please press on one box only | If V32=1 go to V33  If V32=0,8 go to V34 |
| V33 | What kind of malformation did the baby have? | Swelling/defect on back (1)  Very large head (2)  Very small head (3)  Defect of lip and/or palate (4)  Other malformation (6)  Don’t know/ don’t remember (8) | Please press on one box only | Go to V34 |
| V34 | What was the color of the baby at birth? | Normal (1)  Pale (2)  Blue (3)  Don’t know/ don’t remember (8) | Please press on one box only | Go to V35 |
| V35 | Was the baby given assistance to breathe? | Yes (1)  No (0)  Don’t know/ don’t remember (8) | Please press on one box only | Go to V41 |
| Intro V41 | Now I will ask you about any injuries or accidents your baby may have had. |  |  |  |
| V41 | Did the baby suffer from any injury or accident that led to her/his death? | Yes (1)  No (0)  Don’t know/ don’t remember (8) | Please press on one box only | If V41=1 go to V42  If V41=0,8 go to V44 |
| V42 | What kind of injury or accident did the baby suffer? | Road traffic accident (1)  Fall (2)  Drowning (3)  Poisoning (4)  Burns (5)  Violence/Assault (6)  Other (7)  Don’t know/ don’t remember (8) | Please press on one box only | Go to V43 |
| V43 | Was the injury or accident intentionally inflicted by someone else? | Yes (1)  No (0)  Don’t know/ don’t remember (8) | Please press on one box only | Go to V44 |
| V44 | Did the baby suffer from any animal or insect bite that led to her/his death? | Yes (1)  No (0)  Don’t know/ don’t remember (8) | Please press on one box only | If V44=1 go to V45  If V44=0,8 go to V46 |
| V45 | What type of animal or insect? | Dog (1)  Snake (2)  Insect (3)  Other (4)  Don’t know/ don’t remember (8) | Please press on one box only | Go to V46 |
| Intro V46 | Now I will ask you about various illnesses your baby may have had soon after the birth. |  |  |  |
| V46 | Was the baby ever able to suckle or bottle-feed? | Yes (1)  No (0)  Don’t know (8) | Please press on one box only | If V46=1 go to V47  If V46=0,8 go to V50 |
| V47 | How soon after birth did the baby suckle or bottle-feed? | Number pad  Less than 1 day (0)  Don’t remember (8) | Please use the number pad | Go to V48 |
| V48 | Did the baby stop suckling or bottle-feeding? | Yes (1)  No (0)  Don’t know (8) | Please press on one box only | If V48=1 go to V49  If V48=0,8 go to V50 |
| V49 | How many days after birth did the baby stop suckling or bottle-feeding? | Number pad  Don’t remember (88) | Please use the number pad | Go to V50 |
| V50 | Was the breastfeeding exclusive? | Yes (1)  No (0)  Don’t remember (8) | Please press on one box only | Go to V51 |
| V51 | Did the baby have convulsions (sudden, violent, irregular movement of the body)? | Yes (1)  No (0)  Don’t remember (8) | Please press on one box only | If V51=1 go to V52  If V51=0,8 go to V53 |
| V52 | How soon after the birth did the convulsions start? (in days) | Number pad  Don’t remember (88) | Please use the number pad | Go to V53 |
| V53 | Did the baby become stiff and arched backwards? | Yes (1)  No (0)  Don’t remember (8) | Please press on one box only | Go to V54 |
| V54 | Did the child have bulging of the fontanelle (a space between the bones of the skull)? | Yes (1)  No (0)  Don’t remember (8) | Please press on one box only | If V54=1 go to V55  If V55=0,8 go to V56 |
| V55 | How many days after birth did the baby have the bulging? | Number pad  Don’t remember (88) | Please use the number pad | Go to V56 |
| V56 | Did the baby become unresponsive or unconscious? | Yes (1)  No (0)  Don’t remember (8) | Please press on one box only | If V56=1 go to V57  If V56=0,8 go to V58 |
| V57 | How many days after birth did the baby become unresponsive or unconscious?' | Number pad  Don’t remember (88) | Please use the number pad | Go to V58 |
| V58 | Did the baby have a fever? | Yes (1)  No (0)  Don’t remember (8) | Please press on one box only | If V58=1 go to V59  If V58=0,8 go to V60 |
| V59 | How many days after birth did the baby have a fever? | Number pad  Don’t remember (88) | Please use the number pad | Go to V60 |
| V60 | Did the baby become cold to the touch? | Yes (1)  No (0)  Don’t know (8) | Please press on one box only | If V60=1 go to V61  If V60=0,8 go to V62 |
| V61 | How many days after birth did the baby become cold to the touch? | Number pad  Don’t remember (88) | Please use the number pad | Go to V62 |
| V62 | Did the baby have a cough? | Yes (1)  No (0)  Don’t remember (8) | Please press on one box only | If V62=1 go to V63  If V62=0,8 go to V64 |
| V63 | How many days after birth did the baby start to cough? | Number pad  Don’t remember (88) | Please use the number pad | Go to V64 |
| V64 | Did the baby have fast breathing? | Yes (1)  No (0)  Don’t remember (8) | Please press on one box only | If V64=1 go to V65  If V64=0,8 go to V66 |
| V65 | How many days after birth did the baby start breathing fast? | Number pad  Don’t remember (88) | Please use the number pad | Go to V66 |
| V66 | Did the baby have difficulty breathing? | Yes (1)  No (0)  Don’t remember (8) | Please press on one box only | If V66=1 go to V67  If V66=0,8 go to V68 |
| V67 | How many days after birth did the baby start having difficulty in breathing? | Number pad  Don’t remember (88) | Please use the number pad | Go to V68 |
| V68 | Did the baby have chest indrawing (the lower ribs on both sides of the chest pulling inwards when the child was breathing in)? | Yes (1)  No (0)  Don’t remember (8) | Please press on one box only | Go to V69 |
| V69 | Did the baby have grunting (short, deep, hoarse sounds)? [DEMONSTRATE] | Yes (1)  No (0)  Don’t remember (8) | Please press on one box only | Go to V70 |
| V70 | Did the baby have flaring of the nostrils? | Yes (1)  No (0)  Don’t remember (8) | Please press on one box only | Go to V71 |
| V71 | Did the baby have diarrhoea? | Yes (1)  No (0)  Don’t remember (8) | Please press on one box only | If V71=1 go to V72  If V71=0,8 go to V75 |
| V72 | How many days after birth did the baby have diarrhoea? | Number pad  Don’t remember (88) | Please use the number pad | Go to V73 |
| V73 | When the diarrhoea was most severe, how many times did the baby pass stools in a day? | Number pad  Don’t remember (88) | Please use the number pad | Go to V74 |
| V74 | Was there blood in the stools? | Yes (1)  No (0)  Don’t remember (8) | Please press on one box only | Go to V75 |
| V75 | Did the baby have vomiting? | Yes (1)  No (0)  Don’t remember (8) | Please press on one box only | If V75=1 go to V76  If V75=0,8 go to V78 |
| V76 | How many days after birth did vomiting start? | Number pad  Don’t remember (88) | Please use the number pad | Go to V77 |
| V77 | When the vomiting was most severe, how many times did the baby vomit in a day? | Number pad  Don’t remember (88) | Please use the number pad | Go to V78 |
| V78 | Did the baby have abdominal distension (bloating)? | Yes (1)  No (0)  Don’t remember (8) | Please press on one box only | If V78=1 go to V79  If V78=0,8 go to V80 |
| V79 | How many days after birth did the baby have abdominal distension? | Number pad  Don’t remember (8) | Please use the number pad | Go to V80 |
| V80 | Did the baby have redness or discharge from the umbilical cord stump? | Yes (1)  No (0)  Don’t remember (8) | Please press on one box only | Go to V81 |
| V81 | Did the baby have a pustular skin rash (small blisters or pimples containing pus)? | Yes (1)  No (0)  Don’t remember (8) | Please press on one box only | Go to V82 |
| V82 | Did the baby have yellow palms or soles? | Yes (1)  No (0)  Don’t remember (8) | Please press on one box only | If V82=1 go to V83  If V82=0,8 go to V85 |
| V83 | How many days after birth did the yellow palms or soles begin? | Number pad  Don’t remember (88) | Please use the number pad | Go to V84 |
| V84 | For how many days did the baby have yellow palms or soles? | Number pad  Don’t remember (88) | Please use the number pad | Go to V85 |
| Intro V85 | Now I will ask you a few questions about your health. |  |  |  |
| V85 | Did you receive tetanus toxoid (TT) vaccine? | Yes (1)  No (0)  Don’t know/ don’t remember (8) | Please press on one box only | If V85=1 go to V86  If V86=0,8 go to V87 |
| V86 | How many doses? | Number pad  Don’t know/ don’t remember (8) | Please use the number pad | Go to V87 |
| V87 | How is your health now? | Healthy (1)  Ill (2) | Please press on one box only | Go to V88 |
| Intro V88 | Now I will ask you some questions about the health services you may have accessed for your baby. |  |  |  |
| V88 | Did the baby receive any treatment for the illness that led to her/his death? | Yes (1)  No (0)  Don’t remember (8) | Please press on one box only | If V88=1 go to V89a  If V88=0,9 go to V91 |
| Intro V89a | Please tell me at which of the following places or facilities the baby received treatment during the illness that led to death: |  |  |  |
| V89a | Home? | Yes (1)  No (0)  Don’t remember (8) | Please press on one box only | Go to V89b |
| V89b | Traditional healer? | Yes (1)  No (0)  Don’t remember (8) | Please press on one box only | Go to V89c |
| V89c | Government clinic? | Yes (1)  No (0)  Don’t remember (8) | Please press on one box only | Go to V89d |
| V89d | Government hospital? | Yes (1)  No (0)  Don’t remember (8) | Please press on one box only | Go to V89e |
| V89e | Private clinic? | Yes (1)  No (0)  Don’t remember (8) | Please press on one box only | Go to V89f |
| V89f | Private hospital? | Yes (1)  No (0)  Don’t remember (8) | Please press on one box only | Go to V89g |
| V89g | Pharmacy, drug seller, store? | Yes (1)  No (0)  Don’t remember (8) | Please press on one box only | Go to V89h |
| V89h | Any other place or facility? | Yes (1)  No (0)  Don’t remember (8) | Please press on one box only | Go to V90 |
| V90 | In the month before death, how many contacts with formal health services did the baby have? | Number pad  Don’t remember (8) | Please use the number pad | Go to V91 |
| V91 | Do you have a death certificate for the baby? | Yes (1)  No (0) | Please press on one box only | If V91=1 go to V92  If V91=0 SAVE INTERVIEW AND FILL IN THE PINK COLOR PAPER. |
| V92 | Can I see the death certificate? | Yes (1)  No (0) | Please press on one box only | SAVE INTERVIEW AND FILL IN THE PINK COLOR PAPER. |
|  | **Section W. Infant’s verbal autopsy (infant over 4 weeks)** |  |  |  |
| W1 | Did you live with the baby in the period leading to her/his death? | Yes (1)  No (0) | Please press on one box only | If W1=1 go to W2  If W1=0 FINISH |
| Intro W2 | I would like to ask you some questions concerning yourself and symptoms that the baby had at birth and shortly after. Some of these questions may not appear to be directly related to the baby’s death. Please bear with me and answer all the questions. They will help us to get a clear picture of all possible symptoms that the baby had. |  |  |  |
| W2 | Where did s/he die? | Hospital (1)  Other health facility (2)  Home (3)  Other (4)  Don’t remember (8) | Please press on one box only | Go to W3a |
| Intro W3a | Please tell me if the baby suffered from any of the following illnesses: |  |  |  |
| W3a | Heart disease? | Yes (1)  No (0)  Don’t know/ don’t remember (8) | Please press on one box only | Go to W3b |
| W3b | Diabetes? | Yes (1)  No (0)  Don’t know/ don’t remember (8) | Please press on one box only | Go to W3c |
| W3c | Asthma? | Yes (1)  No (0)  Don’t know/ don’t remember (8) | Please press on one box only | Go to W3d |
| W3d | Epilepsy? | Yes (1)  No (0)  Don’t know/ don’t remember (8) | Please press on one box only | Go to W3e |
| W3e | Malnutrition? | Yes (1)  No (0)  Don’t know/ don’t remember (8) | Please press on one box only | Go to W3f |
| W3f | Cancer? | Yes (1)  No (0)  Don’t know/ don’t remember (8) | Please press on one box only | Go to W3g |
| W3g | Tuberculosis? | Yes (1)  No (0)  Don’t know/ don’t remember (8) | Please press on one box only | Go to W3h |
| W3h | HIV/AIDS? | Yes (1)  No (0)  Don’t know/ don’t remember (8) | Please press on one box only | Go to W3j |
| W3j | Any other medically diagnosed illness? | Yes (1)  No (0)  Don’t know/ don’t remember (8) | Please press on one box only. | Go to W4 |
| Intro W4 | Now I will ask you a few questions about any injuries or accidents your baby may have had. |  |  |  |
| W4 | Did s/he suffer from any injury or accident that led to her/his death? | Yes (1)  No (0)  Don’t remember (8) | Please press on one box only | If W4=1 go to W5  If W4=0,8 go to W7 |
| W5 | What kind of injury or accident did the baby suffer? | Road traffic accident (1)  Fall (2)  Drowning (3)  Poisoning (4)  Burns (5)  Violence/Assault (6)  Other (7) | Please press on one box only | Go to W6 |
| W6 | Was the injury or accident intentionally inflicted by someone else? | Yes (1)  No (0)  Don’t know/ don’t remember (8) | Please press on one box only | Go to W7 |
| W7 | Did the s/he suffer from any animal or insect bite that led to her/his death? | Yes (1)  No (0)  Don’t know (8) | Please press on one box only | If W7=1 go to W8  If W7=0,8 go to W9 |
| W8 | What type of animal or insect? | Dog (1)  Snake (2)  Insect (3)  Other (4)  Don’t know/ don’t remember (8) | Please press on one box only | If A5-Q1=1 year or under go to W9  If A5-Q1>1 year go to W15 |
| Intro W9 | Now I will ask you some questions about any symptoms the baby may have had during his/her final illness. |  |  |  |
| W9 | Was the child small at birth? | Yes (1)  No (0)  Don’t remember (8) | Please press on one box only | Go to W10 |
| W10 | Was the child born prematurely? | Yes (1)  No (0)  Don’t know/ don’t remember (8) | Please press on one box only | If W10=1 go to W11  If W10=0,8 go to W12 |
| W11 | How many weeks premature? | Number pad  Don’t remember (88) | Please use the number pad | Go to W12 |
| W12 | Was the child growing normally? | Yes (1)  No (0)  Don’t know/ don’t remember (8) | Please press on one box only | Go to W13 |
| W13 | Did the child have bulging of the fontanelle (a space between the bones of the skull)? | Yes (1)  No (0)  Don’t remember (8) | Please press on one box only | If W13=1 go to W14  If W13=0,8 go to W15 |
| W14 | For how many days before death did s/he have the bulging? | Number pad  Don’t remember (888) | Please use the number pad | Go to W15 |
| W15 | How is your health now? | Healthy (1)  Ill (2) | Please press on one box only | Go to W16 |
| W16 | For how long was the child ill before s/he died? (in days) | Number pad  Don’t remember (888) | Please use the number pad | Go to W17 |
| W17 | Did s/he have a fever? | Yes (1)  No (0)  Don’t remember (8) | Please press on one box only | If W17=1 go to W18  If W17=0,8 go to W21 |
| W18 | For how long did s/he have a fever? (in days) | Number pad  Don’t remember (888) | Please use the number pad | Go to W19 |
| W19 | Was the fever severe? | Yes (1)  No (0)  Don’t know/ don’t remember (8) | Please press on one box only | Go to W20 |
| W20 | Was the fever continuous or on and off? | Continuous (1)  On and off (2)  Don’t remember (8) | Please press on one box only | Go to W21 |
| W21 | Did s/he have chills/rigor? | Yes (1)  No (0)  Don’t remember (8) | Please press on one box only | Go to W22 |
| W22 | Did s/he have a cough? | Yes (1)  No (0)  Don’t remember (8) | Please press on one box only | If W22=1 go to W23  If W23=0,8 go to W26 |
| W23 | For how long did s/he have a cough? (in days) | Number pad  Don’t remember (888) | Please use the number pad | Go to W24 |
| W24 | Was the cough severe? | Yes (1)  No (0)  Don’t remember (8) | Please press on one box only | Go to W25 |
| W25 | Did the child vomit after s/he coughed? | Yes (1)  No (0)  Don’t remember (8) | Please press on one box only | Go to W26 |
| W26 | Did s/he have fast breathing? | Yes (1)  No (0)  Don’t remember (8) | Please press on one box only | If W26=1 go to W27  If W26=0,8 go to W32 |
| W27 | For how long did s/he have fast breathing? (in days) | Number pad  Don’t remember (888) | Please use the number pad | Go to W28 |
| W28 | Did s/he have difficulty in breathing? | Yes (1)  No (0)  Don’t remember (8) | Please press on one box only | If W28=1 go to W29  If W28=0,8 go to W34 |
| W29 | For how long did s/he have difficulty in breathing? (in days) | Number pad  Don’t remember (888) | Please use the number pad | Go to W30 |
| W30 | Did s/he have chest indrawing (the lower ribs on both sides of the chest pulling inwards when the child was breathing in)? | Yes (1)  No (0)  Don’t know/ don’t remember (8) | Please press on one box only | If W30=1 go to W31  If W30=0,8 go to W32 |
| W31 | For how long did s/he have chest indrawing? (in days) | Number pad  Don’t remember (888) | Please use the number pad | Go to W32 |
| W32 | Did s/he have noisy breathing (grunting or wheezing)? [DEMONSTRATE] | Yes (1)  No (0)  Don’t remember (8) | Please press on one box only | Go to W33 |
| W33 | Did s/he have flaring of the nostrils? | Yes (1)  No (0)  Don’t remember (8) | Please press on one box only | Go to W34 |
| W34 | Did s/he have diarrhoea? | Yes (1)  No (0)  Don’t remember (8) | Please press on one box only | If W34=1 go to W35  If W34=0,8 go to W38 |
| W35 | For how long did s/he have diarrhoea? (in days) | Number pad  Don’t remember (888) | Please use the number pad | Go to W36 |
| W36 | When the diarrhoea was most severe, how many times did s/he pass stool in a day? | Number pad  Don’t remember (88) | Please use the number pad | Go to W37 |
| W37 | At any time during the final illness was there blood in the stool? | Yes (1)  No (0)  Don’t remember (8) | Please press on one box only | Go to W38 |
| W38 | Did s/he vomit? | Yes (1)  No (0)  Don’t remember (8) | Please press on one box only | If W38=1 go to W39  If W38=0,8 go to W41 |
| W39 | For how long did s/he vomit? (in days) | Number pad  Don’t remember (888) | Please use the number pad | Go to W40 |
| W40 | When the vomiting was most severe, how many times did s/he vomit in a day? | Number pad  Don’t remember (88) | Please use the number pad | Go to W41 |
| W41 | Did s/he have abdominal pain? | Yes (1)  No (0)  Don’t remember (8) | Please press on one box only | If W41=1 go to W42  If W41=0,8 go to W44 |
| W42 | For how long did s/he have abdominal pain? (in days) | Number pad  Don’t remember (888) | Please use the number pad | Go to W43 |
| W43 | Was the abdominal pain severe? | Yes (1)  No (0)  Don’t remember (8) | Please press on one box only | Go to W44 |
| W44 | Did s/he have abdominal distension (bloating)? | Yes (1)  No (0)  Don’t remember (8) | Please press on one box only | If W44=1 go to W45  If W44=0,8 go to W48 |
| W45 | For how long did s/he have abdominal distension? (in days) | Number pad  Don’t remember (888) | Please use the number pad | Go to W46 |
| W46 | Did the distension develop rapidly within days or gradually over months? | Rapidly within days (1)  Gradually over months (2)  Don’t remember (8) | Please press on one box only | Go to W47 |
| W47 | Was there a period of a day or longer during which s/he did not pass any stool? | Yes (1)  No (0)  Don’t remember (8) | Please press on one box only | Go to W48 |
| W48 | Did s/he have any mass in the abdomen? | Yes (1)  No (0)  Don’t remember (8) | Please press on one box only | If W48=1 go to W49  If W48=0,8 go to W50 |
| W49 | For how long did s/he have the mass in the abdomen? (in days) | Number pad  Don’t remember (888) | Please use the number pad | Go to W50 |
| W50 | Did s/he have headache? | Yes (1)  No (0)  Don’t remember (8) | Please press on one box only | If W50=1 go to W51  If W50=0,8 go to W53 |
| W51 | For how long did s/he have headache? (in days) | Number pad  Don’t remember (888) | Please use the number pad | Go to W52 |
| W52 | Was the headache severe? | Yes (1)  No (0)  Don’t remember (8) | Please press on one box only | Go to W53 |
| W53 | Did s/he have a stiff or painful neck? | Yes (1)  No (0)  Don’t remember (8) | Please press on one box only | If W53=1 go to W54  If W53=0,8 go to W55 |
| W54 | For how long did s/he have a stiff or painful neck? (in days) | Number pad  Don’t remember (888) | Please use the number pad | Go to W55 |
| W55 | Did s/he become unconscious? | Yes (1)  No (0)  Don’t remember (8) | Please press on one box only | If W55=1 go to W56  If W55=0,8 go to W58 |
| W56 | For how long was s/he unconscious? (in days) | Number pad  Don’t remember (888) | Please use the number pad | Go to W57 |
| W57 | Did the unconsciousness start suddenly, quickly within a single day, or slowly over many days? | Suddenly (1)  Fast (in a day) (2)  Slowly (many days) (3)  Don’t remember (8) | Please press on one box only | Go to W58 |
| W58 | Did s/he have convulsions (sudden, violent, irregular movement of the body)? | Yes (1)  No (0)  Don’t remember (8) | Please press on one box only | If W58=1 go to W59  If W58=0,8 go to W60 |
| W59 | For how long did s/he have convulsions? (in days) | Number pad  Don’t remember (888) | Please use the number pad | Go to W60 |
| W60 | Did s/he have paralysis of the lower limbs? | Yes (1)  No (0)  Don’t remember (8) | Please press on one box only | If W60=1 go to W61  If W60=0,8 go to W63 |
| W61 | How long did s/he have paralysis of the lower limbs? (in days) | Number pad  Don’t remember (888) | Please use the number pad | Go to W62 |
| W62 | Did the paralysis of the lower limbs start suddenly, quickly within a single day, or slowly over many days? | Suddenly (1)  Fast (in a day) (2)  Slowly (many days) (3)  Don’t remember (8) | Please press on one box only | Go to W63 |
| W63 | Was there any change in the amount of urine s/he passed daily? | Yes (1)  No (0)  Don’t remember (8) | Please press on one box only | If W63=1 go to W64  If W63=0,8 go to W66 |
| W64 | For how long did s/he have the change in the amount of urine s/he passed daily? (in days) | Number pad  Don’t remember (888) | Please use the number pad | Go to W65 |
| W65 | How much urine did s/he pass? | Too much (1)  Too little (2)  No urine at all (3)  Don’t remember (8) | Please press on one box only | Go to W66 |
| W66 | During the illness that led to death, did s/he have any skin rash? | Yes (1)  No (0)  Don’t remember (8) | Please press on one box only | If W66=1 go to W67  If W66=0,8 go to W70 |
| W67 | For how long did s/he have the skin rash? (in days) | Number pad  Don’t remember (888) | Please use the number pad | Go to W68a |
| Intro W68a | Was the rash located on: |  |  |  |
| W68a | The face? | Yes (1)  No (0)  Don’t remember (8) | Please press on one box only | Go to W68b |
| W68b | The trunk? | Yes (1)  No (0)  Don’t remember (8) | Please press on one box only | Go to W68c |
| W68c | On the arms and legs? | Yes (1)  No (0)  Don’t remember (8) | Please press on one box only | Go to W69 |
| W69 | What did the rash look like? | Measles rash (1)  Rash with clear fluid (2)  Rash with pus (3)  Don’t remember (8) | Please press on one box only | Go to W70 |
| W70 | Did s/he have red eyes? | Yes (1)  No (0)  Don’t remember (8) | Please press on one box only | Go to W71 |
| W71 | Did s/he have bleeding from the nose, mouth, or anus? | Yes (1)  No (0)  Don’t remember (8) | Please press on one box only | Go to W72 |
| W72 | Did s/he have weight loss? | Yes (1)  No (0)  Don’t remember (8) | Please press on one box only | If W72=1 go to W73  If W72=0,8 go to W75 |
| W73 | For how long before death did s/he have the weight loss? (in days) | Number pad  Don’t remember (888) | Please use the number pad | Go to W74 |
| W74 | Did s/he look very thin and wasted? | Yes (1)  No (0)  Don’t remember (8) | Please press on one box only | Go to W75 |
| W75 | Did s/he have mouth sores or white patches in the mouth or on the tongue? | Yes (1)  No (0)  Don’t remember (8) | Please press on one box only | If W75=1 go to W76  If W75=0,8 go to W77 |
| W76 | For how long did s/he have mouth sores or white patches in the mouth or on the tongue? (in days) | Number pad  Don’t remember (888) | Please use the number pad | Go to W77 |
| W77 | Did s/he have any swelling? | Yes (1)  No (0)  Don’t remember (8) | Please press on one box only | If W77=1 go to W78  If W77=0,8 go to W80 |
| W78 | For how long did s/he have the swelling? (in days) | Number pad  Don’t remember (888) | Please use the number pad | Go to W79a |
| Intro W79a | Was the swelling on: |  |  |  |
| W79a | The face? | Yes (1)  No (0)  Don’t remember (8) | Please press on one box only | Go to W79b |
| W79b | The joints? | Yes (1)  No (0)  Don’t remember (8) | Please press on one box only | Go to W79c |
| W79c | The ankles? | Yes (1)  No (0)  Don’t remember (8) | Please press on one box only | Go to W79d |
| W79d | The whole body? | Yes (1)  No (0)  Don’t remember (8) | Please press on one box only | Go to W79e |
| W79e | Any other place? | Yes (1)  No (0)  Don’t remember (8) | Please press on one box only | Go to W80 |
| W80 | Did s/he have any lumps? | Yes (1)  No (0)  Don’t remember (8) | Please press on one box only | If W80=1 go to W81  If W80=0,8 go to W83 |
| W81 | For how long did s/he have the lumps? (in days) | Number pad  Don’t remember (888) | Please use the number pad | Go to W82a |
| Intro W82a | Were the lumps on: |  |  |  |
| W82a | The neck? | Yes (1)  No (0)  Don’t remember (8) | Please press on one box only | Go to W82b |
| W82b | The armpit? | Yes (1)  No (0)  Don’t remember (8) | Please press on one box only | Go to W82c |
| W82c | The groin? | Yes (1)  No (0)  Don’t remember (8) | Please press on one box only | Go to W82d |
| W82d | Any other place? | Yes (1)  No (0)  Don’t remember (8) | Please press on one box only | Go to W83 |
| W83 | Did s/he have yellow discoloration of the eyes? | Yes (1)  No (0)  Don’t remember (8) | Please press on one box only | If W83=1 go to W84  If W83=0,8 go to W85 |
| W84 | For how long did s/he have the yellow discoloration of the eyes? (in days) | Number pad  Don’t remember (888) | Please use the number pad | Go to W85 |
| W85 | Did her/his hair color change to reddish or yellowish? | Yes (1)  No (0)  Don’t remember (8) | Please press on one box only | If W85=1 go to W86  If W85=0,8 go to W87 |
| W86 | For how long did s/he have reddish/yellowish hair? (in days) | Number pad  Don’t remember (888) | Please use the number pad | Go to W87 |
| W87 | Did s/he look pale (thinning/lack of blood) or have pale palms, eyes or nail beds? | Yes (1)  No (0)  Don’t remember (8) | Please press on one box only | If W87=1 go to W88  If W87=0,8 go to W89 |
| W88 | For how long did s/he look pale (thinning/lack of blood) or have pale palms, eyes, or nail beds? (in days) | Number pad  Don’t remember (888) | Please use the number pad | Go to W89 |
| W89 | Did s/he have sunken eyes? | Yes (1)  No (0)  Don’t remember (8) | Please press on one box only | If W89=1 go to W90  If W89=0,8 go to W91 |
| W90 | For how long did s/he have sunken eyes? (in days) | Number pad  Don’t remember (888) | Please use the number pad | Go to W91 |
| Intro W91 | Now I will ask you some questions about the health services you asked for your baby’s final illness. |  |  |  |
| W91 | Was s/he vaccinated for measles? | Yes (1)  No (0)  Don’t remember (8) | Please press on one box only | Go to W92 |
| W92 | Did s/he receive any treatment for the illness that led to death? | Yes (1)  No (0)  Don’t remember (8) | Please press on one box only | If W92=1 go to W93a  If W92=0,8 go to W96 |
| Intro W93a | What type of treatment did s/he receive: |  |  |  |
| W93a | Oral rehydration salts and/or intravenous fluids (drip) treatment? | Yes (1)  No (0)  Don’t remember (8) | Please press on one box only | Go to W93b |
| W93b | Blood transfusion? | Yes (1)  No (0)  Don’t remember (8) | Please press on one box only | Go to W93c |
| W93c | Treatment/food through a tube passed through the nose? | Yes (1)  No (0)  Don’t remember (8) | Please press on one box only | Go to W93d |
| W93d | Any other treatment? | Yes (1)  No (0)  Don’t remember (8) | Please press on one box only | Go to W94a |
| Intro W94a | Please tell me at which of the following places/facilities s/he received treatment during the illness that led to death: |  |  |  |
| W94a | Home? | Yes (1)  No (0)  Don’t remember (8) | Please press on one box only | Go to W94b |
| W94b | Traditional healer? | Yes (1)  No (0)  Don’t remember (8) | Please press on one box only | Go to W94c |
| W94c | Government clinic? | Yes (1)  No (0)  Don’t remember (8) | Please press on one box only | Go to W94d |
| W94d | Government hospital? | Yes (1)  No (0)  Don’t remember (8) | Please press on one box only | Go to W94e |
| W94e | Private clinic? | Yes (1)  No (0)  Don’t remember (8) | Please press on one box only | Go to W94f |
| W94f | Private hospital? | Yes (1)  No (0)  Don’t remember (8) | Please press on one box only | Go to W94g |
| W94g | Pharmacy, drug seller, store? | Yes (1)  No (0)  Don’t remember (8) | Please press on one box only | Go to W94h |
| W94h | Any other place or facility? | Yes(1)  No (0)  Don’t remember (8) | Please press on one box only | Go to W95 |
| W95 | In the month before death, how many contacts with formal health services did s/he have? | Number pad  Don’t remember (888) | Please use the number pad | Go to W96 |
| W96 | Did a health care worker tell you the cause of death? | Yes (1)  No (0)  Don’t remember (8) | Please press on one box only | Go to W97 |
| W97 | Did s/he have any operation for the illness? | Yes (1)  No (0)  Don’t remember (8) | Please press on one box only | If W97=1 go to W98  If W97=0,8 go to W100 |
| W98 | How long before death did s/he have the operation? (in days) | Number pad  Don’t remember (888) | Please use the number pad | Go to W99 |
| W99 | On what part of the body was the operation? | Abdomen (1)  Chest (2)  Head (3)  Other (4)  Don’t remember (8) | Please press on one box only | Go to W100 |
| W100 | Do you have a death certificate for the baby? | Yes (1)  No (0) | Please press on one box only | If W100=1 go to W101  If W100=0,8 SAVE INTERVIEW AND FILL IN THE PINK COLOR PAPER. |
| W101 | Can I see the death certificate? | Yes (1)  No (0) | Please press on one box only | SAVE INTERVIEW AND FILL IN THE PINK COLOR PAPER. |
