# Supplementary material for: Evaluating the Impact of Zimbabwe’s Prevention of Mother-to-Child HIV Transmission Program: Population-Level Estimates of HIV-Free Infant Survival Pre-Option A
Source: PLoS One. 2015 Aug 6;10(8):e0134571. doi: 10.1371/journal.pone.0134571 (PMC4527770; doi:10.1371/journal.pone.0134571)
Supplement: S2 File — (DOCX) [file pone.0134571.s003.docx]

**FACILITY-BASED QUESTIONNAIRE**

**PMTCT BASELINE SURVEY**

***To the Interviewer:***

***Conduct this interview with the Sister in Charge at this facility.***

Date: ________________________ Interviewer: ___________________________________

Person(s) Interviewed from the Health Facility (Name, Title, Phone number):

_____________________________ ___________________________ ____________________

_____________________________ ___________________________ ____________________

Name of Facility: __________________________________ Facility code: ____________

District: ____________________________ Province: _______________________________

| **#** | **Question** | **Answers** | **Skip** |
| --- | --- | --- | --- |
| **1** | **What type of facility is this?** | Central hospital.............................1  Provincial hospital..........................2  District hospital............................3  Mission hospital.............................4  Rural health hospital........................5  Local Authority..............................6  Private hospital.............................7  Rural health clinic..........................8  Polyclinic...................................9  Satellite clinic............................10  Other (specify)_____________________________11 |  |
| **2** | **What is the estimated population living in this facility’s catchment area?** | Number of people__________________  Don’t know (999) |  |
| **3** | **How many rooms are in this entire facility?** | Total number of rooms___________  Don’t know (999) |  |
| **4** | **How many staff members does this facility have on payroll (excluding village health workers/ health promoters)?** | Number_______________  Don’t know (999) |  |
| **5** | **How many village health workers/ health promoters were on this facility’s payroll on the last paycheck?** | Number_______________  Don’t know (999) |  |

| **#** | **Question** | | **Answers** | **Skip** |
| --- | --- | --- | --- | --- |
| **6** | **Does this facility provide pregnant women with any services for the prevention of mother-to-child transmission (PMTCT) of HIV?** This includes (but is not limited to) HIV testing during antenatal care, prophylactic options such as sdNVP, AZT, and HAART. | | Yes..........................................1  No...........................................0  Don’t know...................................9 | **Q7 STOP**  **Q7** |
| **7** | **How many days in a typical week is this facility open for ANC services?** | | 1 day........................................1  2 days.......................................2  3 days.......................................3  4 days.......................................4  5 days.......................................5  6 days.......................................6  7 days.......................................7  Don’t know...................................8 |  |
|  | **Now I will ask you about various PMTCT services your facility may be offering.** | | |  |
| **8** | **In the past month, did this facility offer antenatal care or did it make referrals to other facilities?** | Offered at this facility.....................1  Pregnant women referred to another facility..2  Both.........................................3  Neither......................................4 | |  |
| **9** | **In the past month, did this facility offer HIV testing for pregnant women or did it make referrals to other facilities?** | Offered at this facility.....................1  Pregnant women referred to another facility..2  Both.........................................3  Neither......................................4 | | **Q10**  **Q13**  **Q10**  **Q13** |
| **10** | **When does this facility offer HIV testing to pregnant women?**  Instruction: Check ALL that apply. | During ANC...................................A  During labour................................B  Postnatally..................................C | |  |
| **11** | **Does this facility offer repeat HIV testing to HIV-negative pregnant women?** For example, if a woman tests HIV negative during ANC would you offer her another HIV test during delivery? | Yes..........................................1  No...........................................0  Don’t know...................................9 | |  |

| **#** | **Question** | **Answers** | **Skip** |
| --- | --- | --- | --- |
| **12** | **Is HIV testing part of the standard procedures for antenatal care or is it a procedure women may choose to undergo?** | Part of standard antenatal procedures (opt-out).........................................1  Women can choose to test for HIV (opt-in)..........................................2  Don’t know...................................9 |  |
| **13** | **In the past month, did this facility offer maternal ARV prophylaxis or did it make referrals to other facilities?** | Offered at this facility.....................1  Pregnant women referred to another facility..2  Both.........................................3  Neither......................................4 | **Q15**  **Q14**  **Q14**  **Q17** |
| **14** | **Compared to this facility, how far is the nearest facility that offers maternal ARV prophylaxis?** | Kilometers _____________  Don’t know (999) | **If Q13=2**  **->Q17** |
| **15** | **In the past month, what type of maternal ARV prophylaxis was provided at this facility?** Instruction: DO NOT READ OPTIONS. | SdNVP prophylaxis only.......................1  MER from 28 weeks gestation..................2  MER from 14 weeks gestation..................3  Don’t know...................................9 | **Q16**  **Q16**  **Q16**  **Q17** |
| **16** | **When did the facility begin offering this regimen of maternal ARV prophylaxis?** | Month/Year________/________  Don’t know (999) |  |
| **17** | **In the past month, did this facility offer infant ARV prophylaxis** **or did it make referrals to other facilities?** | Offered at this facility.....................1  Infants referred to another facility.........2  Both.........................................3  Neither......................................4 | **Q19**  **Q18**  **Q18**  **Q21** |
| **18** | **In reference to this facility, how far is the nearest facility that offers infant ARV prophylaxis?** | Kilometers _____________  Don’t know (999) | **If Q17=2 ->Q21** |
| **19** | **In the past month, what type of infant ARV prophylaxis was provided in this facility?**  Instruction: DO NOT READ OPTIONS. | SdNVP only...................................1  SdNVP plus AZT (7 days/28days)...............2  NVP throughout duration of breastfeeding.....3  Don’t know...................................9 | **Q20**  **Q20**  **Q20**  **Q21** |

| **#** | **Question** | **Answers** | **Skip** |
| --- | --- | --- | --- |
| **20** | **When did the facility begin offering this regimen of infant ARV prophylaxis?** | Month/Year________/________  Don’t know (999) |  |
| **21** | **In the past month, when a pregnant or breastfeeding woman was identified as needing to be initiated on ART, where would she go?**  Instruction: Check ALL that apply. | Referred to the dispensary in this facility..A  ART initiated in Maternal and Child Health...B  Referred to off-site ART clinic..............C  Initiated during mobile outreach services....D | **Q22**  **Q22**  **Q23**  **Q23** |
| **22** | **When did the facility begin initiating pregnant women on ART?** | Month/Year________/________  Don’t know (999) |  |
| **23** | **In the past month, how did women access CD4 testing at this facility?** | CD4 testing done in the laboratory in the same facility.....................................1  CD4 test samples collected at this facility are sent to an off-site laboratory...........2  Women referred for CD4 testing to an off-site facility.....................................3  CD4 testing done in Maternal Child Health using POC CD4 machine........................4 | **Q25**  **Q24**  **Q24**  **Q25** |
| **24** | **How far from this facility is the nearest facility with a CD4 testing machine?** | Kilometers _____________  Don’t know (999) | **Q26**  **Q26** |
| **25** | **When did this facility begin offering CD4 testing?** | Month/Year________/________  Don’t know (999) |  |
| **26** | **On average, how many days are needed between specimen collection and availability of CD4 test results?** | Days ___________  The same day (0)  Don’t know (999) |  |
| **27** | **In the past month, who served as the link between this facility and the community for PMTCT follow up?**  Instruction: Check ALL that apply. | There is no link with the community..........A  The community health nurse...................B  Village health workers.......................C  Home based care cadre........................D  Outreach teams...............................E  Environmental health officers................F  Others (specify)_____________________________G |  |

| **#** | **Question** | **Answers** | **Skip** |
| --- | --- | --- | --- |
| **28** | **In the past month, did this facility offer labour and delivery services** **or did it make referrals to other facilities?** | Offered at this facility.....................1  Women referred to another facility...........2  Both.........................................3  Neither......................................4 |  |
| **29** | **In the past month, did this facility offer** **postnatal check-ups or did it make referrals to other facilities?** | Offered at this facility.....................1  Women referred to another facility...........2  Both.........................................3  Neither......................................4 |  |
| **30** | **In the past month, did this facility offer** **immunization visits or did it make referrals to other facilities?** | Offered at this facility.....................1  Women referred to another facility...........2  Both.........................................3  Neither......................................4 |  |
| **31** | **In the past month, if a mother wanted to have her child tested for HIV (early infant diagnosis), how would that request be managed?** | EID testing done in the laboratory at this facility.....................................1  EID samples sent to an off-site laboratory...2  Infants referred for EID testing at an off-site facility................................3 | **Q33**  **Q32**  **Q32** |
| **32** | **How far away from this facility is the nearest facility with EID testing capabilities?** | Kilometers _____________  Don’t know (999) | **Q34**  **Q34** |
| **33** | **When did the facility begin offering EID testing?** | Month/Year________/________  Don’t know (999) |  |
| **34** | **On average, between specimen collection and receipt of results, how many days does it take to receive EID test results?** | Days ___________  The same day (0)  Don’t know (999) |  |
| **35** | **In the past month, was Cotrimoxazole prophylaxis available at this facility for the following patients?**  Instruction: Check ALL that apply. | HIV+ pregnant women..........................A  HIV infected children........................B  HIV exposed infants..........................C  None of the above............................D | **Q36**  **Q36**  **Q36**  **Q37** |

| **#** | **Question** | **Answers** | **Skip** |
| --- | --- | --- | --- |
| **36** | **When did the facility begin offering Cotrimoxazole prophylaxis?** | Month/Year________/________  Don’t know (999) |  |
| **37** | **In the past month, did this facility charge for ANC services (including PMTCT services)?** | Yes..........................................1  No...........................................0  Don’t know...................................9 | **Q38**  **Q42**  **Q42** |
| **38** | **In this facility, are women offered a discount if they pre-pay or pre-register for all ANC services (including PMTCT)?** | Yes..........................................1  No...........................................0  Don’t know...................................9 | **Q39**  **Q40**  **Q40** |
| **39** | **What is the discount for pre-paying or pre-registering for ANC?** | Amount________ OR  Percentage of total cost________  Don’t know (999) |  |
| **40** | **Does this facility charge one inclusive price for ANC services (including PMTCT) or separately for each ANC service?** | Inclusive....................................1  Separate.....................................2  Don’t know...................................9 | **Q41A**  **Q41B**  **Q41B** |
| **41** | **How much is each mother charged for the following services today?** |  |  |
| **41A** | *Inclusive price for all ANC/PMTCT services* | USD_________ OR  Rand________  Not applicable (service not offered) (999) | **Q41L**  **Q41L**  **Q41A** |
| **41B** | *An antenatal consultation* | USD_________ OR  Rand________  Not applicable (service not offered) (999) |  |
| **41C** | *An HIV test for a pregnant woman* | USD_________ OR  Rand________  Not applicable (service not offered) (999) |  |
| **41D** | *A CD4 count test* | USD_________ OR  Rand________  Not applicable (service not offered) (999) |  |
| **41E** | *Maternal ARV prophylaxis (per month)* | USD_________ OR  Rand________  Not applicable (service not offered) (999) |  |
| **41F** | *ART for a pregnant woman (per month)* | USD_________ OR  Rand________  Not applicable (service not offered) (999) |  |
| **41G** | *Labor & delivery* | USD_________ OR  Rand________  Not applicable (service not offered) (999) |  |

| **#** | **Question** | **Answers** | **Skip** |
| --- | --- | --- | --- |
| **41H** | *Single dose NVP for a pregnant woman* | USD_________ OR  Rand________  Not applicable (service not offered) (999) |  |
| **41I** | *Single dose NVP for an infant* | USD_________ OR  Rand________  Not applicable (service not offered) (999) |  |
| **41J** | *An immunization visit* | USD_________ OR  Rand________  Not applicable (service not offered) (999) |  |
| **41K** | *A postnatal check-up* | USD_________ OR  Rand________  Not applicable (service not offered) (999) |  |
| **41L** | *CTX for the mother (per month)* | USD_________ OR  Rand________  Not applicable (service not offered) (999) |  |
| **41M** | *CTX for the infant (per month)* | USD_________ OR  Rand________  Not applicable (service not offered) (999) |  |
| **41N** | *NVP for the infant during breastfeeding (per month)* | USD_________ OR  Rand________  Not applicable (service not offered) (999) |  |
| **41P** | *An HIV test for an infant* | USD_________ OR  Rand________  Not applicable (service not offered) (999) |  |
| **41R** | *ARV therapy for a child (per month)* | USD_________ OR  Rand________  Not applicable (service not offered) (999) |  |
| **42** | **In the last month, have stock-outs affected the provision of any of the following PMTCT services?**  Instruction: Check ALL that apply. | Maternal ARV prophylaxis.....................A  Infant ARV prophylaxis.......................B  CTX for the mother...........................C  CTX for the infant...........................D  ARV therapy for a child......................E  ART therapy for a pregnant woman.............F  None of the above............................G |  |
| **43** | **Have staff members been trained on providing MER from 14 weeks gestation (WHO 2010 guidelines)?** | Yes..........................................1  No...........................................0  Don’t know...................................9 | **Q44**  **Q45**  **Q45** |
| **44** | **How many staff members have been trained on providing MER from 14 weeks gestation (WHO 2010 guidelines)?** | Number_____________ AND  Percentage of all staff_________  Don’t know (999) |  |
| **45** | **Does the facility currently provide MER from 14 weeks gestation (WHO 2010 guidelines)?** | Yes..........................................1  No...........................................0  Don’t know...................................9 | **Q46**  **END**  **END** |
| **46** | **When did the facility begin using MER from 14 weeks gestation (WHO 2010 guidelines)?** | Month/Year________/________  Don’t know (999) |  |

**COMMENTS:**

***Instruction*: Record any comments you may have based on the information you were provided. WRITE LEGIBLY.**
