# Supplementary material for: Evaluating the Impact of Zimbabwe’s Prevention of Mother-to-Child HIV Transmission Program: Population-Level Estimates of HIV-Free Infant Survival Pre-Option A
Source: PLoS One. 2015 Aug 6;10(8):e0134571. doi: 10.1371/journal.pone.0134571 (PMC4527770; doi:10.1371/journal.pone.0134571)
Supplement: S3 File — (DOCX) [file pone.0134571.s004.docx]

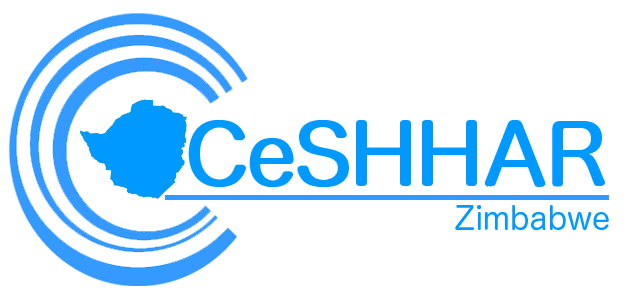


**The Centre for Sexual Health and HIV AIDS Research Zimbabwe (CeSHHAR Zimbabwe)**

**21 Rowland Square, Milton Park**

**Harare, Zimbabwe**

**Phone +263 772 257949**

21 Rowland Square, Milton Park,

Harare, Zimbabwe

**CONSENT TO PARTICIPATE IN A RESEARCH STUDY**

***Evaluation of Zimbabwe’s Accelerated***

***Prevention of Mother-to-Child Transmission of HIV Program***

**Principal Investigator:** Dr. Frances Cowan, MD

**What you should know about this research study:**

- We give you this consent so that you may read about the purpose, risks, and benefits of this research study.
- Routine care is based upon the best-known treatment and is provided with the main goal of helping the individual patient. The main goal of research studies is to gain knowledge that may help future patients.
- We cannot promise that this research will benefit you or your child. Just like regular care, this research can have side effects that can be serious or minor.
- You have the right to refuse to take part or refuse to allow your child to take part, or agree for you or your child to take part now and change your mind later.
- Whatever you decide, it will not affect your or your child’s regular care.
- Please review this consent form carefully. Ask any questions before you make a decision.
- Your participation and your choice to allow your child to participate is voluntary.

**Introduction**

I am a *data capturer* with CeSHHAR Zimbabwe group based in Harare. We are conducting a research study with the Ministry of Health and Child Welfare and the University of California, Berkeley to learn about a program to protect children in Zimbabwe from HIV infection. This study was reviewed and approved by the Medical Research Council of Zimbabwe. You and your child are invited to participate in this study because you live in Zimbabwe and are the mother or caregiver of a child who is or who would have been between 9 and 18 months of age.

This form tells you why the study is being done, who is being asked to participate, and what your participation will involve. Please read this form carefully. If you would prefer, I can read the form to you. Afterwards, I will review a few key points with you and answer any questions you might have. If you are interested in participating in the study after reviewing this form, I will ask for your signature.

**Purpose**

This study is being done to learn more about how to protect children in Zimbabwe from HIV infection. Sometimes babies get HIV infection from their mothers, and Zimbabwe has a special program to help prevent this from happening. The study will see if the program is working to prevent HIV infection in babies and to help children in Zimbabwe live longer.

This study is being conducted in Harare, Mashonaland West, Mashonaland Central, Manicaland, and Matabeleland South provinces in Zimbabwe. Households within these provinces were selected by a random lottery.

**Procedures and Duration**

If you agree to participate in this study, you will be asked to do the following:

- Complete a short survey that should take about 30 minutes to complete. You can identify a quiet and private place where we can complete the survey. The survey will ask questions about you and your baby including your use of health care, your pregnancy and birth history (only if you are the biological mother), your HIV testing history and status, and basic characteristics of your household. If your baby passed away, we will ask you about the possible causes of his/ her death.
- Show your maternal health card and your child’s health card, if still in your possession. This will allow us to collect accurate information about the medications you and your child received during your pregnancy, delivery, and after delivery.
- Provide a blood sample (only if you are the biological mother). The survey assistant will clean your finger with a swab of alcohol, allow the cleaned area to dry, prick your finger with a small needle to produce small drops of blood, and collect a few blood drops on a piece of filter paper.

If your child is present and you consent to your child’s participation in the study, his/her involvement will include the collection of a blood spot sample. This will include: cleaning the child’s heel with a swab of alcohol, allowing the cleaned area to dry, pricking the child’s heel with a small needle to produce small drops of blood that will be collected on a piece of filter paper.

Once collected, the filter paper(s) containing your and/or your child’s blood will be dried and then sent to the National Laboratory for HIV testing. The Laboratory will destroy the blood samples according to standard biohazard procedures once the study is finished (in 2015).

Neither your child’s name nor your name will be associated with the blood sample provided by you or your child; instead it will be identified with a random code. We will give you a card with a random code that matches your blood sample. You will be able to pick up the test results at your local clinic in minimum 4 weeks, as long as you show this card to the nurse. Your results will be at the clinic for 3 months, so if you go to the clinic more than four months from now, you will no longer be able to receive your results. Your HIV test results will be sent to the clinic in a sealed envelope identified by the same random code. If you do not want to receive this card with the code that matches your and your child’s blood samples, we will not give it to you and will not send your results to the local clinic. Choosing not to take the card will in no way impact your participation in the study.

Study timing: The questionnaire should take minimum 30 minutes to complete. The collection of one dried blood spot sample should take no more than 10 minutes. The total participation time should be less than an hour.

**Risks and Discomforts**

Some of the questions asked in the questionnaire may make you feel uncomfortable or embarrassed. Some questions may ask about children you may have had that have passed away, and these questions may cause you some sadness or discomfort. You are free to decline to answer any question or stop participating at any time.

Your child may experience brief discomfort from the heel stick during the collection of the blood spots. Infections at the site of the heel stick are rare. Similarly, while the finger stick blood sample you provide might be uncomfortable, it is very unlikely to cause an infection. If you are currently pregnant, there is no increased risk to you from this procedure. We will not know the result of the dried blood spot tests immediately because they require analysis in the national medical laboratory.

**Benefits**: There is no direct benefit to you or your child for participating in the study. However, this study will help the study team understand if a program in Zimbabwe to prevent HIV infection in babies is working. This is very important so that we know if the program is having the intended effect, or if there are ways that we can help to make the program work better.

**Compensation/Payment**: We will provide you with a small gift as a thank you for your time. This will be in the form of a small household item valued at US$5 or below.

**Confidentiality**

If you agree to participate in this study, we will ask you to sign and to provide your and your child’s names on this form. However, your and your child’s names will **not** be recorded on any other survey materials; instead these materials will be linked using a unique code. More specifically, we will **not** enter your name into the Personal Digital Assistant we will use to conduct the questionnaire, on the filter paper on which we will collect your and/or your child’s blood, or on any other forms. No one will be able to link your questionnaire or the blood samples to this form which has your name on it. Once we leave your house, even if you wanted us to, we would be unable to identify **your** questionnaire or blood samples among the thousands of questionnaires and blood samples collected in this study. This guarantees that no one will know your answers to the questionnaire and your or your child’s HIV status based on the blood samples collected in this study. However, we will give you a card that has the same code as your blood sample, unless you tell us you don’t want the card. You will then be able to use that card to pick up your and/ or your child’s test results at the local clinic in minimum 4 weeks time. Publications or presentations with results of this study will not have access to individual names or other personally identifiable information.

All paper documents will be kept in a locked cabinet in a locked office, and only authorized study personnel will have access to these records. The study database will be password protected so that only authorized study personnel have access to these records.

When the study is completed, the data maybe saved for use in future research. The study team will retain this study information for up to ten years after the study is over. The same measures described above will be taken to protect confidentiality of this study data.

**Costs of Study Participation**: There is no cost to you for participating in this study.

**Rights***: Participation in this study is completely voluntary.* You have the right to decline to participate or to withdraw from the study without penalty. Similarly, you can withdraw your child’s participation at anytime.

**Questions**: Before you sign this form, please ask any questions on any aspect of this study that is unclear to you. You may take as much time as necessary to think it over.

If you have any additional questions or concerns about this study, you can contact Dr. Frances Cowan at 04-707 289 or the Study Coordinator Constancia Watadzaushe at 0772 288 163

If you have any questions or concerns about your rights and treatment as a research subject, you may contact Medical Research Council of Zimbabwe at 04-791792 or 791193*.*

**CONSENT TO PARTICIPATE IN A RESEARCH STUDY**

***Evaluation of Zimbabwe’s Accelerated***

***Prevention of Mother-to-Child Transmission of HIV Program***

**Authorization for your participation**: You are making a decision whether or not to participate in this study. Your signature indicates that you have read and understood the information provided above, have had all your questions answered, and have decided to participate.

Do you agree to participate in the questionnaire survey? Yes_____ No _____

Do you agree to provide a blood sample? Yes_____ No _____ NA_____

I am going to give you a numbered card that matches your HIV test result. If you do NOT want to receive this card, please indicate that here: I do NOT want to receive the card_____

**Authorization for your child’s participation:** You are making a decision whether or not to allow your child to participate in this study. Your signature indicates that you have read and understood the information provided above, have had all your questions answered, and have decided to allow your child to participate. The date you sign this document to enroll your child in this study, that is, today’s date, MUST fall between the dates indicated on the approval stamp affixed to each page. These dates indicate that this form is valid when you enroll your child in the study but do not reflect how long your child may participate in the study. Each page of this Informed Consent Form is stamped to indicate the form’s validity as approved by MRCZ.

Do you agree that your child participate in this study by providing a blood sample?

Yes_____ No _____ NA_____

I am going to give you a numbered card that matches your child’s HIV test result. If you do NOT want to receive this card, please indicate that here: I do NOT want to receive the card_____

Participant's Name *(please print)*

_______________

Participant's Signature Date

___________________________________

*Infant Participant Name (*please print*)

­­­­­­­­­________________________________ _______________

Witness’ Name *(please print)* Date

____________________________________

Witness’ Relationship to mother/caregiver

_______________

Interviewer’s Name Date

**We will give you a copy of this consent form to keep for your records.**

**CONSENT TO PARTICIPATE IN A RESEARCH STUDY**

***Evaluation of Zimbabwe’s Accelerated***

***Prevention of Mother-to-Child Transmission of HIV Program***

**Authorization for your participation**: You are making a decision whether or not to participate in this study. Your signature indicates that you have read and understood the information provided above, have had all your questions answered, and have decided to participate.

Do you agree to participate in the questionnaire survey? Yes_____ No _____

Do you agree to provide a blood sample? Yes_____ No _____ NA_____

I am going to give you a numbered card that matches your HIV test result. If you do NOT want to receive this card, please indicate that here: I do NOT want to receive the card_____

**Authorization for your child’s participation:** You are making a decision whether or not to allow your child to participate in this study. Your signature indicates that you have read and understood the information provided above, have had all your questions answered, and have decided to allow your child to participate. The date you sign this document to enroll your child in this study, that is, today’s date, MUST fall between the dates indicated on the approval stamp affixed to each page. These dates indicate that this form is valid when you enroll your child in the study but do not reflect how long your child may participate in the study. Each page of this Informed Consent Form is stamped to indicate the form’s validity as approved by MRCZ.

Do you agree that your child participate in this study by providing a blood sample?

Yes_____ No _____ NA_____

I am going to give you a numbered card that matches your child’s HIV test result. If you do NOT want to receive this card, please indicate that here: I do NOT want to receive the card_____

Participant's Name *(please print)*

_______________

Participant's Signature Date

___________________________________

*Infant Participant Name (*please print*)

­­­­­­­­­________________________________ _______________

Witness’ Name *(please print)* Date

____________________________________

Witness’ Relationship to mother/caregiver

_______________

Interviewer’s Name Date

**We will give you a copy of this consent form to keep for your records.**
