# Supplementary material for: Evaluating the Impact of Zimbabwe’s Prevention of Mother-to-Child HIV Transmission Program: Population-Level Estimates of HIV-Free Infant Survival Pre-Option A
Source: PLoS One. 2015 Aug 6;10(8):e0134571. doi: 10.1371/journal.pone.0134571 (PMC4527770; doi:10.1371/journal.pone.0134571)
Supplement: S4 File — (DOCX) [file pone.0134571.s005.docx]

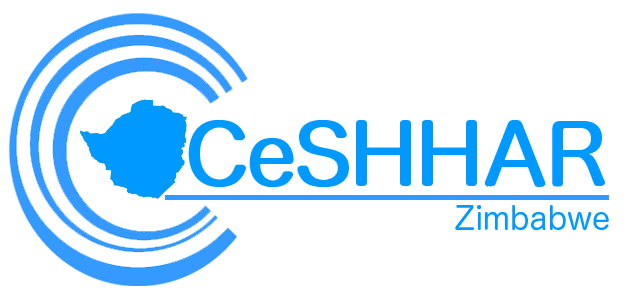


**The Centre for Sexual Health and HIV AIDS Research Zimbabwe (CeSHHAR Zimbabwe)**

**21 Rowland Square, Milton Park**

**Harare, Zimbabwe**

**Phone +263 772 257949**

21 Rowland Square, Milton Park,

Harare, Zimbabwe

**CONSENT TO PARTICIPATE IN THE FACILITY QUESTIONNAIRE**

***Evaluation of Zimbabwe’s Accelerated***

***Prevention of Mother-to-Child Transmission of HIV Program***

**Principal Investigator:** Dr. Frances Cowan, MD

**What you should know about this research study:**

- We give you this consent so that you may read about the purpose, risks, and benefits of this research study.
- Routine care is based upon the best-known treatment and is provided with the main goal of helping the individual patient. The main goal of research studies is to gain knowledge that may help future patients.
- We cannot promise that this research will benefit you. Just like regular care, this research can have side effects that can be serious or minor.
- You have the right to refuse or agree to take part now and change your mind later.
- Whatever you decide, it will not affect your regular care.
- Please review this consent form carefully. Ask any questions before you make a decision.
- Your participation is voluntary.

**Introduction**

I am a *team leader* with CeSHHAR Zimbabwe group based in Harare. We are conducting a research study with the Ministry of Health and Child Welfare and the University of California, Berkeley to learn about a program to protect children in Zimbabwe from HIV infection. *This study was reviewed and approved by the Medical Research Council of Zimbabwe and is supported by the Ministry of Health and Child Welfare.*

This form tells you why the study is being done, who is being asked to participate, and what your participation will involve. Please read this form carefully. If you would prefer, I can read the form to you. Afterwards, I will review a few key points with you and answer any questions you might have. If you are interested in participating in the study after reviewing this form, I will ask for your signature.

**Purpose**

This study is being done to learn more about how to protect children in Zimbabwe from HIV infection. Sometimes babies get HIV infection from their mothers, and Zimbabwe has a special program to help prevent this from happening. The study will see if the program is working to prevent HIV infection in babies and to help children in Zimbabwe live longer.

This study is being conducted in Harare, Mashonaland West, Mashonaland Central, Manicaland, and Matabeleland South provinces in Zimbabwe. Health facilities within these provinces were selected by a random lottery around which the household survey will be conducted.

**Procedures and Duration**

If this health facility agrees to participate in this study, you will be asked to complete a short survey that should take about 30 minutes to complete. The survey will collect information about the health facility and the antenatal care and prevention of mother-to-child of HIV services provided.

**Risks and Discomforts**

There are no risks or discomfort from your participation on behalf of your health facility in this survey. If you decide to take part in this research, your participation will have no effect on the personnel, activities or funding of the facility.

**Benefits**

There is no direct benefit to you or your health facility for participating in the study. However, this survey is an important piece in helping the study team understand if a program in Zimbabwe to prevent HIV infection in babies is working. This is very important so that we know if the program is having the intended effect, or if there are ways that we can help to make the program work better.

**Compensation/Payment**

There is no compensation for participating in this survey.

**Confidentiality**

All paper documents will be kept in a locked cabinet in a locked office, and only authorized study personnel will have access to these records. The study database will be password protected so that only authorized study personnel have access to these records.

When the study is completed, the data maybe saved for use in future research. The study team will retain this study information for up to ten years after the study is over. The same measures described above will be taken to protect confidentiality of this study data.

**Costs of Study Participation**

There is no cost to you for participating in this study.

**Rights**

*Participation in this study is completely voluntary.* You have the right to decline to participate or to withdraw from the study without penalty. If you have any questions at any time during the survey (for example, the meaning of a certain abbreviation), please ask. *Feel free to consult with any colleague in case any question is too specific.*

**Questions**

Before you sign this form, please ask any questions on any aspect of this study that is unclear to you. You may take as much time as necessary to think it over.

If you have any additional questions or concerns about this study, you can contact Dr. Frances Cowan at 04-707 289 or the Study Coordinator Constancia Watadzaushe at 0772 288 163.

**CONSENT TO PARTICIPATE IN A RESEARCH STUDY**

***Evaluation of Zimbabwe’s Accelerated***

***Prevention of Mother-to-Child Transmission of HIV Program***

**Authorization for the participation of this health facility**: You are making a decision whether or not to participate in this study on behalf of this health facility. Your signature indicates that you have read and understood the information provided above, have had all your questions answered, and have decided to participate.

Name of Health Facility

Health Facility Representative’s Name *(please print)*

_______________

Health Facility Representative’s Signature Date

_______________

Interviewer’s Name Date

**CONSENT TO PARTICIPATE IN A RESEARCH STUDY**

***Evaluation of Zimbabwe’s Accelerated***

***Prevention of Mother-to-Child Transmission of HIV Program***

**Authorization for the participation of this health facility**: You are making a decision whether or not to participate in this study on behalf of this health facility. Your signature indicates that you have read and understood the information provided above, have had all your questions answered, and have decided to participate.

Name of Health Facility

Health Facility Representative’s Name *(please print)*

_______________

Health Facility Representative’s Signature Date

_______________

Interviewer’s Name Date
